# Intranasal Oxytocin Combined With Social Skills Training for Schizophrenia: An Add-on Randomized Controlled Trial

**DOI:** 10.1093/schizbullopen/sgae022

**Published:** 2024-10-21

**Authors:** Liron Saporta-Wiesel, Ruth Feldman, Linda Levi, Michael Davidson, Shimon Burshtein, Ruben Gur, Orna Zagoory-Sharon, Revital Amiaz, Jinyoung Park, John M Davis, Mark Weiser

**Affiliations:** Zachai Department of Psychiatry, Sheba Medical Center, Ramat Gan, Israel; Department of Psychology, Ivcher School of Psychology, Reichman University, Hertzelia, Israel; Zachai Department of Psychiatry, Sheba Medical Center, Ramat Gan, Israel; Department of Psychiatry, Nicosia University School of Medicine, Cyprus; Department of Psychiatry, Beer Yaakov-Ness Ziona Mental Health Center, Israel; Department of Psychiatry, Perelman School of Medicine, University of Pennsylvania, Philadelphia, PA, USA; Department of Psychology, Ivcher School of Psychology, Reichman University, Hertzelia, Israel; Zachai Department of Psychiatry, Sheba Medical Center, Ramat Gan, Israel; Department of Psychiatry, Faculty of Medical and Health Sciences, Tel Aviv University, Tel Aviv, Israel; Department of Psychology and Neuroscience, Duke University, Durham, NC, USA; Department of Psychiatry, University of Illinois at Chicago, Chicago, IL, USA; Zachai Department of Psychiatry, Sheba Medical Center, Ramat Gan, Israel; Department of Psychiatry, Faculty of Medical and Health Sciences, Tel Aviv University, Tel Aviv, Israel

**Keywords:** oxytocin, RCT, schizophrenia, negative symptoms, social functioning

## Abstract

Some but not other studies on oxytocin for schizophrenia, particularly those using a higher dose, indicate that oxytocin improves negative symptoms of schizophrenia. We performed an add-on randomized controlled trial to examine the effect of high-dose oxytocin, social skills training, and their combination in the treatment of negative symptoms and social dysfunction in schizophrenia. Fifty-one subjects with schizophrenia were randomized, employing a two-by-two design: intranasal oxytocin (24 IU X3/day) or placebo, and social skills training or supportive psychotherapy, for 3 weeks. The primary outcome was the difference in the total score from baseline to end-of-study of a semi-structured interview which assessed patients’ social interactions in 3 scenarios: sharing a positive experience, sharing a conflict, and giving support when the experimenter shared a conflict. The interactions were scored using the Coding Interactive Behavior Manual (CIB), clinical symptoms were assessed with the Positive and Negative Syndrome Scale (PANSS). No significant difference was found between groups in the total CIB or PANSS scores. The majority of comparisons in the different social interactions between oxytocin and placebo, and between social skills training vs supportive psychotherapy, were also nonsignificant. Social skills training reduced blunted affect and gaze. In post-hoc analyses of the support interaction, oxytocin improved synchrony and decreased tension, while in the positive interaction it improved positive affect and avoidance. None of these findings remained significant when controlling for multiple comparisons. In conclusion, oxytocin did not influence participants’ social behavior, and was not effective in improving the symptoms of schizophrenia.

Clinicaltrials.gov Identifier: NCT01598623

## Introduction

Social disability is a hallmark of schizophrenia and considered part of the negative symptoms of the illness. Many patients have social abnormalities in early childhood,^1^ years before their first episode, which are predictive of the course and outcome of the illness.^2,3^ There are no effective pharmacological treatments for negative symptoms or social dysfunction.^4–6^

Oxytocin is a 9 amino-acid peptide synthesized in the hypothalamus, which acts both peripherally as a hormone and as a neurotransmitter in the brain. As a neurotransmitter, it has been found to play a key role in regulating mammalian, including human, social affiliation, such as sexual behavior and mother-infant and adult-adult pair-bond formation.^7–10^ Research in healthy humans has shown that intranasal administration of oxytocin impacts multiple social and emotional behaviors in a prosocial, pro-affiliative way, leading to an increase in trust, empathy, and eye contact.^11–17^ Studies on autism, a neurodevelopment disorder particularly linked with social dysfunction, have been equivocal.^18–23^

These observations suggest that oxytocin has the potential to be an effective prosocial intervention for schizophrenia as well. Initially, a few studies showed improvement in schizophrenia patients who received oxytocin,^24–28^ but subsequent studies failed to replicated these findings,^29–35^ meta-analyzed in Martins et al and Zheng et al.^22,36^ Those studies which found the clearest evidence for efficacy used a high dose of oxytocin, above 50 IU/day^24,26^; consequently our study evaluated oxytocin at 72 IU/day.

Some authors have hypothesized that combining medications with psychosocial intervention might help in the treatment of schizophrenia. Similar notions have been expressed regarding the use of cognition-enhancing medication, suggesting that patients who receive compounds that putatively enhance cognition should be treated with cognitive remediation at the same time.^37–41^ Ford and Young note that oxytocin increases the salience of social stimuli, increasing the signal-to-noise ratio of social information, and have specifically recommended that trials of oxytocin in autism be carried out combining behavioral therapy with oxytocin.^42^

Based on this concept, we administered oxytocin combined with social skills training focused on the key domains of social behavior impaired in schizophrenia, which are postulated to be improved by oxytocin. We used placebo as a control for the oxytocin and supportive psychotherapy as a control for the social skills training, and performed an add-on, randomized, double-blind, placebo-controlled study to examine the effects of intranasal oxytocin combined with social skills training in patients with schizophrenia patients.

Three main hypotheses guided the current study: (1) intranasal oxytocin at a high dose would be more beneficial compared to placebo in improving schizophrenia patients’ prosocial behavior and psychopathology; (2) social skills training would be more beneficial compared to supportive psychotherapy; (3) the combined effects of intranasal oxytocin together with the social skills training will be synergetic and will exceed the effects of each intervention on its own in improving prosocial behavior and negative symptoms.

## Methods

### Study Subjects

Participants were outpatients recruited from the Zachai Division of Psychiatry in the Sheba Medical Center in Ramat Gan, Israel and by advertisements in rehabilitation centers, notice boards, and the internet.

The inclusion criteria were current DSM-IV-TR diagnosis of schizophrenia or schizoaffective disorder confirmed by the Structured Clinical Interview for DSM-IV Axis I Disorders^43^; 18–65 years of age; Positive and Negative Syndrome Scale (PANSS) total score greater than 55; social dysfunction as defined by a score of 4 (moderate) or higher on at least 1 or more of the following PANSS negative items: emotional withdrawal, poor rapport, or passive-apathetic social withdrawal. Subjects had to be on the same antipsychotic medication for 2 weeks before randomization. Adjunctive treatment with other psychotropics was allowed, provided that patients had been on the medication for at least 2 weeks prior to entry into the screening phase of the study. Changes in concomitant medications, both psychiatric and other, were allowed and recorded.

The study was approved by the local IRB, and written informed consent was obtained from all participants. The study was registered on ClinicalTrials.gov (NCT01598623).

### Study Design

This study utilized a randomized, placebo-controlled, parallel, 2 × 2, double-blind, add-on design. Social skills training or supportive psychological therapy was administered by BA-level psychologists, 3 times a week, for the 3-week study period, for a total of 9 sessions. At baseline, a videotaped interview assessing the interaction between the subject and the experimenter was performed. Subjects were assessed once a week using the PANSS.^44^ At this weekly visit, and study medication inhalers were replaced. At the end of the 3 week period, subjects participated in a final, end-of-study visit during which all assessments were repeated.

### Study Medication

Subjects received daily intranasal oxytocin (Syntocinon Spray, Novartis) or intranasal placebo (matching vials containing saline) for a period of 3 weeks. Based on the previous studies showing efficacy,^24,27^ oxytocin was dosed at 24 IU 3 times a day. Subjects received 2 inhalers of study medication during their weekly visit and returned the used inhalers. Due to the potential for loss of drug dripping out through the nares if not properly administered, subjects were instructed how to self-administer the medication, spraying six 0.1 ml insufflations while tilting the head slightly back. Subjects were instructed to administer study medication 3 times a day: morning, noon, and evening, before meals. The daily dose was divided into 3 times a day since the literature shows that oxytocin’s levels go down back to baseline levels after approximately 7 h,^45,46^ thus the need to take repeated doses throughout the day.

On days when social skills training or psychotherapy was given, subjects were given 1 oxytocin dose 30 min prior to the session.

### Psychological Intervention

The social skills training was comprised of 9 sessions. The first 5 sessions were based on the relevant sessions from the Social Cognition and Interaction Training,^47^ which focus on understanding emotions. In these sessions, subjects were taught to define their emotions, and to link facial expressions to the emotions they portray. The last 4 sessions were from the Social Skills Training for Schizophrenia,^48^ focusing on starting conversation, listening to others, expressing positive as well as negative feelings. The training was administered individually rather than in a group format because different subjects were recruited at different times.

Supportive psychotherapy was administered as a control condition for the social skills training, to account for the nonspecific effects of therapist contact and interest, social interaction, and support. In these supportive psychotherapy sessions, subjects were encouraged to bring any particular topic to therapy, however, socially relevant topics were not addressed. Subjects underwent social skills training or supportive psychotherapy 3 times a week during the in-person clinical visits throughout the 3-week trial period. Subjects were instructed to administer study medication (oxytocin/placebo) 30 min before the session started. The psychological intervention was performed by 3 trained therapists, who followed a study-specific protocol for the social skills training. No formal training was performed.

### Social Interaction Measures

Before and after the 3-week oxytocin/placebo administration subjects were videotaped in 3 interaction paradigms developed for the adult version of the Coding Interactive Behavior (CIB),^49^ consistent with prior research on the CIB in adolescents and adults.^50–56^ These included: (1) a “positive interaction” in which subjects were asked to recall a positive event or experience that occurred in their lives; (2) a “conflict interaction” in which subjects were asked to talk about a conflict that occurred in their lives; (3) a “support interaction” in which the experimenter shared an incident that occurred in her life (conflict at work or with a family member). Subjects were asked to share their feelings and opinions in light of that story. Interactions were coded off-line by blinded raters, using the CIB,^49^ a well-validated system for coding dyadic interactions. The CIB is a global rating system of dyadic interactions that includes multiple scales, each coded from 1 (low) to 5 (high) and with versions for newborns, infants, children, adolescents, and adults. The system has shown good psychometric qualities on multiple studies across a wide range of ages, cultures, normative, and psychiatric conditions.^57^ The adult version of the CIB contains multiple scales that address the individual’s behavior (eg, gaze, expressed affect, and anxiety) and scales addressing the nature of the dialogue between patient and experimenter (eg, reciprocity, synchrony, and fluency). The parameters were rated on a scale of 1 (low) to 5 (high), with 0.5-point intervals. Reliability, rated as agreement of both raters on 10 paradigms averaged 91.74%.

### Data Analysis

The primary outcome measure was the change from baseline in the total score of the structured assessment of social interaction (CIB) in oxytocin, compared with placebo on all patients. Total score was the sum of all prosocial item, minus the sum of items that were not prosocial. Secondary outcome measures included change from baseline in the CIB total score in the oxytocin vs placebo groups, with and without social skills training, and social skills training vs supportive psychotherapy, with or without oxytocin. Changes from baseline in the total PANSS and PANSS subscales in all groups were additional secondary outcome measures.

A mixed-effect model with repeated measures (MMRM) was performed with participants as a random effect. The main effect and the interaction effect of medication (oxytocin = 1 vs placebo = 0) and psychological treatment (social skills training = 1 vs supportive psychotherapy = 0) were tested for every outcome. Specifically, (1) the medication effect (oxytocin vs placebo), (2) the psychological treatment effect (social skills training vs supportive psychotherapy), and (3) the effect of the combinations of oxytocin and psychological therapy (oxytocin/social skills training, oxytocin/supportive psychotherapy, placebo/social skills training, placebo/supportive psychotherapy) were examined. To prevent spurious associations from multiple comparisons, the *P* values were adjusted using Bonferroni correction.

Separate analyses were performed for the CIB variables individually. An exploratory factor analysis with the principal axis factoring method was used to identify the underlying dimensions of CIB items. A 5-factor structure was selected, explaining 67% of the total variance. Factor scores were calculated using regression-based weights for each factor and used in the MMRM as outcome variables. The factor analysis was done on the CIB for all the subjects to identify its dimensions. We used a MMRM to analyze the direct effect of oxytocin, the direct effect of social skills training, or their interactions. Similar analyses were performed for all secondary measures, including PANSS total score, PANSS subscales, and PANSS factor scores.^58^ R version 4.1.0 was used for the analysis.

## Results

There were no significant differences between the 4 groups on baseline demographic and clinical variables (table 1).

**Table 1.** Baseline Characteristics of Subjects

Measure	Oxytocin/Social Cognitive and Skills Training	Oxytocin/Supportive Psychotherapy	Placebo/Social Cognitive and Skills Training	Placebo/Supportive Psychotherapy	Overall	Statistic
Gender (male/female)	10/1	11/0	8/4	10/3	39/8	χ² (3) = 5.34 *P* = .14
Age (years ± SD)	39.45 ± 11.51	33.09 ± 9.72	37.83 ± 11.91	36.3 ± 8.33	36.68 ± 10.33	*F*(3,43) = 0.75 *P* = .52
Marital status (single/married/divorced)	9/2/0	8/2/1	10/0/2	13/0/0	40/4/3	χ² (6) = 8.74 *P* = .18
Education in years (mean ± SD)	10.45 ± 5.59	11.55 ± 4.15	11.67 ± 2.27	12 ± 3.91	11.45 ± 4.01	*F*(3,43) = 0.3 *P* = .82
Diagnosis (Schizophrenia/Schizoaffective)	10/1	10/1	12/0	9/4	41/6	χ² (3) = 5.8 *P* = .12
Age of onset	24 ± 8.79	22.5 ± 6.54	24.67 ± 7.11	23.85 ± 4.94	23.78 ± 6.72	*F*(3,43) = 0.19 *P* = .89
Duration of illness (mean ± SD)	15.45 ± 6.23	10.59 ± 10.28	13.16 ± 11.18	12.46 ± 7.63	12.9 ± 8.91	*F*(3,43) = 0.54 *P* = .65
PANSS Total at Baseline	64 ± 14.62	69.73 + 16.96	66.17 + 10.86	63.92 + 10.35	65.87 ± 13.06	*F*(3,43) = 0.47 *P* = .7
PANSS Positive at Baseline	12.73 ± 5.12	13.09 ± 4.94	12 ± 3.51	12.62 ± 3.15	12.6 ± 4.09	*F*(3,43) = 0.13 *P* = .93
PANSS Negative at Baseline	19.55 ± 5.64	22.73 ± 6.95	23 ± 4.11	20.54 ± 3.97	21.45 ± 5.26	*F*(3,43) = 1.18 *P* = .32
PANSS General Psychopathology at Baseline	31.73 ± 6.98	33.91 ± 9.02	31.17 ± 6.74	29.77 ± 5.38	31.55 ± 7	*F*(3,43) = 0.69 *P* = .55

**Fig. 1.**
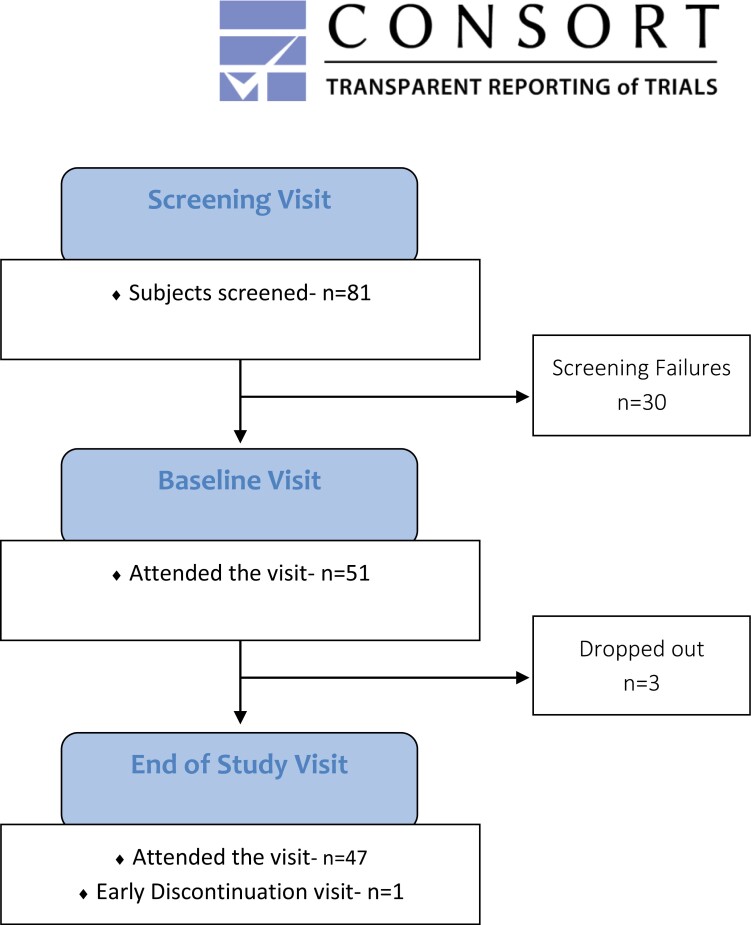
CONSORT diagram.

Eighty-one subjects with schizophrenia were screened, 51 were randomized, and 47 completed the 3-week study. One of the subjects who dropped out agreed to arrive for an early discontinuation visit. Since she completed 16 days of the study, these visits were included in the analysis, leaving 3 dropouts (see CONSORT diagram, figure 1).

Factor analysis of the CIB revealed 2 major factor and 3 minor factors (see web supplement—Section 2.1): Factor 1 (Synergy) had high loading with higher scores on synchrony (factor loading = 0.91), persistence (0.83), reciprocity (0.72), fluency (0.66), acknowledgment (0.64), and motivation (0.44), together with negative scores for constriction (0.73) and withdrawal (−0.42). This factor reflects the main prosocial dimension of the CIB. Factor 2 (Elaboration) had high loading on initiative (0.77), elaboration (0.79), alertness (0.59), patient leading the conversation (0.64), and motivation (0.32), with negative scores for blunted affect (−0.59) and the experimenter leading the conversation (−0.69). Factor 3 (Tension) had high loading on tension (0.86), as well as lower loading on anxiety (0.39), criticism (0.32), negative affect (0.34), and persistence (0.43), with less gaze (−0.45) and less reciprocity (−0.33). Factor 4 (Withdrawal) had high loading on withdrawal (0.55) and silence (0.42), and decreased intrusiveness (−0.67). Factor 5 (Positive affect and mismatch of affect) had high loading on positive (0.85) or mismatched affect (0.77).

### The Effect of Oxytocin and Social Skills Training on Total CIB Score

**Fig. 2.**
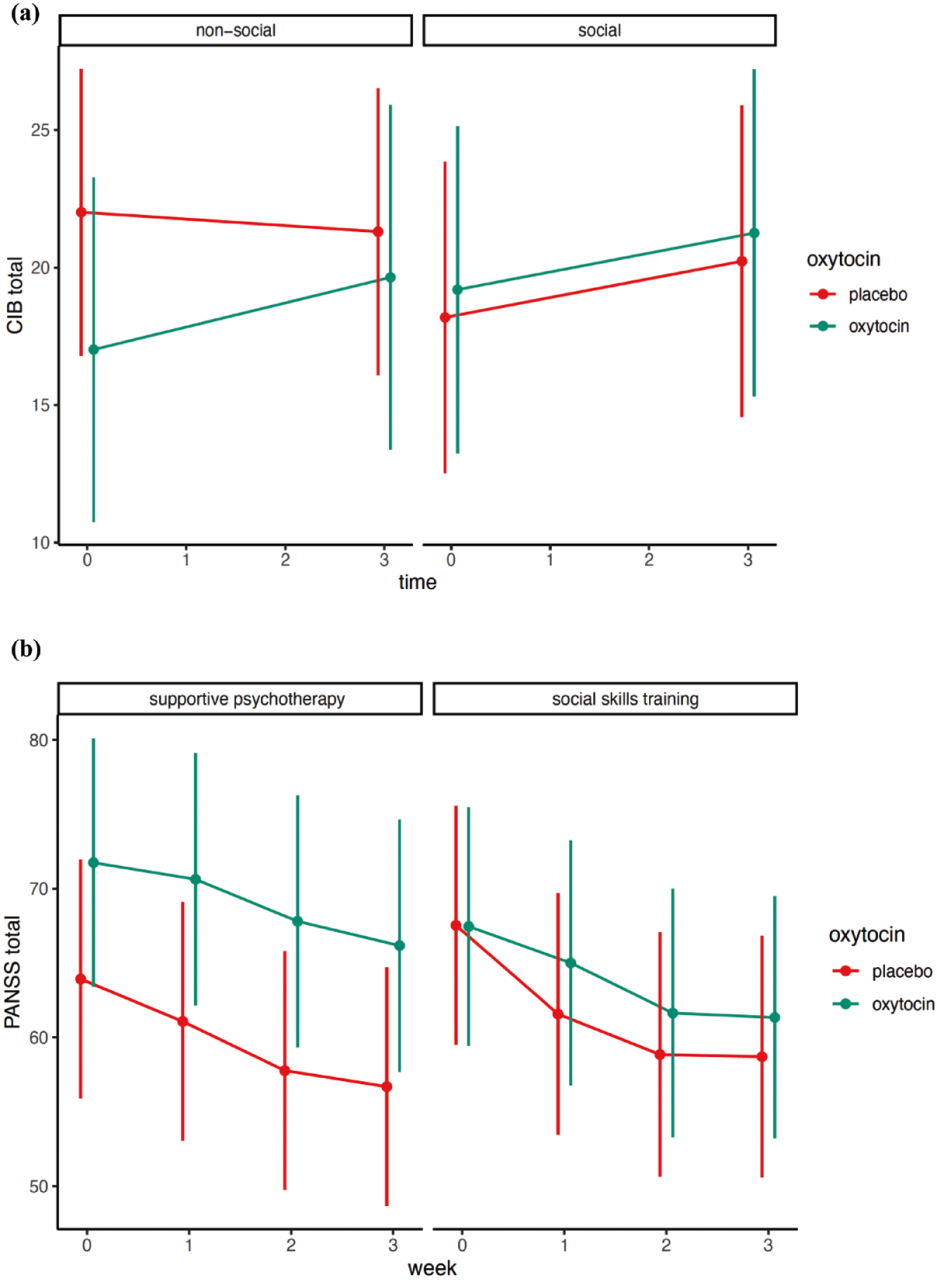

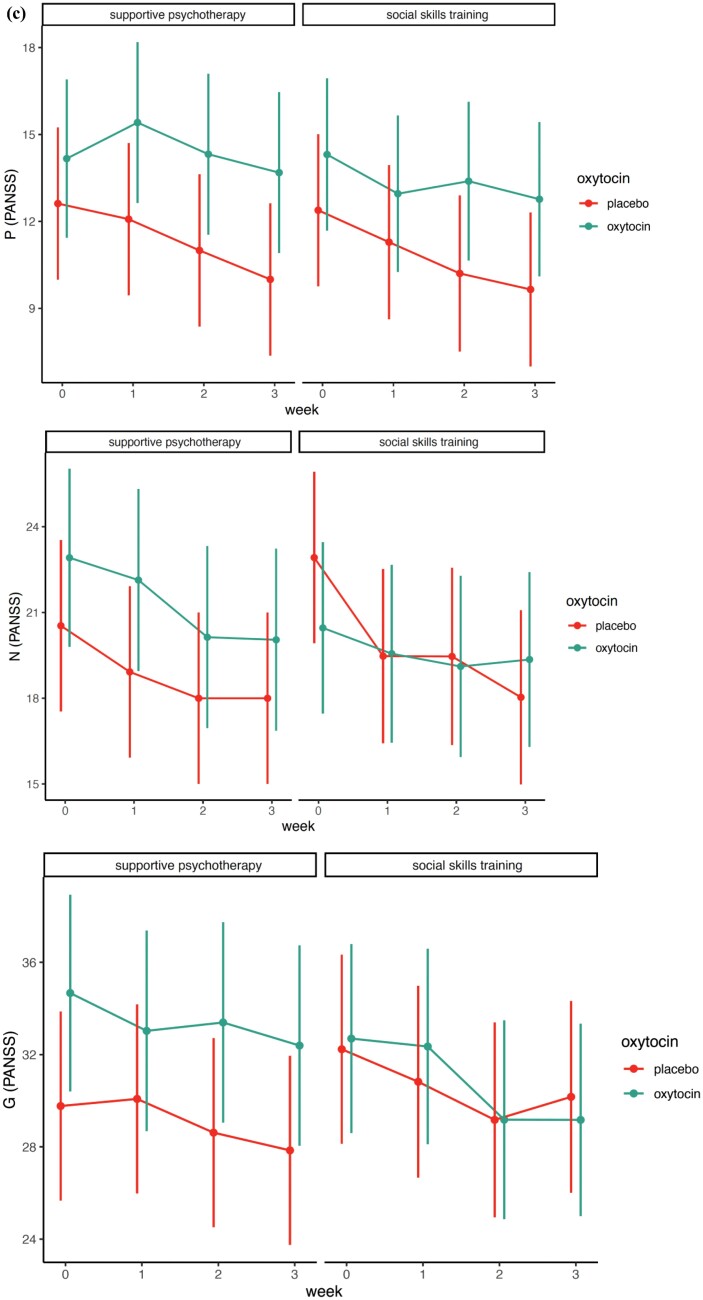
Interaction model with CIB total (a), PANSS total (b), and PANSS subscales (c) as outcomes.

A mixed-effect model was performed to test the effect of oxytocin, social skills training, or the combination of both treatments on the change of the total CIB score over time, our primary hypothesis. Although oxytocin alone (coefficient 0.59, *P* = .39), and social skills training alone (coefficient 0.46, *P* = .5) directionally increased total score, this difference was not statistically significant. Oxytocin increased CIB Total in the supportive psychotherapy group (coefficient −1.11, *P* = .43) but this was not significant. See figure 2, table 2, and web supplement.

**Table 2.** Two-way Complete Model for Oxytocin, Social Skills Training or Both for CIB and PANSS Outcomes

	1. Two-way Complete Model								
Predictors (Interaction With Time)	Effect of Oxytocin			Effect of Social Treatment			Interaction Effect of Oxytocin and Social Treatment		
Outcomes	Estimates (CIs)	*P*-value	*Q*-value	Estimates (CIs)	*P*-value	*Q*-value	Estimates (CIs)	*P*-value	Q-value
CIB Total Score									
Individual items									
Acknowledgment	0.12 (−0.10, 0.35)	.31	1.00	0.03 (−0.19, 0.24)	.82	1.00	−0.08 (−0.40, 0.25)	.65	1.00
Alertness	0.15 (−0.07, 0.37)	.20	1.00	0.17 (−0.04, 0.38)	.13	1.00	−0.33 (−0.64, −0.01)	.06	1.00
Anger	0.01 (−0.06, 0.08)	.80	1.00	0.02 (−0.05, 0.09)	.56	1.00	−0.05 (−0.16 , 0.05)	.36	1.00
Anxiety	0.05 (−0.06, 0.16)	.42	1.00	0.06 (−0.04, 0.17)	.27	1.00	−0.17 (−0.33, −0.01)	.05	1.00
Avoidance	0.07 (0.00, 0.14)	.05	1.00	0.07 (0.00, 0.13)	.05	1.00	−0.14 (−0.24, −0.05)	.01	.18
Blunt Affect	−0.04 (−0.16, 0.08)	.55	1.00	−0.13 (−0.24, −0.01)	.04	1.00	0.04 (−0.13, 0.22)	.65	1.00
Constriction	−0.13 (−0.30, 0.04)	.16	1.00	−0.08 (−0.24, 0.08)	.35	1.00	0.12 (−0.12, 0.36)	.36	1.00
Criticism	−0.08 (−0.18, 0.02)	.16	1.00	−0.06 (−0.16, 0.03)	.21	1.00	0.08 (−0.06, 0.23)	.28	1.00
Detachment	0.02 (−0.01, 0.06)	.26	1.00	0.01 (−0.02, 0.05)	.43	1.00	−0.01 (−0.06, 0.04)	.72	1.00
Elaboration	0.09 (−0.11, 0.30)	.40	1.00	−0.01 (−0.20, 0.19)	.96	1.00	−0.05 (−0.34, 0.24)	.75	1.00
Fluency	0.15 (−0.01, 0.31)	.08	1.00	0.09 (−0.06, 0.24)	.24	1.00	−0.15 (−0.37, 0.08)	.23	1.00
Gaze	−0.05 (−0.27, 0.16)	.63	1.00	0.19 (−0.01, 0.39)	.08	1.00	0.07 (−0.24, 0.37)	.67	1.00
Hostility	0.02 (−0.01, 0.06)	.18	1.00	−0.00 (−0.03, 0.03)	1.00	1.00	−0.04 (−0.09, 0.01)	.11	1.00
Initiation	0.24 (−0.06, 0.54)	.14	1.00	0.09 (−0.19, 0.38)	.54	1.00	−0.27 (−0.70, 0.16)	.23	1.00
Instrusiveness	−0.04 (−0.12, 0.04)	.31	1.00	0.01 (−0.07, 0.08)	.88	1.00	0.04 (−0.07, 0.15)	.52	1.00
Therapist leads	−0.01 (−0.17, 0.16)	.93	1.00	−0.00 (−0.16, 0.15)	1.00	1.00	−0.07 (−0.30, 0.16)	.58	1.00
Patient leads	0.05 (−0.09, 0.20)	.47	1.00	0.02 (−0.12, 0.16)	.78	1.00	−0.01 (−0.21, 0.20)	.96	1.00
Mismatch Affect	0.03 (−0.05, 0.11)	.44	1.00	0.03 (−0.04, 0.11)	.42	1.00	−0.06 (−0.17, 0.06)	.34	1.00
Motivation	0.05 (−0.13, 0.24)	.58	1.00	0.15 (−0.02, 0.32)	.11	1.00	−0.25 (−0.51, 0.01)	.08	1.00
Negative Affect	−0.19 (−0.39, 0.00)	.06	1.00	0.02 (−0.16, 0.21)	.80	1.00	0.13 (−0.15, 0.40)	.39	1.00
Positive Affect	0.13 (0.00, 0.26)	.06	1.00	0.06 (−0.06, 0.19)	.33	1.00	−0.18 (−0.37, 0.01)	.07	1.00
Persistence	−0.05 (−0.20, 0.09)	.49	1.00	−0.10 (−0.24, 0.04)	.19	1.00	0.03 (−0.18, 0.25)	.75	1.00
Reciprocity	0.05 (−0.10, 0.20)	.51	1.00	0.04 (−0.10, 0.18)	.62	1.00	−0.05 (−0.26, 0.16)	.63	1.00
Silence	0.00 (−0.10, 0.10)	.93	1.00	0.02 (−0.08, 0.11)	.73	1.00	0.03 (−0.11, 0.18)	.65	1.00
Synchrony	0.10 (−0.06, 0.27)	.25	1.00	0.06 (−0.09, 0.22)	.44	1.00	−0.12 (−0.36, 0.11)	.32	1.00
Tension	−0.11 (−0.24, 0.01)	.10	1.00	−0.10 (−0.22, 0.02)	.13	1.00	0.08 (−0.11, 0.26)	.43	1.00
Withdrawal	0.00 (−0.14, 0.14)	.99	1.00	−0.01 (−0.14 , 0.12)	.89	1.00	0.01 (−0.19, 0.21)	.95	1.00
Factors									
Synchrony	0.11 (−0.15, 0.37)	.41	1.00	0.09 (−0.15, 0.33)	.49	1.00	−0.15 (−0.52, 0.21)	.43	1.00
Initiation	0.14 (−0.09, 0.37)	.25	1.00	0.04 (−0.17, 0.26)	.70	1.00	−0.02 (−0.35, 0.31)	.90	1.00
Tension	−0.21 (−0.47, 0.04)	.12	.60	−0.15 (−0.40, 0.09)	.23	1.00	0.09 (−0.27, 0.46)	.63	1.00
Withdrawal	0.01 (−0.26, 0.28)	.95	1.00	−0.13 (−0.38, 0.13)	.35	1.00	0.08 (−0.30, 0.47)	.69	1.00
Positive Affect	0.28 (0.08, 0.49)	.01	.06	0.19 (−0.00, 0.39)	.07	.33	−0.36 (−0.66, −0.07)	.02	.11
	1. Two-way Complete Model								
Predictors (Interaction With Time)	Oxytocin			Social Treatment			Social Treatment × Oxytocin		
Outcomes	Estimates (CIs)	*P*-value	*Q*-value	Estimates (CIs)	*P*-value	*Q*-value	Estimates (CIs)	*P*-value	*Q*-value
PANSS Total	0.55 (−1.71, 2.80)	.63	1.00	−0.45 (−2.66, 1.75)	.69	1.00	0.25 (−2.93, 3.44)	.88	1.00
PANSS Positive Score	0.64 (−0.11, 1.39)	.10	.87	−0.04 (−0.77, 0.70)	.92	1.00	−0.14 (−1.20, 0.92)	.79	1.00
PANSS Negative Score	−0.21 (−1.22, 0.80)	.69	1.00	−0.64 (−1.63, 0.35)	.21	1.00	1.33 (−0.10, 2.76)	.07	.66
PANSS General Psychopathology Score	0.08 (−1.22, 1.37)	.91	1.00	−0.06 (−1.33, 1.21)	.93	1.00	−0.64 (−2.47, 1.19)	.50	1.00
Withdrawal Factor	−0.04 (−0.21, 0.12)	.61	1.00	−0.12 (−0.29, 0.04)	.14	1.00	0.23 (−0.00, 0.47)	.06	.52
Delusion Factor	0.09 (−0.02, 0.21)	.11	.99	0.02 (−0.09, 0.13)	.69	1.00	−0.04 (−0.20, 0.12)	.60	1.00
Anxiety Factor	−0.02 (−0.21, 0.17)	.82	1.00	0.00 (−0.19, 0.19)	1.00	1.00	−0.09 (−0.36, 0.18)	.53	1.00
Disorganization Factor	−0.00 (−0.13, 0.12)	.95	1.00	0.03 (−0.09, 0.16)	.61	1.00	−0.06 (−0.23, 0.13)	.55	1.00
Poor Control Factor	0.01 (−0.10, 0.12)	.84	1.00	−0.03 (−0.14, 0.08)	.64	1.00	−0.00 (−0.16, 0.16)	.99	1.00

(1. Two-way complete model: Outcome ~ Oxytocin * Social Treatment * Time).

### The Effect of Oxytocin and Social Skills Training on Individual CIB Variables and Factors

For each of the 27 CIB social interaction items and the CIB Factors, comparisons were made between oxytocin and placebo, between the social skills training and supportive psychotherapy, and their interactions. This is presented in table 2. The effect of oxytocin is presented in the left panel, the effect of social skills training in the next panel, and the interaction of the 2 treatments in the third panel. The outcome in the far-right panels reflects the direct outcome of oxytocin vs placebo, and social skills training vs supportive psychotherapy, evaluated without the interaction. These results are presented in graphs with more statistical information in the web supplement. When comparing oxytocin to placebo, summing the results over all 3 scenarios, oxytocin had no significant effects on the 2 major factors Synergy or Initiative, as well as the Tension and Withdrawal factors. Oxytocin improved the positive affect factor (coefficient 0.28, CI: 0.08–0.49, *P* = .01), but did so only in the supportive psychotherapy conditions, (the opposite of the predicted effects), (interaction coefficient −0.36, CI: −0.66 to −0.07, *P* = .02), (see web supplement 1.1.3, page 9.) Social skills training reduced blunted affect, regardless of oxytocin treatment (coefficient −0.13, CI: −0.24 to 0.01, *P* = .04).

Overall, oxytocin led to a decrease in the avoidance item, when combined with social skills training, and there was an increase in the avoidance item in the supportive psychotherapy interactions, coefficient −0.14, CI: −0.24 to −0.05, *P* = .01, see figure 2 and web supplement 1.2.5 page 17. Social skills training, regardless of oxytocin, led toward an improvement in gaze (estimate 0.22, *P* = .01).

When controlling for multiple comparisons, none of these findings remained significant.

### The Effect of Oxytocin and Social Skills Training in the 3 Social Interaction Conditions

Post-hoc analyses looking at the different CIB factors in the 3 (positive, conflicted, and supportive) social interactions separately (table 3) showed a few significant effects. In the positive social interaction, the positive affect factor improved in the oxytocin groups (coefficient 0.29, CI: 0.04−0.55, *P* = .03). In the conflict social interaction, the withdrawal factor (coefficient −0.33, CI: −0.57 to 0.08, *P* = .01) and the positive affect (coefficient 0.21, CI: 0.02–0.04, *P* = .04) showed improvement in the social skills training group compared to supportive psychotherapy. However, for those patients receiving social skills training together with oxytocin, withdrawal increased and positive affect decreased.

**Table 3.** Full Interaction Model for the Effect of Oxytocin, Social Skills Training or Both on the 5 Factors in the 3 Different Social Interaction Scenarios

	Full Interaction Model											
	Effect of Oxytocin				Effect of Social Treatment				Interaction Effect of Oxy and Social			
	coef	lower ci	upper ci	*P*-value	coef	lower ci	upper ci	*P*-value	coef	lower ci	upper ci	*P*-value
Conflict Interaction												
Synchrony	−0.02	−0.29	0.26	.92	0.05	−0.21	0.31	.71	0.12	−0.27	0.51	.56
Initiation	0.19	−0.07	0.45	.18	0.01	−0.23	0.26	.92	−0.09	−0.47	0.28	.63
Tension	−0.15	−0.47	0.16	.36	−0.16	−0.46	0.13	.3	0.12	−0.33	0.56	.62
Withdrawal	−0.16	−0.41	0.1	.25	−0.33	−0.57	−0.08	.01	0.39	0.03	0.76	.05
Positive Affect	0.14	−0.07	0.34	.21	0.21	0.02	0.4	.04	−0.45	−0.74	−0.16	.01
Positive interaction												
Synchrony	0.09	−0.23	0.41	.59	−0.1	−0.4	0.2	.54	−0.15	−0.6	0.3	.53
Initiation	0.01	−0.27	0.29	.96	0.16	−0.1	0.43	.25	0.07	−0.33	0.46	.75
Tension	−0.14	−0.42	0.15	.37	−0.14	−0.4	0.13	.34	0.04	−0.36	0.44	.85
Withdrawal	0.14	−0.22	0.5	.46	−0.06	−0.4	0.28	.74	−0.15	−0.66	0.36	.58
Positive Affect	0.29	0.04	0.55	.03	0.07	−0.16	0.31	.56	−0.32	−0.68	0.04	.1
Supportive interaction												
Synchrony	0.45	0.13	0.77	.01	0.19	−0.11	0.49	.24	−0.29	−0.74	0.16	.22
Initiation	0.25	−0.03	0.53	.1	0.08	−0.19	0.34	.58	−0.15	−0.55	0.24	.46
Tension	−0.39	−0.76	−0.03	.05	−0.25	−0.6	0.09	.16	0.54	0.02	1.05	.05
Criticism	−0.28	−0.61	0.05	.11	−0.18	−0.5	0.13	.28	0.15	−0.33	0.62	.56
Positive Affect	0.13	−0.19	0.45	.45	−0.13	−0.43	0.18	.43	−0.07	−0.53	0.39	.77

In the supportive interaction, oxytocin improved the synchrony factor (coefficient 0.45, CI: 0.13–0.77, *P* = .01), and decreased the tension factor (coefficient −0.39, CI: −0.76 to −0.03, *P* = .05). However, the opposite effect is seen for patients receiving oxytocin together with the social skills training (coefficient 0.54, CI: 0.02–1.05, *P* = .05).

All *P* values were not significant when controlling for multiple comparisons.

### The Effect of Oxytocin and Social Skills Training on PANSS Scores

We evaluated the interaction effects of oxytocin and social skills training on the PANSS total score, its subscales, and the 5 PANSS factors, to test the hypothesis that oxytocin and social skills training potentiated each other. No statistically significant effects were reported for both the main interventions (oxytocin or social skills training) and the interaction of the 2 on any PANSS variable. The only significant main effect of oxytocin was the worsening of positive symptoms (coefficient estimate 0.56, 95% CI: 0.03–1.09, *P* = .04), when directly evaluated since the interactions was not significant. However, when correcting for multiple comparisons, this finding did not remain significant (table 2 and figure 2).

## Discussion

This was an exploratory study, using the CIB coding instrument to test the effect of oxytocin, social skills training, and the combination of the 2 on patients with schizophrenia. This instrument has not been previously used in schizophrenia, therefore we looked at the total score, its factors, and individual items to explore the possible effect of oxytocin. Overall, oxytocin failed to improve social interactions and overall psychopathology, as seen in the (PANSS) analyses when we controlled for multiple comparisons.

### Social Interactions

Before controlling for multiple comparisons, oxytocin did improve avoidant behavior. This is consistent with the literature and specifically with the theory of Kemp and Guastella,^59^ who suggest that oxytocin decreases avoidance and withdrawal. This finding is also supported by studies on rodents,^60^ borderline personality disorder,^61^ and social anxiety.^62^

Very interesting and relevant for our study is the work of Heinrichs et al^63^ which showed that the combination of intranasal administration of oxytocin and social support reduces both anxiety and neuroendocrine stress reactivity in healthy men. Although the social support given in the Heinrichs study is not identical to the social skills training given in our study, it suggests that in these situations when support is needed, oxytocin may be relevant.

Our study is consistent with several studies that failed to find oxytocin more effective than placebo on social functioning.^31,35,64–68^

### Symptoms

We found no effect of high-dose oxytocin on clinical symptoms measured by the PANSS. Meta-analyses found oxytocin to be no better than placebo on PANSS total score,^17,22,69^ a finding consistent with other reviews.^70^ But several of the studies that did find oxytocin effective on the PANSS used high doses,^24,26,27^ which is why we used this high dose, but with no improvement. Furthermore, we found a hint of greater improvements for the placebo group in the PANSS positive subscale.

A meta-analysis of randomized, placebo-controlled trials examined the efficacy of oxytocin in schizophrenia and found that oxytocin was superior to placebo for the PANSS general psychopathology scale, and was not different from placebo for PANSS total symptoms, positive or negative symptoms.^69^ However, when the researchers removed the study of Davis et al,^28^ due to intermittent administration of drug, the meta-analysis found improvement in negative symptoms. A meta-analysis of Sabe suggested that oxytocin might benefit negative symptoms, particularly if given at higher doses.^30^ Our findings failed to find oxytocin to significantly alter negative symptoms, particularly in the social skill training group.

Two studies similar to ours combined psychotherapy and oxytocin. Davis et al^28^ and Cacciotti-Saija et al,^33^ administered oxytocin and social skills training to schizophrenia patients for a period of 6 weeks. Both studies did not find improvements in positive symptoms.

### Strengths and Limitations

Although we had a relative small sample size, this was the third-largest study on oxytocin in schizophrenia. Studies with larger sample sizes might yield more information on oxytocin’s potential as a therapeutic molecule. We cannot rule out that a dose higher than ours might be efficacious. Social functioning is complex, and we cannot rule out the possibility of oxytocin effecting some aspects not captured by our measures.

We administered oxytocin chronically for 3 weeks and at the end of study visit we administered the last dose 30 min before the assessment of social interactions. For that reason, we could not separate the influence of daily administration over the 3 week study period, from that of the last single administration. Scheduling an additional visit at least 3 days after the cessation of treatment, would have enabled us to differentiate between these possibilities. Oxytocin was administered 30 min before the social skills training or psychotherapy so that these treatments occurred when Oxytocin plasma and brain levels may have been elevated. However, previous studies have failed to find a difference between intranasal oxytocin and placebo among schizophrenia patients, even 20 h after the last oxytocin dose.^71^

An additional limitation of this study is the inclusion of subjects who were stable on antipsychotics for 2 weeks prior to randomization. This is a relatively short interval of medication stability, and might have led to the inclusion of patients who might not have been clinically stable, with potentially increased variability and reduced ability to discern a therapeutic effect of the experimental intervention.

The current study had a gender imbalance, with 38 males and 8 females, thus limiting the ability to make any gender-specific inferences. Also, tolerance and compliance were not measured and might have affected outcomes. However, other studies on intranasal oxytocin did not report significant side effects.

A major strength of this study was the inclusion of a “real-life” outcome measure, which has never used in schizophrenia studies testing oxytocin before. It is somewhat similar to another study, which assessed social skills in schizophrenia patients during role-playing after 6 weeks of oxytocin administration.^25^ That study also found no significant change in the quality of the interaction during rule playing in the oxytocin group.

### Conclusions

In conclusion, this study’s findings do not support the use of oxytocin, social skills training, or the combination of both as a treatment for schizophrenia.

## Supplementary Material

sgae022_suppl_Supplementary_Material

## References

[1] Weinberger D. Neurodevelopmental perspectives on schizophrenia. In: Psychopharmacology: The Fourth Generation of Progress. New York: Raven Press, Ltd.; 1995:1171–1183.

[2] Johnstone EC , Macmillan JF, Frith CD, Benn DK, Crow TJ. Further investigation of the predictors of outcome following first schizophrenic episodes. Br J Psychiatry. 1990;157(2):182–189. 2224368 10.1192/bjp.157.2.182

[3] Marwick K , Hall J. Social cognition in schizophrenia: a review of face processing. Br Med Bull. 2008;88(1):43–58. 18812413 10.1093/bmb/ldn035

[4] Carpenter WT , Koenig JI. The evolution of drug development in schizophrenia: past issues and future opportunities. Neuropsychopharmacology. 2008;33(9):2061–2079. 18046305 10.1038/sj.npp.1301639 PMC2575138

[5] Kirkpatrick B , Fenton WS, Carpenter WT, Marder SR. The NIMH-MATRICS consensus statement on negative symptoms. Schizophr Bull. 2006;32(2):214–219. 16481659 10.1093/schbul/sbj053 PMC2632223

[6] Krause M , Zhu Y, Huhn M, et al Antipsychotic drugs for patients with schizophrenia and predominant or prominent negative symptoms: a systematic review and meta-analysis. Eur Arch Psychiatry Clin Neurosci. 2018;268(7):625–639. 29368205 10.1007/s00406-018-0869-3

[7] Argiolas A , Gessa GL. Central functions of oxytocin. Neurosci Biobehav Rev. 1991;15(2):217–231. 1852313 10.1016/s0149-7634(05)80002-8

[8] Insel TR. Oxytocin—a neuropeptide for affiliation: evidence from behavioral, receptor autoradiographic, and comparative studies. Psychoneuroendocrinology. 1992;17(1):3–35. 1319071 10.1016/0306-4530(92)90073-g

[9] McCarthy MM , Altemus M. Central nervous system actions of oxytocin and modulation of behavior in humans. Mol Med Today. 1997;3(6):269–275. 9211418 10.1016/S1357-4310(97)01058-7

[10] Insel TR. The challenge of translation in social neuroscience: a review of oxytocin, vasopressin, and affiliative behavior. Neuron. 2010;65(6):768–779. 20346754 10.1016/j.neuron.2010.03.005 PMC2847497

[11] Carter CS , Kenkel WM, MacLean EL, et al Is oxytocin “nature’s medicine?” Pharmacol Rev. 2020;72(4):829–861. 32912963 10.1124/pr.120.019398 PMC7495339

[12] Jurek B , Neumann ID. The oxytocin receptor: from intracellular signaling to behavior. Physiol Rev. 2018;98(3):1805–1908. 29897293 10.1152/physrev.00031.2017

[13] Feldman R. The neurobiology of human attachments. Trends Cogn Sci. 2017;21(2):80–99. 28041836 10.1016/j.tics.2016.11.007

[14] Guastella AJ , Mitchell PB, Dadds MR. Oxytocin increases gaze to the eye region of human faces. Biol Psychiatry. 2008;63(1):3–5. 17888410 10.1016/j.biopsych.2007.06.026

[15] Hurlemann R , Patin A, Onur OA, et al Oxytocin enhances amygdala-dependent, socially reinforced learning and emotional empathy in humans. J Neurosci. 2010;30(14):4999–5007. 20371820 10.1523/JNEUROSCI.5538-09.2010 PMC6632777

[16] Kosfeld M , Heinrichs M, Zak PJ, Fischbacher U, Fehr E. Oxytocin increases trust in humans. Nature. 2005;435(7042):673–676. 15931222 10.1038/nature03701

[17] Yang X , Wang W, Wang Y, Wang X. A meta-analysis of hormone administration effects on cooperative behaviours: oxytocin, vasopressin, and testosterone. Neurosci Biobehav Rev. 2021;126:430. 33819546 10.1016/j.neubiorev.2021.03.033

[18] Andari E , Duhamel J-R, Zalla T, Herbrecht E, Leboyer M, Sirigu A. Promoting social behavior with oxytocin in high-functioning autism spectrum disorders. Proc Natl Acad Sci USA. 2010;107(9):4389–4394. 20160081 10.1073/pnas.0910249107 PMC2840168

[19] Guastella AJ , Einfeld SL, Gray KM, et al Intranasal oxytocin improves emotion recognition for youth with autism spectrum disorders. Biol Psychiatry. 2010;67(7):692–694. 19897177 10.1016/j.biopsych.2009.09.020

[20] Hollander E , Bartz J, Chaplin W, et al Oxytocin increases retention of social cognition in autism. Biol Psychiatry. 2007;61(4):498–503. 16904652 10.1016/j.biopsych.2006.05.030

[21] Huang Y , Huang X, Ebstein RP, Yu R. Intranasal oxytocin in the treatment of autism spectrum disorders: a multilevel meta-analysis. Neurosci Biobehav Rev. 2021;122:18–27. 33400920 10.1016/j.neubiorev.2020.12.028

[22] Martins D , Paduraru M, Paloyelis Y. Heterogeneity in response to repeated intranasal oxytocin in schizophrenia and autism spectrum disorders: a meta-analysis of variance. Br J Pharmacol. 2022;179(8):1525–1543. 33739447 10.1111/bph.15451

[23] Yamasue H , Okada T, Munesue T, et al Effect of intranasal oxytocin on the core social symptoms of autism spectrum disorder: a randomized clinical trial. Mol Psychiatry. 2020;25(8):1849–1858. 29955161 10.1038/s41380-018-0097-2

[24] Feifel D , Macdonald K, Nguyen A, et al Adjunctive intranasal oxytocin reduces symptoms in schizophrenia patients. Biol Psychiatry. 2010;68(7):678–680. 20615494 10.1016/j.biopsych.2010.04.039

[25] Gibson CM , Penn DL, Smedley KL, Leserman J, Elliott T, Pedersen CA. A pilot six-week randomized controlled trial of oxytocin on social cognition and social skills in schizophrenia. Schizophr Res. 2014;156(2–3):261–265. 24799299 10.1016/j.schres.2014.04.009

[26] Modabbernia A , Rezaei F, Salehi B, et al Intranasal oxytocin as an adjunct to risperidone in patients with schizophrenia. CNS Drugs. 2013;27(1):57–65. 23233269 10.1007/s40263-012-0022-1

[27] Pedersen CA , Gibson CM, Rau SW, et al Intranasal oxytocin reduces psychotic symptoms and improves theory of Mind and social perception in schizophrenia. Schizophr Res. 2011;132(1):50–53. 21840177 10.1016/j.schres.2011.07.027

[28] Davis MC , Green MF, Lee J, et al Oxytocin-augmented social cognitive skills training in schizophrenia. Neuropsychopharmacology. 2014;39(9):2070–2077. 24637803 10.1038/npp.2014.68 PMC4104336

[29] Bürkner P-C , Williams DR, Simmons TC, Woolley JD. Intranasal oxytocin may improve high-level social cognition in schizophrenia, but not social cognition or neurocognition in general: a multilevel Bayesian meta-analysis. Schizophr Bull. 2017;43(6):1291–1303. 28586471 10.1093/schbul/sbx053 PMC5737621

[30] Sabe M , Zhao N, Crippa A, Strauss GP, Kaiser S. Intranasal oxytocin for negative symptoms of schizophrenia: systematic review, meta-analysis, and dose-response meta-analysis of randomized controlled trials. Int J Neuropsychopharmacol. 2021;24(8):601–614. 33890987 10.1093/ijnp/pyab020 PMC8378078

[31] Jarskog LF , Pedersen CA, Johnson JL, et al A 12-week randomized controlled trial of twice-daily intranasal oxytocin for social cognitive deficits in people with schizophrenia. Schizophr Res. 2017;185:88–95. 28094169 10.1016/j.schres.2017.01.008 PMC5474129

[32] Buchanan RW , Kelly DL, Weiner E, et al A randomized clinical trial of oxytocin or galantamine for the treatment of negative symptoms and cognitive impairments in people with schizophrenia. J Clin Psychopharmacol. 2017;37(4):394–400. 28590362 10.1097/JCP.0000000000000720 PMC5484721

[33] Cacciotti-Saija C , Langdon R, Ward PB, et al A double-blind randomized controlled trial of oxytocin nasal spray and social cognition training for young people with early psychosis. Schizophr Bull. 2015;41(2):483–493. 24962607 10.1093/schbul/sbu094 PMC4332939

[34] Dagani J , Sisti D, Abelli M, et al Do we need oxytocin to treat schizophrenia? A randomized clinical trial. Schizophr Res. 2016;172(1–3):158–164. 26883950 10.1016/j.schres.2016.02.011

[35] Lee MR , Wehring HJ, McMahon RP, et al Effects of adjunctive intranasal oxytocin on olfactory identification and clinical symptoms in schizophrenia: results from a randomized double blind placebo controlled pilot study. Schizophr Res. 2013;145(1–3):110–115. 23415472 10.1016/j.schres.2013.01.001 PMC4125132

[36] Zheng W , Zhu X-M, Zhang Q-E, et al Adjunctive intranasal oxytocin for schizophrenia: a meta-analysis of randomized, double-blind, placebo-controlled trials. Schizophr Res. 2019;206:13–20. 30573406 10.1016/j.schres.2018.12.007

[37] Scheidtmann K , Fries W, Müller F, Koenig E. Effect of levodopa in combination with physiotherapy on functional motor recovery after stroke: a prospective, randomised, double-blind study. Lancet. 2001;358(9284):787–790. 11564483 10.1016/S0140-6736(01)05966-9

[38] Norberg MM , Krystal JH, Tolin DF. A meta-analysis of D-cycloserine and the facilitation of fear extinction and exposure therapy. Biol Psychiatry. 2008;63(12):1118–1126. 18313643 10.1016/j.biopsych.2008.01.012

[39] Chasson GS , Buhlmann U, Tolin DF, et al Need for speed: evaluating slopes of OCD recovery in behavior therapy enhanced with d-cycloserine. Behav Res Ther. 2010;48(7):675–679. 20362975 10.1016/j.brat.2010.03.007

[40] Montero-Odasso M , Almeida QJ, Burhan AM, et al SYNERGIC TRIAL (SYNchronizing Exercises, Remedies in Gait and Cognition) a multi-Centre randomized controlled double blind trial to improve gait and cognition in mild cognitive impairment. BMC Geriatr. 2018;18(1):1–15. 29661156 10.1186/s12877-018-0782-7 PMC5902955

[41] Lauriello J , Lenroot R, Bustillo JR. Maximizing the synergy between pharmacotherapy and psychosocial therapies for schizophrenia. Psychiatr Clin North Am. 2003;26(1):191–211. 12683266 10.1016/s0193-953x(02)00017-5

[42] Ford CL , Young LJ. Refining oxytocin therapy for autism: context is key. Nat Rev Neurol. 2022;18(2):67–68. 34880473 10.1038/s41582-021-00602-9 PMC8816821

[43] First MB , Pincus HA. The DSM-IV text revision: rationale and potential impact on clinical practice. Psychiatr Serv. 2002;53(3):288–292. 11875221 10.1176/appi.ps.53.3.288

[44] Kay SR , Fiszbein A, Opler LA. The Positive and Negative Syndrome Scale (PANSS) for schizophrenia. Schizophr Bull. 1987;13(2):261–276. 3616518 10.1093/schbul/13.2.261

[45] Weisman O , Zagoory-Sharon O, Feldman R. Intranasal oxytocin administration is reflected in human saliva. Psychoneuroendocrinology. 2012;37(9):1582–1586. 22436536 10.1016/j.psyneuen.2012.02.014

[46] Van IJzendoorn MH , Bhandari R, Van der Veen R, Grewen KM, Bakermans-Kranenburg MJ. Elevated salivary levels of oxytocin persist more than 7 h after intranasal administration. Front Neurosci. 2012;6:174. 23233832 10.3389/fnins.2012.00174 PMC3516702

[47] Penn DL , Roberts DL, Combs D, Sterne A. Best practices: the development of the social cognition and interaction training program for schizophrenia spectrum disorders. Psychiatr Serv. 2007;58(4):449–451. 17412842 10.1176/ps.2007.58.4.449

[48] Bellack AS. Social Skills Training for Schizophrenia: A Step-by-Step Guide. New York: Guilford Press; 2004.

[49] Feldman R. Coding interactive behavior manual. Unpublished manual; 1998.

[50] Levy J , Goldstein A, Feldman R. Perception of social synchrony induces mother–child gamma coupling in the social brain. Soc Cogn Affect Neurosci. 2017;12:1036–1046. 28402479 10.1093/scan/nsx032 PMC5490671

[51] Levy J , Goldstein A, Influs M, Masalha S, Zagoory-Sharon O, Feldman R. Adolescents growing up amidst intractable conflict attenuate brain response to pain of outgroup. Proc Natl Acad Sci USA. 2016;113(48):13696–13701. 27849588 10.1073/pnas.1612903113 PMC5137744

[52] Lebowitz ER , Silverman WK, Martino AM, Zagoory-Sharon O, Feldman R, Leckman JF. Oxytocin response to youth–mother interactions in clinically anxious youth is associated with separation anxiety and dyadic behavior. Depress Anxiety. 2017;34:127. 28052452 10.1002/da.22585 PMC5503301

[53] Schneiderman I , Kanat-Maymon Y, Ebstein RP, Feldman R. Cumulative risk on the oxytocin receptor gene (OXTR) underpins empathic communication difficulties at the first stages of romantic love. Soc Cogn Affect Neurosci. 2014;9(10):1524–1529. 23974948 10.1093/scan/nst142 PMC4187267

[54] Schneiderman I , Kanat-Maymon Y, Zagoory-Sharon O, Feldman R. Mutual influences between partners’ hormones shape conflict dialog and relationship duration at the initiation of romantic love. Social Neurosci. 2014;9(4):337–351. 10.1080/17470919.2014.893925 24579960

[55] Feldman R , Rosenthal Z, Eidelman AI. Maternal-preterm skin-to-skin contact enhances child physiologic organization and cognitive control across the first 10 years of life. Biol Psychiatry. 2014;75(1):56–64. 24094511 10.1016/j.biopsych.2013.08.012

[56] Schneiderman I , Zagoory-Sharon O, Leckman JF, Feldman R. Oxytocin during the initial stages of romantic attachment: relations to couples’ interactive reciprocity. Psychoneuroendocrinology. 2012;37(8):1277–1285. 22281209 10.1016/j.psyneuen.2011.12.021 PMC3936960

[57] Feldman R. Oxytocin and social affiliation in humans. Horm Behav. 2012;61(3):380–391. 22285934 10.1016/j.yhbeh.2012.01.008

[58] Davis JM , Chen N. The effects of olanzapine on the 5 dimensions of schizophrenia derived by factor analysis: combined results of the North American and international trials. J Clin Psychiatry. 2001;62(10):757–771. 11816864 10.4088/jcp.v62n1003

[59] Kemp AH , Guastella AJ. Oxytocin: prosocial behavior, social salience, or approach-related behavior? Biol Psychiatry. 2010;67(6):e33–4; author reply e35. 20060102 10.1016/j.biopsych.2009.11.019

[60] Lukas M , Toth I, Reber SO, Slattery DA, Veenema AH, Neumann ID. The neuropeptide oxytocin facilitates pro-social behavior and prevents social avoidance in rats and mice. Neuropsychopharmacology. 2011;36(11):2159–2168. 21677650 10.1038/npp.2011.95 PMC3176581

[61] Brüne M , Ebert A, Kolb M, Tas C, Edel MA, Roser P. Oxytocin influences avoidant reactions to social threat in adults with borderline personality disorder. Hum Psychopharmacol. 2013;28(6):552–561. 23950057 10.1002/hup.2343

[62] Radke S , Roelofs K, De Bruijn ER. Acting on anger social anxiety modulates approach-avoidance tendencies after oxytocin administration. Psychol Sci. 2013;24:0956797612472682. 10.1177/0956797612472682 23737083

[63] Heinrichs M , Baumgartner T, Kirschbaum C, Ehlert U. Social support and oxytocin interact to suppress cortisol and subjective responses to psychosocial stress. Biol Psychiatry. 2003;54(12):1389–1398. 14675803 10.1016/s0006-3223(03)00465-7

[64] Lee MR , Wehring HJ, McMahon RP, et al The effect of intranasal oxytocin on measures of social cognition in schizophrenia: a negative report. J Psychiatry Brain Sci. 2019;4(1):e190001. 10.20900/jpbs.20190001 PMC6485966 31037274

[65] Dwyer KR , Andrea AM, Savage CL, et al A randomized clinical trial of oxytocin or galantamine in schizophrenia: assessing the impact on behavioral, lexical, and self-report indicators of social affiliation. Schizophr Bull Open. 2020;1(1):sgaa001. 32803156 10.1093/schizbullopen/sgaa001 PMC7418868

[66] Cohen AS , Mitchell KR, Strauss GP, et al The effects of oxytocin and galantamine on objectively-defined vocal and facial expression: data from the CIDAR study. Schizophr Res. 2017;188:141–143. 28130004 10.1016/j.schres.2017.01.028 PMC5524598

[67] Buchanan RW , Kelly DL, Strauss GP, et al Combined oxytocin and CBSST for social function in people with schizophrenia. J Clin Psychopharmacol. 2021;41(3):236–243. 33783399 10.1097/JCP.0000000000001397 PMC8887701

[68] Browne J , Harvey PD, Buchanan RW, et al A longitudinal examination of real-world sedentary behavior in adults with schizophrenia-spectrum disorders in a clinical trial of combined oxytocin and cognitive behavioral social skills training. Behav Sci. 2022;12(3):60. 35323379 10.3390/bs12030060 PMC8945120

[69] Oya K , Matsuda Y, Matsunaga S, Kishi T, Iwata N. Efficacy and safety of oxytocin augmentation therapy for schizophrenia: an updated systematic review and meta-analysis of randomized, placebo-controlled trials. Eur Arch Psychiatry Clin Neurosci. 2015;266:439–450. 26303414 10.1007/s00406-015-0634-9

[70] Heringa SM , Begemann MJ, Goverde AJ, Sommer IE. Sex hormones and oxytocin augmentation strategies in schizophrenia: a quantitative review. Schizophr Res. 2015;168:603–613. 25914107 10.1016/j.schres.2015.04.002

[71] Busnelli M , Dagani J, De Girolamo G, et al Unaltered oxytocin and vasopressin plasma levels in patients with schizophrenia after 4 months of daily treatment with intranasal oxytocin. J Neuroendocrinol. 2016;28(4). 10.1111/jne.12359 26715485

