# Supplementary material for: Intranasal Oxytocin Combined With Social Skills Training for Schizophrenia: An Add-on Randomized Controlled Trial
Source: Schizophr Bull Open. 2024 Oct 21;5(1):sgae022. doi: 10.1093/schizbullopen/sgae022 (PMC11535855; doi:10.1093/schizbullopen/sgae022)
Supplement: sgae022_suppl_Supplementary_Material [file sgae022_suppl_supplementary_material.docx]

Web Supplement

# Contents

1. [**The oxytocin–social-treatment full interaction models**](#_bookmark0) **7**
   1. [**The oxytocin–social-treatment full interaction models: the five social interaction factors**](#_bookmark1)

[**as the outcomes**](#_bookmark1) **7**

- - 1. [The full interaction model with Factor 1 (synchrony) total as the outcome](#_bookmark2) 8
    2. [The full interaction model with Factor 2 (initiation) total as the outcome](#_bookmark3) 9
    3. [The full interaction model with Factor 3 (tension) total as the outcome](#_bookmark4) 10
    4. [The full interaction model with Factor 4 (withdrawal) total as the outcome](#_bookmark5) 11
    5. [The full interaction model with Factor 5 (Positive affect) total as the outcome](#_bookmark6) 12
    6. [The full interaction model with Factor 1 (synchrony) Conflict Interaction as the outcome](#_bookmark7) 13
    7. [The full interaction model with Factor 2 (initiation) Conflict Interaction as the outcome](#_bookmark8) 14
    8. [The full interaction model with Factor 3 (tension) Conflict Interaction as the outcome](#_bookmark9) 15
    9. [The full interaction model with Factor 4 (withdrawal) Conflict Interaction as the outcome](#_bookmark10) 16
    10. [The full interaction model with Factor 5 (Positive affect) Conflict Interaction as the outcome](#_bookmark11) 17
    11. [The full interaction model with Factor 1 (synchrony) Positive Interaction as the outcome](#_bookmark12) 18
    12. [The full interaction model with Factor 2 (initiation) Positive Interaction as the outcome](#_bookmark13) 19
    13. [The full interaction model with Factor 3 (tension) Positive Interaction as the outcome](#_bookmark14) 20
    14. [The full interaction model with Factor 4 (withdrawal) Positive Interaction as the outcome](#_bookmark15) . 21
    15. [The full interaction model with Factor 5 (Positive affect) Positive Interaction as the outcome](#_bookmark16) 22
    16. [The full interaction model with Factor 1 (synchrony) Supportive interaction as the outcome](#_bookmark17) 23
    17. [The full interaction model with Factor 2 (initiation) Supportive interaction as the outcome](#_bookmark18) . 24
    18. [The full interaction model with Factor 3 (EmpathEmoEx) Supportive interaction as the](#_bookmark19)

[outcome](#_bookmark19) 25

- - 1. [The full interaction model with Factor 4 (Positive affect) Supportive interaction as the outcome](#_bookmark20) 26
    2. [The full interaction model with Factor 5 (Critisism) Supportive interaction as the outcome](#_bookmark21) . 27
    3. [The full interaction model with CIB total as the outcome](#_bookmark22) 28
  1. [The oxytocin–social-treatment full interaction models: the individual social interaction](#_bookmark23) [scores as the outcomes](#_bookmark23) 29
     1. [The full interaction model with Acknowledgment as the outcome](#_bookmark24) 30
     2. [The full interaction model with Alert as the outcome](#_bookmark25) 31
     3. [The full interaction model with Anger as the outcome](#_bookmark26) 32
     4. [The full interaction model with Anxiety as the outcome](#_bookmark27) 33
     5. [The full interaction model with Avoidance as the outcome](#_bookmark28) 34
     6. [The full interaction model with Blatant affect as the outcome](#_bookmark29) 35
     7. [The full interaction model with Constricted as the outcome](#_bookmark30) 36
     8. [The full interaction model with Criticism as the outcome](#_bookmark31) 37
     9. [The full interaction model with Detachment as the outcome](#_bookmark32) 38
     10. [The full interaction model with Elaboration as the outcome](#_bookmark33) 39
     11. [The full interaction model with Fluency as the outcome](#_bookmark34) 40
     12. [The full interaction model with Gaze as the outcome](#_bookmark35) 41
     13. [The full interaction model with Hostility as the outcome](#_bookmark36) 42
     14. [The full interaction model with Initiation as the outcome](#_bookmark37) 43
     15. [The full interaction model with Intrusiveness as the outcome](#_bookmark38) 44
     16. [The full interaction model with Lead Expert as the outcome](#_bookmark39) 45
     17. [The full interaction model with Lead Patient as the outcome](#_bookmark40) 46
     18. [The full interaction model with Mismatched Affect as the outcome](#_bookmark41) 47
     19. [The full interaction model with Motivation as the outcome](#_bookmark42) 48
     20. [The full interaction model with negative affect as the outcome](#_bookmark43) 49
     21. [The full interaction model with Positive Affect as the outcome](#_bookmark44) 50
     22. [The full interaction model with Persistence as the outcome](#_bookmark45) 51
     23. [The full interaction model with Reciprocity as the outcome](#_bookmark46) 52
     24. [The full interaction model with Silence as the outcome](#_bookmark47) 53
     25. [The full interaction model with Synchrony as the outcome](#_bookmark48) 54
     26. [The full interaction model with Tension as the outcome](#_bookmark49) 55
     27. [The full interaction model with Withdrawal as the outcome](#_bookmark50) 56
  2. [The oxytocin–social-treatment full interaction models: the PANSS scores as the out-](#_bookmark51) [comes](#_bookmark51) 57
     1. [The full interaction model with PANSS total as the outcome](#_bookmark52) 58
     2. [The full interaction model with P (PANSS) as the outcome](#_bookmark53) 59
     3. [The full interaction model with N (PANSS) as the outcome](#_bookmark54) 60
     4. [The full interaction model with G (PANSS) as the outcome](#_bookmark55) 61
     5. [The full interaction model with Withdrawal (PANSS) as the outcome](#_bookmark56) 62
     6. [The full interaction model with Delusion (PANSS) as the outcome](#_bookmark57) 63
     7. [The full interaction model with Anxiety (PANSS) as the outcome](#_bookmark58) 64
     8. [The full interaction model with Disorganization (PANSS) as the outcome](#_bookmark59) 65
     9. [The full interaction model with Poor control (PANSS) as the outcome](#_bookmark60) 66

1. [The oxytocin main effect models](#_bookmark61) 67
   1. [The oxytocin main effect models: the five social interaction factors as the outcomes](#_bookmark62) 67
      1. [The oxytocin main effect model with Factor 1 (synchrony) total as the outcome](#_bookmark63) 68
      2. [The oxytocin main effect model with Factor 2 (initiation) total as the outcome](#_bookmark64) 69
      3. [The oxytocin main effect model with Factor 3 (tension) total as the outcome](#_bookmark65) 70
      4. [The oxytocin main effect model with Factor 4 (withdrawal) total as the outcome](#_bookmark66) 71
      5. [The oxytocin main effect model with Factor 5 (Positive affect) total as the outcome](#_bookmark67) 72
      6. [The oxytocin main effect model with Factor 1 (synchrony) Conflict Interaction as the outcome](#_bookmark68) 73
      7. [The oxytocin main effect model with Factor 2 (initiation) Conflict Interaction as the outcome](#_bookmark69) 74
      8. [The oxytocin main effect model with Factor 3 (tension) Conflict Interaction as the outcome](#_bookmark70) . 75
      9. [The oxytocin main effect model with Factor 4 (withdrawal) Conflict Interaction as the outcome](#_bookmark71) 76
      10. [The oxytocin main effect model with Factor 5 (Positive affect) Conflict Interaction as the](#_bookmark72) [outcome](#_bookmark72) 77
      11. [The oxytocin main effect model with Factor 1 (synchrony) Positive Interaction as the outcome](#_bookmark73) 78
      12. [The oxytocin main effect model with Factor 2 (initiation) Positive Interaction as the outcome](#_bookmark74) 79
      13. [The oxytocin main effect model with Factor 3 (tension) Positive Interaction as the outcome](#_bookmark75) 80
      14. [The oxytocin main effect model with Factor 4 (withdrawal) Positive Interaction as the outcome](#_bookmark76) 81
      15. [The oxytocin main effect model with Factor 5 (Positive affect) Positive Interaction as the](#_bookmark77) [outcome](#_bookmark77) 82
      16. [The oxytocin main effect model with Factor 1 (synchrony) Supportive interaction as the](#_bookmark78) [outcome](#_bookmark78) 83
      17. [The oxytocin main effect model with Factor 2 (initiation) Supportive interaction as the outcome](#_bookmark79) 84
      18. [The oxytocin main effect model with Factor 3 (EmpathEmoEx) Supportive interaction as](#_bookmark80)

[the outcome](#_bookmark80) 85

- - 1. [The oxytocin main effect model with Factor 4 (Positive affect) Supportive interaction as the](#_bookmark81) [outcome](#_bookmark81) 86
    2. [The oxytocin main effect model with Factor 5 (Critisism) Supportive interaction as the outcome](#_bookmark82) 87
    3. [The oxytocin main effect model with CIB total as the outcome](#_bookmark83) 88
  1. [The oxytocin main effect models: the individual social interaction scores as the out-](#_bookmark84) [comes](#_bookmark84) 89
     1. [The oxytocin main effect model with Acknowledgment as the outcome](#_bookmark85) 90
     2. [The oxytocin main effect model with Alert as the outcome](#_bookmark86) 91
     3. [The oxytocin main effect model with Anger as the outcome](#_bookmark87) 92
     4. [The oxytocin main effect model with Anxiety as the outcome](#_bookmark88) 93
     5. [The oxytocin main effect model with Avoidance as the outcome](#_bookmark89) 94
     6. [The oxytocin main effect model with Blatant affect as the outcome](#_bookmark90) 95
     7. [The oxytocin main effect model with Constricted as the outcome](#_bookmark91) 96
     8. [The oxytocin main effect model with Criticism as the outcome](#_bookmark92) 97
     9. [The oxytocin main effect model with Detachment as the outcome](#_bookmark93) 98
     10. [The oxytocin main effect model with Elaboration as the outcome](#_bookmark94) 99
     11. [The oxytocin main effect model with Fluency as the outcome](#_bookmark95) 100
     12. [The oxytocin main effect model with Gaze as the outcome](#_bookmark96) 101
     13. [The oxytocin main effect model with Hostility as the outcome](#_bookmark97) 102
     14. [The oxytocin main effect model with Initiation as the outcome](#_bookmark98) 103
     15. [The oxytocin main effect model with Intrusiveness as the outcome](#_bookmark99) 104
     16. [The oxytocin main effect model with Lead Expert as the outcome](#_bookmark100) 105
     17. [The oxytocin main effect model with Lead Patient as the outcome](#_bookmark101) 106
     18. [The oxytocin main effect model with Mismatched Affect as the outcome](#_bookmark102) 107
     19. [The oxytocin main effect model with Motivation as the outcome](#_bookmark103) 108
     20. [The oxytocin main effect model with Negative affect as the outcome](#_bookmark104) 109
     21. [The oxytocin main effect model with Positive Affect as the outcome](#_bookmark105) 110
     22. [The oxytocin main effect model with Persistence as the outcome](#_bookmark106) 111
     23. [The oxytocin main effect model with Reciprocity as the outcome](#_bookmark107) 112
     24. [The oxytocin main effect model with Silence as the outcome](#_bookmark108) 113
     25. [The oxytocin main effect model with Synchrony as the outcome](#_bookmark109) 114
     26. [The oxytocin main effect model with Tension as the outcome](#_bookmark110) 115
     27. [The oxytocin main effect model with Withdrawal as the outcome](#_bookmark111) 116
  2. [The oxytocin main effect models: the PANSS scores as the outcomes](#_bookmark112) 117
     1. [The oxytocin main effect model with PANSS total as the outcome](#_bookmark113) 118
     2. [The oxytocin main effect model with P (PANSS) as the outcome](#_bookmark114) 119
     3. [The oxytocin main effect model with N (PANSS) as the outcome](#_bookmark115) 120
     4. [The oxytocin main effect model with G (PANSS) as the outcome](#_bookmark116) 121
     5. [The oxytocin main effect model with Withdrawal (PANSS) as the outcome](#_bookmark117) 122
     6. [The oxytocin main effect model with Delusion (PANSS) as the outcome](#_bookmark118) 123
     7. [The oxytocin main effect model with Anxiety (PANSS) as the outcome](#_bookmark119) 124
     8. [The oxytocin main effect model with Disorganization (PANSS) as the outcome](#_bookmark120) 125
     9. [The oxytocin main effect model with Poor control (PANSS) as the outcome](#_bookmark121) 126

1. [The social treatment main effect models](#_bookmark122) 127
   1. [The social treatment main effect models: the five social interaction factors as the](#_bookmark123) [outcomes](#_bookmark123) 127
      1. [The social treatment main effect model with Factor 1 (synchrony) total as the outcome](#_bookmark124) 128
      2. [The social treatment main effect model with Factor 2 (initiation) total as the outcome](#_bookmark125) 129
      3. [The social treatment main effect model with Factor 3 (tension) total as the outcome](#_bookmark126) 130
      4. [The social treatment main effect model with Factor 4 (withdrawal) total as the outcome](#_bookmark127) 131
      5. [The social treatment main effect model with Factor 5 (Positive affect) total as the outcome](#_bookmark128) . 132
      6. [The social treatment main effect model with Factor 1 (synchrony) Conflict Interaction as the](#_bookmark129) [outcome](#_bookmark129) 133
      7. [The social treatment main effect model with Factor 2 (initiation) Conflict Interaction as the](#_bookmark130) [outcome](#_bookmark130) 134
      8. [The social treatment main effect model with Factor 3 (tension) Conflict Interaction as the](#_bookmark131) [outcome](#_bookmark131) 135
      9. [The social treatment main effect model with Factor 4 (withdrawal) Conflict Interaction as the](#_bookmark132) [outcome](#_bookmark132) 136
      10. [The social treatment main effect model with Factor 5 (Positive affect) Conflict Interaction](#_bookmark133)

[as the outcome](#_bookmark133) 137

- - 1. [The social treatment main effect model with Factor 1 (synchrony) Positive Interaction as](#_bookmark134)

[the outcome](#_bookmark134) 138

- - 1. [The social treatment main effect model with Factor 2 (initiation) Positive Interaction as the](#_bookmark135) [outcome](#_bookmark135) 139
    2. [The social treatment main effect model with Factor 3 (tension) Positive Interaction as the](#_bookmark136) [outcome](#_bookmark136) 140
    3. [The social treatment main effect model with Factor 4 (withdrawal) Positive Interaction as](#_bookmark137)

[the outcome](#_bookmark137) 141

- - 1. [The social treatment main effect model with Factor 5 (Positive affect) Positive Interaction](#_bookmark138)

[as the outcome](#_bookmark138) 142

- - 1. [The social treatment main effect model with Factor 1 (synchrony) Supportive interaction as](#_bookmark139)

[the outcome](#_bookmark139) 143

- - 1. [The social treatment main effect model with Factor 2 (initiation) Supportive interaction as](#_bookmark140)

[the outcome](#_bookmark140) 144

- - 1. [The social treatment main effect model with Factor 3 (EmpathEmoEx) Supportive interaction](#_bookmark141)

[as the outcome](#_bookmark141) 145

- - 1. [The social treatment main effect model with Factor 4 (Positive affect) Supportive interaction](#_bookmark142)

[as the outcome](#_bookmark142) 146

- - 1. [The social treatment main effect model with Factor 5 (Critisism) Supportive interaction as](#_bookmark143)

[the outcome](#_bookmark143) 147

- - 1. [The social treatment main effect model with CIB total as the outcome](#_bookmark144) 148
  1. [The social treatment main effect models: the individual social interaction scores as the](#_bookmark145) [outcomes](#_bookmark145) 149
     1. [The social treatment main effect model with Acknowledgment as the outcome](#_bookmark146) 150
     2. [The social treatment main effect model with Alert as the outcome](#_bookmark147) 151
     3. [The social treatment main effect model with Anger as the outcome](#_bookmark148) 152
     4. [The social treatment main effect model with Anxiety as the outcome](#_bookmark149) 153
     5. [The social treatment main effect model with Avoidance as the outcome](#_bookmark150) 154
     6. [The social treatment main effect model with Blatant affect as the outcome](#_bookmark151) 155
     7. [The social treatment main effect model with Constricted as the outcome](#_bookmark152) 156
     8. [The social treatment main effect model with Criticism as the outcome](#_bookmark153) 157
     9. [The social treatment main effect model with Detachment as the outcome](#_bookmark154) 158
     10. [The social treatment main effect model with Elaboration as the outcome](#_bookmark155) 159
     11. [The social treatment main effect model with Fluency as the outcome](#_bookmark156) 160
     12. [The social treatment main effect model with Gaze as the outcome](#_bookmark157) 161
     13. [The social treatment main effect model with Hostility as the outcome](#_bookmark158) 162
     14. [The social treatment main effect model with Initiation as the outcome](#_bookmark159) 163
     15. [The social treatment main effect model with Intrusiveness as the outcome](#_bookmark160) 164
     16. [The social treatment main effect model with Lead Expert as the outcome](#_bookmark161) 165
     17. [The social treatment main effect model with Lead Patient as the outcome](#_bookmark162) 166
     18. [The social treatment main effect model with Mismatched Affect as the outcome](#_bookmark163) 167
     19. [The social treatment main effect model with Motivation as the outcome](#_bookmark164) 168
     20. [The social treatment main effect model with Negative affect as the outcome](#_bookmark165) 169
     21. [The social treatment main effect model with Positive Affect as the outcome](#_bookmark166) 170
     22. [The social treatment main effect model with Persistence as the outcome](#_bookmark167) 171
     23. [The social treatment main effect model with Reciprocity as the outcome](#_bookmark168) 172
     24. [The social treatment main effect model with Silence as the outcome](#_bookmark169) 173
     25. [The social treatment main effect model with Synchrony as the outcome](#_bookmark170) 174
     26. [The social treatment main effect model with Tension as the outcome](#_bookmark171) 175
     27. [The social treatment main effect model with Withdrawal as the outcome](#_bookmark172) 176
  2. [The social treatment main effect models: the PANSS scores as the outcomes](#_bookmark173) 177
     1. [The social treatment main effect model with PANSS total as the outcome](#_bookmark174) 178
     2. [The social treatment main effect model with P (PANSS) as the outcome](#_bookmark175) 179
     3. [The social treatment main effect model with N (PANSS) as the outcome](#_bookmark176) 180
     4. [The social treatment main effect model with G (PANSS) as the outcome](#_bookmark177) 181
     5. [The social treatment main effect model with Withdrawal (PANSS) as the outcome](#_bookmark178) 182
     6. [The social treatment main effect model with Delusion (PANSS) as the outcome](#_bookmark179) 183
     7. [The social treatment main effect model with Anxiety (PANSS) as the outcome](#_bookmark180) 184
     8. [The social treatment main effect model with Disorganization (PANSS) as the outcome](#_bookmark181) 185
     9. [The social treatment main effect model with Poor control (PANSS) as the outcome](#_bookmark182) 186

1. **Antipsychotics use 187**

# The oxytocin–social-treatment full interaction models

To estimate the effects over t ime of oxytocin, socical t reatment, a nd t heir interaction, m ixed effects models were used for each of the five s ocial i nteraction f actors, t he i ndividual s ocial i nteraction s cores, a nd the PANSS scores as the outcome. In these models, multiple measurements (one per time point) for each patient were modeled as random effects. The coefficients of interest are the cha nge in the effects of the intervention variables (the combination of oxytocin and social treatment) over time, which are represented as the time interactions. I.e., oxytocin × time, social treatment × time, and oxytocin × social treatment × time. The p- values due to multiple testing were adjusted using Bonferroni corrrection for each group of outcomes (the set of social interaction factors, the set of the individual social interaction scores, and the set of PANSS-related scores) and the corrected values are referred to as the ‘q-values.’

# The oxytocin–social-treatment full interaction models: the five social interaction factors as the outcomes

This section provides the results for each of the five social interaction factors—initiation, synchrony, positive affects, tension, a nd withdrawal—as t he o utcome in t he models.

## The full interaction model with Factor 1 (synchrony) total as the outcome

Table 1.1.1. The full interaction model with Factor 1 (synchrony) total as the outcome, N = 43

supportive psychotherapy

|  | Estimate | Std. Error | df | t value | p-value | q-value |
| --- | --- | --- | --- | --- | --- | --- |
| (Intercept) | 0.05 | 0.29 | 57.66 | 0.16 | 0.87 | 1 |
| oxytocin | -0.15 | 0.45 | 57.66 | -0.33 | 0.74 | 1 |
| social treatment | -0.22 | 0.43 | 57.66 | -0.53 | 0.60 | 1 |
| time | 0.01 | 0.09 | 39.00 | 0.12 | 0.91 | 1 |
| oxytocin×social treatment | 0.16 | 0.64 | 57.66 | 0.24 | 0.81 | 1 |
| oxytocin×time | 0.11 | 0.14 | 39.00 | 0.84 | 0.41 | 1 |
| social treatment×time | 0.09 | 0.13 | 39.00 | 0.70 | 0.49 | 1 |
| oxytocin×social treatment×time | -0.15 | 0.19 | 39.00 | -0.79 | 0.43 | 1 |

social skills training

1.0

0.5


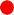

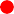

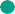

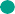

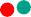

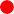

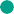


Factor 1 (synchrony) total

0.0

oxytocin


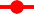

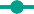
placebo oxytocin

−0.5

0 1 2 3 0 1 2 3

time

Figure 1.1.1 The predicted values of the full interaction model with Factor 1 (synchrony) total as the outcome

## The full interaction model with Factor 2 (initiation) total as the outcome

Table 1.1.2. The full interaction model with Factor 2 (initiation) total as the outcome, N = 43

|  | Estimate | Std. Error | df | t value | p-value | q-value |
| --- | --- | --- | --- | --- | --- | --- |
| (Intercept) | 0.10 | 0.29 | 53.39 | 0.34 | 0.73 | 1 |
| oxytocin | -0.22 | 0.45 | 53.39 | -0.48 | 0.63 | 1 |
| social treatment | 0.01 | 0.43 | 53.39 | 0.03 | 0.98 | 1 |
| time | -0.06 | 0.08 | 39.00 | -0.79 | 0.44 | 1 |
| oxytocin×social treatment | -0.10 | 0.64 | 53.39 | -0.16 | 0.88 | 1 |
| oxytocin×time | 0.14 | 0.12 | 39.00 | 1.17 | 0.25 | 1 |
| social treatment×time | 0.04 | 0.11 | 39.00 | 0.39 | 0.70 | 1 |
| oxytocin×social treatment×time | -0.02 | 0.17 | 39.00 | -0.12 | 0.90 | 1 |

social skills training

0.5


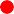

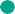

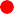

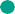

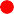

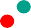

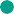


supportive psychotherapy

0.0

Factor 2 (initiation) total

oxytocin


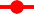

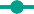
placebo oxytocin

−0.5

0 1 2 3 0 1 2 3

time

Figure 1.1.2 The predicted values of the full interaction model with Factor 2 (initiation) total as the outcome

## The full interaction model with Factor 3 (tension) total as the outcome

Table 1.1.3. The full interaction model with Factor 3 (tension) total as the outcome, N = 43

|  | Estimate | Std. Error | df | t value | p-value | q-value |
| --- | --- | --- | --- | --- | --- | --- |
| (Intercept) | -0.51 | 0.27 | 60.91 | -1.91 | 0.06 | 1.00 |
| oxytocin | 0.95 | 0.42 | 60.91 | 2.30 | 0.03 | 0.53 |
| social treatment | 1.02 | 0.39 | 60.91 | 2.61 | 0.01 | 0.24 |
| time | 0.06 | 0.09 | 39.00 | 0.75 | 0.46 | 1.00 |
| oxytocin×social treatment | -1.19 | 0.59 | 60.91 | -2.02 | 0.05 | 1.00 |
| oxytocin×time | -0.21 | 0.13 | 39.00 | -1.59 | 0.12 | 1.00 |
| social treatment×time | -0.15 | 0.13 | 39.00 | -1.22 | 0.23 | 1.00 |
| oxytocin×social treatment×time | 0.09 | 0.19 | 39.00 | 0.48 | 0.63 | 1.00 |

social skills training

1.0


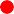

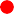

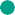

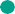

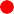

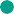

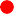

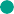


supportive psychotherapy

0.5

Factor 3 (tension) total

0.0

oxytocin


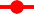

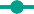
placebo oxytocin

−0.5

−1.0

0 1 2 3 0 1 2 3

time

Figure 1.1.3 The predicted values of the full interaction model with Factor 3 (tension) total as the outcome

## The full interaction model with Factor 4 (withdrawal) total as the outcome

Table 1.1.4. The full interaction model with Factor 4 (withdrawal) total as the outcome, N = 43


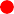

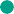

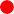

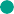


supportive psychotherapy

|  | Estimate | Std. Error | df | t value | p-value | q-value |
| --- | --- | --- | --- | --- | --- | --- |
| (Intercept) | -0.20 | 0.28 | 61.51 | -0.70 | 0.48 | 1 |
| oxytocin | -0.12 | 0.43 | 61.51 | -0.29 | 0.78 | 1 |
| social treatment | 0.64 | 0.41 | 61.51 | 1.57 | 0.12 | 1 |
| time | 0.01 | 0.09 | 39.00 | 0.08 | 0.94 | 1 |
| oxytocin×social treatment | -0.21 | 0.62 | 61.51 | -0.35 | 0.73 | 1 |
| oxytocin×time | 0.01 | 0.14 | 39.00 | 0.07 | 0.95 | 1 |
| social treatment×time | -0.13 | 0.13 | 39.00 | -0.95 | 0.35 | 1 |
| oxytocin×social treatment×time | 0.08 | 0.20 | 39.00 | 0.40 | 0.69 | 1 |

social skills training

1.0

0.5

Factor 4 (withdrawal) total

0.0

oxytocin


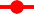

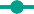
placebo oxytocin

−0.5


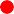

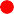

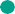

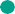


−1.0

0 1 2 3

time

0 1 2 3

Figure 1.1.4 The predicted values of the full interaction model with Factor 4 (withdrawal) total as the outcome

## The full interaction model with Factor 5 (Positive affect) total as the out- come

Table 1.1.5. The full interaction model with Factor 5 (Positive affect) total as the outcome, N = 43

supportive psychotherapy

|  | Estimate | Std. Error | df | t value | p-value | q-value |
| --- | --- | --- | --- | --- | --- | --- |
| (Intercept) | 0.40 | 0.26 | 53.04 | 1.52 | 0.14 | 1.00 |
| oxytocin | -0.96 | 0.41 | 53.04 | -2.34 | 0.02 | 0.48 |
| social treatment | -0.74 | 0.39 | 53.04 | -1.93 | 0.06 | 1.00 |
| time | -0.07 | 0.07 | 39.00 | -0.97 | 0.34 | 1.00 |
| oxytocin×social treatment | 1.03 | 0.58 | 53.04 | 1.77 | 0.08 | 1.00 |
| oxytocin×time | 0.28 | 0.11 | 39.00 | 2.63 | 0.01 | 0.26 |
| social treatment×time | 0.19 | 0.10 | 39.00 | 1.89 | 0.07 | 1.00 |
| oxytocin×social treatment×time | -0.36 | 0.15 | 39.00 | -2.38 | 0.02 | 0.47 |

social skills training

1.0

0.5


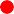

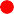

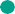

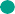

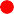

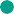

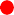

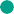


Factor 5 (Positive affect) total

0.0

oxytocin


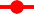

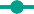
placebo oxytocin

−0.5

−1.0

0 1 2 3 0 1 2 3

time

Figure 1.1.5 The predicted values of the full interaction model with Factor 5 (Positive affect) total as the outcome

## The full interaction model with Factor 1 (synchrony) Conflict Interaction as the outcome

Table 1.1.6. The full interaction model with Factor 1 (synchrony) Conflict Interaction as the outcome, N = 43

supportive psychotherapy

|  | Estimate | Std. Error | df | t value | p-value | q-value |
| --- | --- | --- | --- | --- | --- | --- |
| (Intercept) | -0.15 | 0.28 | 61.15 | -0.54 | 0.59 | 1 |
| oxytocin | 0.40 | 0.44 | 61.15 | 0.90 | 0.37 | 1 |
| social treatment | 0.16 | 0.42 | 61.15 | 0.39 | 0.70 | 1 |
| time | 0.02 | 0.09 | 39.00 | 0.20 | 0.84 | 1 |
| oxytocin×social treatment | -0.94 | 0.62 | 61.15 | -1.50 | 0.14 | 1 |
| oxytocin×time | -0.02 | 0.14 | 39.00 | -0.11 | 0.92 | 1 |
| social treatment×time | 0.05 | 0.14 | 39.00 | 0.38 | 0.71 | 1 |
| oxytocin×social treatment×time | 0.12 | 0.20 | 39.00 | 0.58 | 0.56 | 1 |

social skills training

1.0

0.5


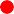

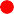

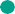

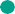

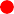

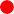

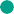

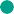


Factor 1 (synchrony) Conflict Interaction

0.0

oxytocin


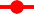

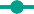
placebo oxytocin

−0.5

−1.0

0 1 2 3 0 1 2 3

time

Figure 1.1.6 The predicted values of the full interaction model with CIB total as the outcome

## The full interaction model with Factor 2 (initiation) Conflict Interaction as the outcome

Table 1.1.7. The full interaction model with Factor 2 (initiation) Conflict Interaction as the outcome, N = 43

|  | Estimate | Std. Error | df | t value | p-value | q-value |
| --- | --- | --- | --- | --- | --- | --- |
| (Intercept) | -0.03 | 0.29 | 58.91 | -0.10 | 0.92 | 1 |
| oxytocin | -0.06 | 0.45 | 58.91 | -0.14 | 0.89 | 1 |
| social treatment | 0.10 | 0.42 | 58.91 | 0.23 | 0.82 | 1 |
| time | -0.03 | 0.09 | 39.00 | -0.34 | 0.73 | 1 |
| oxytocin×social treatment | -0.31 | 0.63 | 58.91 | -0.49 | 0.63 | 1 |
| oxytocin×time | 0.19 | 0.14 | 39.00 | 1.38 | 0.18 | 1 |
| social treatment×time | 0.01 | 0.13 | 39.00 | 0.11 | 0.92 | 1 |
| oxytocin×social treatment×time | -0.09 | 0.20 | 39.00 | -0.48 | 0.63 | 1 |

supportive psychotherapy

social skills training

1.0


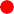

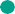

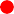

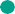


0.5


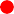

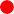

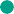

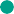


Factor 2 (initiation) Conflict Interaction

0.0

oxytocin


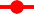

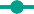
placebo oxytocin

−0.5

−1.0

0 1 2 3

time

0 1 2 3

Figure 1.1.7 The predicted values of the full interaction model with Factor 1 (synchrony) Conflict Interaction as the outcome

## The full interaction model with Factor 3 (tension) Conflict Interaction as the outcome

Table 1.1.8. The full interaction model with Factor 3 (tension) Conflict Interaction as the outcome, N = 43

|  | Estimate | Std. Error | df | t value | p-value | q-value |
| --- | --- | --- | --- | --- | --- | --- |
| (Intercept) | -0.33 | 0.28 | 68.96 | -1.18 | 0.24 | 1.00 |
| oxytocin | 0.54 | 0.44 | 68.96 | 1.23 | 0.22 | 1.00 |
| social treatment | 0.85 | 0.41 | 68.96 | 2.05 | 0.04 | 0.93 |
| time | 0.05 | 0.11 | 39.00 | 0.45 | 0.66 | 1.00 |
| oxytocin×social treatment | -0.83 | 0.62 | 68.96 | -1.34 | 0.19 | 1.00 |
| oxytocin×time | -0.15 | 0.16 | 39.00 | -0.93 | 0.36 | 1.00 |
| social treatment×time | -0.16 | 0.16 | 39.00 | -1.05 | 0.30 | 1.00 |
| oxytocin×social treatment×time | 0.12 | 0.23 | 39.00 | 0.50 | 0.62 | 1.00 |

social skills training

1.0


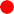

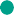

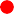

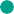


supportive psychotherapy

0.5


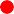

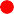

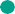

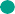


Factor 3 (tension) Conflict Interaction

0.0

oxytocin


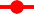

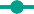
placebo oxytocin

−0.5

0 1 2 3 0 1 2 3

time

Figure 1.1.8 The predicted values of the full interaction model with Factor 2 (initiation) Conflict Interaction as the outcome

## The full interaction model with Factor 4 (withdrawal) Conflict Interaction as the outcome

Table 1.1.9. The full interaction model with Factor 4 (withdrawal) Conflict Interaction as the outcome, N

= 43

|  | Estimate | Std. Error | df | t value | p-value | q-value |
| --- | --- | --- | --- | --- | --- | --- |
| (Intercept) | -0.50 | 0.28 | 59.37 | -1.81 | 0.07 | 1.00 |
| oxytocin | 0.41 | 0.43 | 59.37 | 0.96 | 0.34 | 1.00 |
| social treatment | 1.11 | 0.41 | 59.37 | 2.73 | 0.01 | 0.17 |
| time | 0.13 | 0.09 | 39.00 | 1.50 | 0.14 | 1.00 |
| oxytocin×social treatment | -1.01 | 0.61 | 59.37 | -1.65 | 0.10 | 1.00 |
| oxytocin×time | -0.16 | 0.13 | 39.00 | -1.17 | 0.25 | 1.00 |
| social treatment×time | -0.33 | 0.13 | 39.00 | -2.57 | 0.01 | 0.29 |
| oxytocin×social treatment×time | 0.39 | 0.19 | 39.00 | 2.05 | 0.05 | 0.99 |

social skills training

1.0


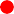

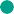

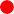

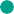


supportive psychotherapy

Factor 4 (withdrawal) Conflict Interaction

0.5


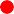

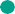

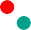


0.0

oxytocin


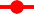

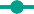
placebo oxytocin

−0.5

−1.0

0 1 2 3 0 1 2 3

time

Figure 1.1.9 The predicted values of the full interaction model with Factor 3 (tension) Conflict Interaction as the outcome

## The full interaction model with Factor 5 (Positive affect) Conflict Inter- action as the outcome

Table 1.1.10. The full interaction model with Factor 5 (Positive affect) Conflict Interaction as the outcome, N = 43

supportive psychotherapy

|  | Estimate | Std. Error | df | t value | p-value | q-value |
| --- | --- | --- | --- | --- | --- | --- |
| (Intercept) | 0.22 | 0.26 | 52.68 | 0.85 | 0.40 | 1.00 |
| oxytocin | -0.44 | 0.41 | 52.68 | -1.07 | 0.29 | 1.00 |
| social treatment | -0.70 | 0.39 | 52.68 | -1.81 | 0.08 | 1.00 |
| time | 0.03 | 0.07 | 39.00 | 0.50 | 0.62 | 1.00 |
| oxytocin×social treatment | 0.56 | 0.58 | 52.68 | 0.96 | 0.34 | 1.00 |
| oxytocin×time | 0.14 | 0.11 | 39.00 | 1.28 | 0.21 | 1.00 |
| social treatment×time | 0.21 | 0.10 | 39.00 | 2.09 | 0.04 | 0.91 |
| oxytocin×social treatment×time | -0.45 | 0.15 | 39.00 | -2.94 | 0.01 | 0.11 |

social skills training

1.0

0.5


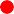

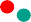

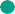

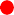

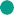

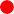

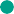


Factor 5 (Positive affect) Conflict Interaction

0.0

oxytocin


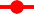

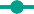
placebo oxytocin

−0.5

−1.0

0 1 2 3 0 1 2 3

time

Figure 1.1.10 The predicted values of the full interaction model with Factor 4 (withdrawal) Conflict Inter- action as the outcome

## The full interaction model with Factor 1 (synchrony) Positive Interaction as the outcome

Table 1.1.11. The full interaction model with Factor 1 (synchrony) Positive Interaction as the outcome, N

= 43

|  | Estimate | Std. Error | df | t value | p-value | q-value |
| --- | --- | --- | --- | --- | --- | --- |
| (Intercept) | -0.05 | 0.28 | 69.6 | -0.19 | 0.85 | 1 |
| oxytocin | -0.16 | 0.44 | 69.6 | -0.37 | 0.72 | 1 |
| social treatment | 0.06 | 0.41 | 69.6 | 0.14 | 0.89 | 1 |
| time | 0.12 | 0.11 | 39.0 | 1.14 | 0.26 | 1 |
| oxytocin×social treatment | 0.02 | 0.62 | 69.6 | 0.04 | 0.97 | 1 |
| oxytocin×time | 0.09 | 0.17 | 39.0 | 0.55 | 0.59 | 1 |
| social treatment×time | -0.10 | 0.16 | 39.0 | -0.62 | 0.54 | 1 |
| oxytocin×social treatment×time | -0.15 | 0.24 | 39.0 | -0.64 | 0.53 | 1 |

social skills training

1.0


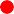

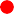

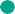

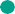


supportive psychotherapy

0.5

Factor 1 (synchrony) Positive Interaction

0.0

oxytocin

placebo oxytocin

−0.5

0 1 2 3 0 1 2 3

time

Figure 1.1.11 The predicted values of the full interaction model with Factor 5 (Positive affect) Conflict Interaction as the outcome

## The full interaction model with Factor 2 (initiation) Positive Interaction as the outcome

Table 1.1.12. The full interaction model with Factor 2 (initiation) Positive Interaction as the outcome, N = 43

|  | Estimate | Std. Error | df | t value | p-value | q-value |
| --- | --- | --- | --- | --- | --- | --- |
| (Intercept) | 0.10 | 0.28 | 61.89 | 0.34 | 0.73 | 1 |
| oxytocin | 0.02 | 0.44 | 61.89 | 0.05 | 0.96 | 1 |
| social treatment | -0.12 | 0.42 | 61.89 | -0.29 | 0.77 | 1 |
| time | -0.08 | 0.09 | 39.00 | -0.89 | 0.38 | 1 |
| oxytocin×social treatment | -0.19 | 0.63 | 61.89 | -0.30 | 0.77 | 1 |
| oxytocin×time | 0.01 | 0.15 | 39.00 | 0.05 | 0.96 | 1 |
| social treatment×time | 0.16 | 0.14 | 39.00 | 1.18 | 0.25 | 1 |
| oxytocin×social treatment×time | 0.07 | 0.21 | 39.00 | 0.32 | 0.75 | 1 |

1.0

supportive psychotherapy

social skills training

0.5

Factor 2 (initiation) Positive Interaction

0.0

oxytocin

placebo oxytocin

−0.5

0 1 2 3 0 1 2 3

time

Figure 1.1.12 The predicted values of the full interaction model with Factor 1 (synchrony) Positive Interaction as the outcome

## The full interaction model with Factor 3 (tension) Positive Interaction as the outcome

Table 1.1.13. The full interaction model with Factor 3 (tension) Positive Interaction as the outcome, N = 43

supportive psychotherapy

|  | Estimate | Std. Error | df | t value | p-value | q-value |
| --- | --- | --- | --- | --- | --- | --- |
| (Intercept) | -0.50 | 0.27 | 64.83 | -1.83 | 0.07 | 1.00 |
| oxytocin | 0.78 | 0.43 | 64.83 | 1.83 | 0.07 | 1.00 |
| social treatment | 0.90 | 0.40 | 64.83 | 2.22 | 0.03 | 0.62 |
| time | 0.08 | 0.10 | 39.00 | 0.83 | 0.41 | 1.00 |
| oxytocin×social treatment | -0.92 | 0.60 | 64.83 | -1.52 | 0.13 | 1.00 |
| oxytocin×time | -0.14 | 0.15 | 39.00 | -0.92 | 0.37 | 1.00 |
| social treatment×time | -0.14 | 0.14 | 39.00 | -0.97 | 0.34 | 1.00 |
| oxytocin×social treatment×time | 0.04 | 0.21 | 39.00 | 0.19 | 0.85 | 1.00 |

social skills training

1.0

0.5

Factor 3 (tension) Positive Interaction

0.0

oxytocin

placebo oxytocin

−0.5

−1.0

0 1 2 3 0 1 2 3

time

Figure 1.1.13 The predicted values of the full interaction model with Factor 2 (initiation) Positive Interaction as the outcome

## The full interaction model with Factor 4 (withdrawal) Positive Interaction as the outcome

Table 1.1.14. The full interaction model with Factor 4 (withdrawal) Positive Interaction as the outcome, N

= 43

|  | Estimate | Std. Error | df | t value | p-value | q-value |
| --- | --- | --- | --- | --- | --- | --- |
| (Intercept) | -0.11 | 0.28 | 75.47 | -0.40 | 0.69 | 1 |
| oxytocin | -0.32 | 0.44 | 75.47 | -0.73 | 0.47 | 1 |
| social treatment | 0.30 | 0.41 | 75.47 | 0.72 | 0.47 | 1 |
| time | 0.04 | 0.12 | 39.00 | 0.32 | 0.75 | 1 |
| oxytocin×social treatment | 0.08 | 0.62 | 75.47 | 0.13 | 0.90 | 1 |
| oxytocin×time | 0.14 | 0.19 | 39.00 | 0.74 | 0.46 | 1 |
| social treatment×time | -0.06 | 0.18 | 39.00 | -0.34 | 0.74 | 1 |
| oxytocin×social treatment×time | -0.15 | 0.27 | 39.00 | -0.56 | 0.58 | 1 |

social skills training

0.5

supportive psychotherapy

Factor 4 (withdrawal) Positive Interaction

0.0 oxytocin

placebo oxytocin

−0.5

−1.0

0 1 2 3 0 1 2 3

time

Figure 1.1.14 The predicted values of the full interaction model with Factor 3 (tension) Positive Interaction as the outcome

## The full interaction model with Factor 5 (Positive affect) Positive Inter- action as the outcome

Table 1.1.15. The full interaction model with Factor 5 (Positive affect) Positive Interaction as the outcome, N = 43

|  | Estimate | Std. Error | df | t value | p-value | q-value |
| --- | --- | --- | --- | --- | --- | --- |
| (Intercept) | 0.36 | 0.26 | 60.26 | 1.35 | 0.18 | 1.00 |
| oxytocin | -0.87 | 0.41 | 60.26 | -2.10 | 0.04 | 0.85 |
| social treatment | -0.37 | 0.39 | 60.26 | -0.94 | 0.35 | 1.00 |
| time | -0.09 | 0.08 | 39.00 | -1.03 | 0.31 | 1.00 |
| oxytocin×social treatment | 0.68 | 0.59 | 60.26 | 1.16 | 0.25 | 1.00 |
| oxytocin×time | 0.29 | 0.13 | 39.00 | 2.22 | 0.03 | 0.68 |
| social treatment×time | 0.07 | 0.12 | 39.00 | 0.59 | 0.56 | 1.00 |
| oxytocin×social treatment×time | -0.32 | 0.19 | 39.00 | -1.70 | 0.10 | 1.00 |

social skills training

0.5

supportive psychotherapy

Factor 5 (Positive affect) Positive Interaction

0.0

oxytocin

placebo oxytocin

−0.5

−1.0

0 1 2 3 0 1 2 3

time

Figure 1.1.15 The predicted values of the full interaction model with Factor 4 (withdrawal) Positive Inter- action as the outcome

## 1.1.16 The full interaction model with Factor 1 (synchrony) Supportive

**interaction as the outcome**

Table 1.1.16. The full interaction model with Factor 1 (synchrony) Supportive interaction as the outcome, N = 43

|  | Estimate | Std. Error | df | t value | p-value | q-value |
| --- | --- | --- | --- | --- | --- | --- |
| (Intercept) | 0.62 | 0.28 | 73.53 | 2.24 | 0.03 | 0.59 |
| oxytocin | -1.03 | 0.43 | 73.53 | -2.38 | 0.02 | 0.42 |
| social treatment | -0.96 | 0.41 | 73.53 | -2.34 | 0.02 | 0.46 |
| time | -0.18 | 0.11 | 39.00 | -1.57 | 0.12 | 1.00 |
| oxytocin×social treatment | 1.29 | 0.62 | 73.53 | 2.09 | 0.04 | 0.84 |
| oxytocin×time | 0.36 | 0.18 | 39.00 | 2.01 | 0.05 | 1.00 |
| social treatment×time | 0.22 | 0.17 | 39.00 | 1.34 | 0.19 | 1.00 |
| oxytocin×social treatment×time | -0.40 | 0.25 | 39.00 | -1.57 | 0.13 | 1.00 |

social skills training

1.0

supportive psychotherapy

Factor 1 (synchrony) Supportive interaction

0.5

0.0

oxytocin

placebo oxytocin

−0.5

−1.0

0 1 2 3 0 1 2 3

time

Figure 1.1.16 The predicted values of the full interaction model with Factor 5 (Positive affect) Positive Interaction as the outcome

## 1.1.17 The full interaction model with Factor 2 (initiation) Supportive interaction

**as the outcome**

Table 1.1.17. The full interaction model with Factor 2 (initiation) Supportive interaction as the outcome, N

= 43

|  | Estimate | Std. Error | df | t value | p-value | q-value |
| --- | --- | --- | --- | --- | --- | --- |
| (Intercept) | 0.56 | 0.28 | 58.63 | 2.01 | 0.05 | 1 |
| oxytocin | -0.52 | 0.43 | 58.63 | -1.20 | 0.24 | 1 |
| social treatment | -0.73 | 0.41 | 58.63 | -1.77 | 0.08 | 1 |
| time | -0.13 | 0.09 | 39.00 | -1.50 | 0.14 | 1 |
| oxytocin×social treatment | 0.57 | 0.62 | 58.63 | 0.92 | 0.36 | 1 |
| oxytocin×time | 0.13 | 0.13 | 39.00 | 0.95 | 0.35 | 1 |
| social treatment×time | 0.08 | 0.13 | 39.00 | 0.66 | 0.51 | 1 |
| oxytocin×social treatment×time | -0.09 | 0.19 | 39.00 | -0.47 | 0.64 | 1 |

supportive psychotherapy

social skills training

1.0

0.5

Factor 2 (initiation) Supportive interaction

0.0

oxytocin

placebo oxytocin

−0.5

−1.0

0 1 2 3

time

0 1 2 3

Figure 1.1.17 The predicted values of the full interaction model with Factor 1 (synchrony) Supportive

interaction as the outcome

## The full interaction model with Factor 3 (EmpathEmoEx) Supportive

**interaction as the outcome**

Table 1.1.18. The full interaction model with Factor 3 (EmpathEmoEx) Supportive interaction as the out- come, N = 43

|  | Estimate | Std. Error | df | t value | p-value | q-value |
| --- | --- | --- | --- | --- | --- | --- |
| (Intercept) | 0.21 | 0.28 | 78 | 0.74 | 0.46 | 1 |
| oxytocin | 0.36 | 0.44 | 78 | 0.81 | 0.42 | 1 |
| social treatment | 0.03 | 0.42 | 78 | 0.08 | 0.94 | 1 |
| time | -0.16 | 0.13 | 78 | -1.19 | 0.24 | 1 |
| oxytocin×social treatment | -0.68 | 0.62 | 78 | -1.09 | 0.28 | 1 |
| oxytocin×time | -0.11 | 0.21 | 78 | -0.52 | 0.61 | 1 |
| social treatment×time | 0.05 | 0.20 | 78 | 0.25 | 0.80 | 1 |
| oxytocin×social treatment×time | 0.14 | 0.29 | 78 | 0.48 | 0.63 | 1 |

supportive psychotherapy

social skills training

1.0

Factor 3 (EmpathEmoEx) Supportive interaction

0.5

oxytocin

placebo

0.0 oxytocin

−0.5

−1.0

0 1 2 3

time

0 1 2 3

Figure 1.1.18 The predicted values of the full interaction model with Factor 2 (initiation) Supportive

interaction as the outcome

## The full interaction model with Factor 4 (Positive affect) Supportive

**interaction as the outcome**

Table 1.1.19. The full interaction model with Factor 4 (Positive affect) Supportive interaction as the outcome, N = 43

supportive psychotherapy

Factor 4 (Positive affect) Supportive interaction

|  | Estimate | Std. Error | df | t value | p-value | q-value |
| --- | --- | --- | --- | --- | --- | --- |
| (Intercept) | 0.18 | 0.26 | 67.73 | 0.67 | 0.51 | 1 |
| oxytocin | -0.71 | 0.41 | 67.73 | -1.74 | 0.09 | 1 |
| social treatment | -0.32 | 0.39 | 67.73 | -0.83 | 0.41 | 1 |
| time | 0.12 | 0.10 | 39.00 | 1.27 | 0.21 | 1 |
| oxytocin×social treatment | 0.58 | 0.58 | 67.73 | 0.99 | 0.32 | 1 |
| oxytocin×time | 0.08 | 0.15 | 39.00 | 0.53 | 0.60 | 1 |
| social treatment×time | -0.10 | 0.14 | 39.00 | -0.71 | 0.48 | 1 |
| oxytocin×social treatment×time | -0.13 | 0.21 | 39.00 | -0.61 | 0.55 | 1 |

social skills training

1.0

0.5

0.0

oxytocin

placebo oxytocin

−0.5

−1.0

0 1 2 3 0 1 2 3

time

Figure 1.1.19 The predicted values of the full interaction model with Factor 3 (EmpathEmoEx) Supportive

interaction as the outcome

## 1.1.20 The full interaction model with Factor 5 (Critisism) Supportive interaction

**as the outcome**

Table 1.1.20. The full interaction model with Factor 5 (Critisism) Supportive interaction as the outcome, N

= 43

|  | Estimate | Std. Error | df | t value | p-value | q-value |
| --- | --- | --- | --- | --- | --- | --- |
| (Intercept) | -0.36 | 0.27 | 76.66 | -1.33 | 0.19 | 1.00 |
| oxytocin | 0.91 | 0.43 | 76.66 | 2.12 | 0.04 | 0.79 |
| social treatment | -0.05 | 0.41 | 76.66 | -0.13 | 0.90 | 1.00 |
| time | 0.19 | 0.12 | 39.00 | 1.61 | 0.11 | 1.00 |
| oxytocin×social treatment | -0.18 | 0.61 | 76.66 | -0.29 | 0.77 | 1.00 |
| oxytocin×time | -0.26 | 0.19 | 39.00 | -1.39 | 0.17 | 1.00 |
| social treatment×time | -0.12 | 0.18 | 39.00 | -0.69 | 0.49 | 1.00 |
| oxytocin×social treatment×time | 0.06 | 0.27 | 39.00 | 0.21 | 0.83 | 1.00 |

social skills training

1.0

supportive psychotherapy

Factor 5 (Critisism) Supportive interaction

0.5

0.0

oxytocin

placebo oxytocin

−0.5

−1.0

0 1 2 3 0 1 2 3

time

Figure 1.1.20 The predicted values of the full interaction model with Factor 4 (Positive affect) Supportive

interaction as the outcome

## 1.1.21 The full interaction model with CIB total as the outcome

Table 1.1.21. The full interaction model with CIB total as the outcome, N = 43

|  | Estimate | Std. Error | df | t value | p-value | q-value |
| --- | --- | --- | --- | --- | --- | --- |
| (Intercept) | 22.01 | 2.62 | 50.07 | 8.41 | 0.00 | 0 |
| oxytocin | -4.99 | 4.09 | 50.07 | -1.22 | 0.23 | 1 |
| social treatment | -3.82 | 3.87 | 50.07 | -0.99 | 0.33 | 1 |
| time | -0.24 | 0.62 | 39.00 | -0.38 | 0.71 | 1 |
| oxytocin×social treatment | 6.00 | 5.81 | 50.07 | 1.03 | 0.31 | 1 |
| oxytocin×time | 1.11 | 0.97 | 39.00 | 1.14 | 0.26 | 1 |
| social treatment×time | 0.92 | 0.92 | 39.00 | 1.00 | 0.32 | 1 |
| oxytocin×social treatment×time | -1.11 | 1.38 | 39.00 | -0.80 | 0.43 | 1 |

social skills training

25

supportive psychotherapy

20 oxytocin

CIB total

placebo oxytocin

15

10

0 1 2 3

time

0 1 2 3

Figure 1.1.21 The predicted values of the full interaction model with Factor 5 (Critisism) Supportive

interaction as the outcome

# The oxytocin–social-treatment full interaction models: the in- dividual social interaction scores as the outcomes

This section provides the results for each of the individual social interaction scores as the outcome in the models.

## The full interaction model with Acknowledgment as the outcome

Table 1.2.1. The full interaction model with Acknowledgment as the outcome, N = 43

|  | Estimate | Std. Error | df | t value | p-value | q-value |
| --- | --- | --- | --- | --- | --- | --- |
| (Intercept) | 4.24 | 0.25 | 58.76 | 17.22 | 0.00 | 0 |
| oxytocin | -0.45 | 0.39 | 58.76 | -1.16 | 0.25 | 1 |
| social treatment | -0.38 | 0.36 | 58.76 | -1.04 | 0.30 | 1 |
| time | 0.00 | 0.08 | 39.00 | 0.00 | 1.00 | 1 |
| oxytocin×social treatment | 0.38 | 0.55 | 58.76 | 0.70 | 0.49 | 1 |
| oxytocin×time | 0.12 | 0.12 | 39.00 | 1.04 | 0.31 | 1 |
| social treatment×time | 0.03 | 0.11 | 39.00 | 0.22 | 0.82 | 1 |
| oxytocin×social treatment×time | -0.08 | 0.17 | 39.00 | -0.45 | 0.65 | 1 |

social skills training

4.5

supportive psychotherapy

4.0

Acknowledgment

oxytocin

placebo oxytocin

3.5

0 1 2 3 0 1 2 3

time

Figure 1.2.1 The predicted values of the full interaction model with Acknowledgment as the outcome

## The full interaction model with Alert as the outcome

Table 1.2.2. The full interaction model with Alert as the outcome, N = 43

supportive psychotherapy

|  | Estimate | Std. Error | df | t value | p-value | q-value |
| --- | --- | --- | --- | --- | --- | --- |
| (Intercept) | 3.10 | 0.32 | 49.52 | 9.65 | 0.00 | 0 |
| oxytocin | -0.23 | 0.50 | 49.52 | -0.46 | 0.65 | 1 |
| social treatment | -0.60 | 0.47 | 49.52 | -1.27 | 0.21 | 1 |
| time | -0.03 | 0.07 | 39.00 | -0.46 | 0.65 | 1 |
| oxytocin×social treatment | 0.65 | 0.71 | 49.52 | 0.91 | 0.37 | 1 |
| oxytocin×time | 0.15 | 0.12 | 39.00 | 1.30 | 0.20 | 1 |
| social treatment×time | 0.17 | 0.11 | 39.00 | 1.55 | 0.13 | 1 |
| oxytocin×social treatment×time | -0.33 | 0.17 | 39.00 | -1.98 | 0.06 | 1 |

social skills training

4.0

3.5

3.0

Alert

oxytocin

placebo oxytocin

2.5

2.0

0 1 2 3 0 1 2 3

time

Figure 1.2.2 The predicted values of the full interaction model with Alert as the outcome

## The full interaction model with Anger as the outcome

Table 1.2.3. The full interaction model with Anger as the outcome, N = 43

|  | Estimate | Std. Error | df | t value | p-value | q-value |
| --- | --- | --- | --- | --- | --- | --- |
| (Intercept) | 1.05 | 0.06 | 77.28 | 18.90 | 0.00 | 0 |
| oxytocin | 0.08 | 0.09 | 77.28 | 0.90 | 0.37 | 1 |
| social treatment | -0.04 | 0.08 | 77.28 | -0.44 | 0.66 | 1 |
| time | 0.01 | 0.02 | 39.00 | 0.34 | 0.73 | 1 |
| oxytocin×social treatment | 0.06 | 0.12 | 77.28 | 0.46 | 0.65 | 1 |
| oxytocin×time | 0.01 | 0.04 | 39.00 | 0.26 | 0.80 | 1 |
| social treatment×time | 0.02 | 0.04 | 39.00 | 0.59 | 0.56 | 1 |
| oxytocin×social treatment×time | -0.05 | 0.06 | 39.00 | -0.93 | 0.36 | 1 |

social skills training

supportive psychotherapy

1.2

1.1

Anger

oxytocin

placebo oxytocin

1.0

0.9

0 1 2 3 0 1 2 3

time

Figure 1.2.3 The predicted values of the full interaction model with Anger as the outcome

## 1.2.4 The full interaction model with Anxiety as the outcome

Table 1.2.4. The full interaction model with Anxiety as the outcome, N = 43

supportive psychotherapy

|  | Estimate | Std. Error | df | t value | p-value | q-value |
| --- | --- | --- | --- | --- | --- | --- |
| (Intercept) | 1.40 | 0.14 | 53.76 | 10.03 | 0.00 | 0 |
| oxytocin | -0.01 | 0.22 | 53.76 | -0.04 | 0.97 | 1 |
| social treatment | 0.19 | 0.21 | 53.76 | 0.94 | 0.35 | 1 |
| time | -0.02 | 0.04 | 39.00 | -0.45 | 0.65 | 1 |
| oxytocin×social treatment | -0.25 | 0.31 | 53.76 | -0.81 | 0.42 | 1 |
| oxytocin×time | 0.05 | 0.06 | 39.00 | 0.81 | 0.42 | 1 |
| social treatment×time | 0.06 | 0.06 | 39.00 | 1.13 | 0.27 | 1 |
| oxytocin×social treatment×time | -0.17 | 0.08 | 39.00 | -1.98 | 0.05 | 1 |

social skills training

2.0

1.6

oxytocin

Anxiety

placebo oxytocin

1.2

0.8

0 1 2 3 0 1 2 3

time

Figure 1.2.4 The predicted values of the full interaction model with Anxiety as the outcome

## The full interaction model with Avoidance as the outcome

Table 1.2.5. The full interaction model with Avoidance as the outcome, N = 43

|  | Estimate | Std. Error | df | t value | p-value | q-value |
| --- | --- | --- | --- | --- | --- | --- |
| (Intercept) | 1.09 | 0.07 | 61.85 | 15.87 | 0.00 | 0.00 |
| oxytocin | -0.07 | 0.11 | 61.85 | -0.66 | 0.51 | 1.00 |
| social treatment | -0.01 | 0.10 | 61.85 | -0.14 | 0.89 | 1.00 |
| time | -0.02 | 0.02 | 39.00 | -0.94 | 0.35 | 1.00 |
| oxytocin×social treatment | 0.26 | 0.15 | 61.85 | 1.72 | 0.09 | 1.00 |
| oxytocin×time | 0.07 | 0.04 | 39.00 | 2.00 | 0.05 | 1.00 |
| social treatment×time | 0.07 | 0.03 | 39.00 | 2.00 | 0.05 | 1.00 |
| oxytocin×social treatment×time | -0.14 | 0.05 | 39.00 | -2.87 | 0.01 | 0.18 |

social skills training

supportive psychotherapy

1.2

Avoidance

oxytocin

placebo oxytocin

1.0

0 1 2 3 0 1 2 3

time

Figure 1.2.5 The predicted values of the full interaction model with Avoidance as the outcome

## The full interaction model with Blunted affect as the outcome

Table 1.2.6. The full interaction model with Blunted affect as the outcome, N = 43

|  | Estimate | Std. Error | df | t value | p-value | q-value |
| --- | --- | --- | --- | --- | --- | --- |
| (Intercept) | 2.00 | 0.22 | 45.75 | 9.13 | 0.00 | 0 |
| oxytocin | 0.00 | 0.34 | 45.75 | 0.00 | 1.00 | 1 |
| social treatment | 0.09 | 0.32 | 45.75 | 0.28 | 0.78 | 1 |
| time | 0.05 | 0.04 | 39.00 | 1.24 | 0.22 | 1 |
| oxytocin×social treatment | -0.16 | 0.49 | 45.75 | -0.32 | 0.75 | 1 |
| oxytocin×time | -0.04 | 0.06 | 39.00 | -0.60 | 0.55 | 1 |
| social treatment×time | -0.13 | 0.06 | 39.00 | -2.08 | 0.04 | 1 |
| oxytocin×social treatment×time | 0.04 | 0.09 | 39.00 | 0.46 | 0.65 | 1 |

social skills training

2.4

supportive psychotherapy

2.0

Blunted affect

oxytocin

placebo oxytocin

1.6

1.2

0 1 2 3

time

0 1 2 3

Figure 1.2.6 The predicted values of the full interaction model with Blunted affect as the outcome

## 1.2.7 The full interaction model with Constricted as the outcome

Table 1.2.7. The full interaction model with Constricted as the outcome, N = 43

|  | Estimate | Std. Error | df | t value | p-value | q-value |
| --- | --- | --- | --- | --- | --- | --- |
| (Intercept) | 1.87 | 0.21 | 53.47 | 8.87 | 0.00 | 0 |
| oxytocin | 0.28 | 0.33 | 53.47 | 0.84 | 0.41 | 1 |
| social treatment | 0.23 | 0.31 | 53.47 | 0.75 | 0.46 | 1 |
| time | 0.03 | 0.06 | 39.00 | 0.60 | 0.55 | 1 |
| oxytocin×social treatment | -0.35 | 0.47 | 53.47 | -0.75 | 0.46 | 1 |
| oxytocin×time | -0.13 | 0.09 | 39.00 | -1.43 | 0.16 | 1 |
| social treatment×time | -0.08 | 0.08 | 39.00 | -0.95 | 0.35 | 1 |
| oxytocin×social treatment×time | 0.12 | 0.13 | 39.00 | 0.93 | 0.36 | 1 |

social skills training

2.4

supportive psychotherapy

2.0

Constricted

oxytocin

placebo oxytocin

1.6

0 1 2 3 0 1 2 3

time

Figure 1.2.7 The predicted values of the full interaction model with Constricted as the outcome

## The full interaction model with Criticism as the outcome

Table 1.2.8. The full interaction model with Criticism as the outcome, N = 43

supportive psychotherapy

|  | Estimate | Std. Error | df | t value | p-value | q-value |
| --- | --- | --- | --- | --- | --- | --- |
| (Intercept) | 1.06 | 0.07 | 77.6 | 14.28 | 0.00 | 0 |
| oxytocin | 0.14 | 0.12 | 77.6 | 1.20 | 0.23 | 1 |
| social treatment | 0.04 | 0.11 | 77.6 | 0.38 | 0.70 | 1 |
| time | 0.06 | 0.03 | 39.0 | 1.89 | 0.07 | 1 |
| oxytocin×social treatment | -0.05 | 0.17 | 77.6 | -0.28 | 0.78 | 1 |
| oxytocin×time | -0.08 | 0.05 | 39.0 | -1.44 | 0.16 | 1 |
| social treatment×time | -0.06 | 0.05 | 39.0 | -1.28 | 0.21 | 1 |
| oxytocin×social treatment×time | 0.08 | 0.08 | 39.0 | 1.09 | 0.28 | 1 |

social skills training

1.4

1.3

- 1. oxytocin

Criticism

placebo oxytocin

1.1

1.0

0.9

0 1 2 3

time

0 1 2 3

Figure 1.2.8 The predicted values of the full interaction model with Criticism as the outcome

## The full interaction model with Detachment as the outcome

Table 1.2.9. The full interaction model with Detachment as the outcome, N = 43

supportive psychotherapy

|  | Estimate | Std. Error | df | t value | p-value | q-value |
| --- | --- | --- | --- | --- | --- | --- |
| (Intercept) | 1.06 | 0.04 | 61.36 | 29.84 | 0.00 | 0 |
| oxytocin | 0.01 | 0.06 | 61.36 | 0.18 | 0.86 | 1 |
| social treatment | -0.02 | 0.05 | 61.36 | -0.35 | 0.72 | 1 |
| time | -0.01 | 0.01 | 39.00 | -0.73 | 0.47 | 1 |
| oxytocin×social treatment | -0.06 | 0.08 | 61.36 | -0.70 | 0.49 | 1 |
| oxytocin×time | 0.02 | 0.02 | 39.00 | 1.15 | 0.26 | 1 |
| social treatment×time | 0.01 | 0.02 | 39.00 | 0.79 | 0.43 | 1 |
| oxytocin×social treatment×time | -0.01 | 0.03 | 39.00 | -0.36 | 0.72 | 1 |

social skills training

1.2

1.1

Detachment

oxytocin

placebo oxytocin

1.0

0 1 2 3 0 1 2 3

time

Figure 1.2.9 The predicted values of the full interaction model with Detachment as the outcome

## The full interaction model with Elaboration as the outcome

Table 1.2.10. The full interaction model with Elaboration as the outcome, N = 43

|  | Estimate | Std. Error | df | t value | p-value | q-value |
| --- | --- | --- | --- | --- | --- | --- |
| (Intercept) | 3.55 | 0.23 | 56.97 | 15.21 | 0.00 | 0 |
| oxytocin | -0.46 | 0.37 | 56.97 | -1.26 | 0.21 | 1 |
| social treatment | 0.01 | 0.34 | 56.97 | 0.03 | 0.98 | 1 |
| time | 0.00 | 0.07 | 39.00 | 0.00 | 1.00 | 1 |
| oxytocin×social treatment | 0.45 | 0.52 | 56.97 | 0.86 | 0.39 | 1 |
| oxytocin×time | 0.09 | 0.11 | 39.00 | 0.86 | 0.40 | 1 |
| social treatment×time | -0.01 | 0.10 | 39.00 | -0.05 | 0.96 | 1 |
| oxytocin×social treatment×time | -0.05 | 0.15 | 39.00 | -0.32 | 0.75 | 1 |

social skills training

4.0

supportive psychotherapy

3.5 oxytocin

Elaboration

placebo oxytocin

3.0

2.5

0 1 2 3

time

0 1 2 3

Figure 1.2.10 The predicted values of the full interaction model with Elaboration as the outcome

## The full interaction model with Fluency as the outcome

Table 1.2.11. The full interaction model with Fluency as the outcome, N = 43

supportive psychotherapy

|  | Estimate | Std. Error | df | t value | p-value | q-value |
| --- | --- | --- | --- | --- | --- | --- |
| (Intercept) | 4.10 | 0.20 | 52.7 | 20.04 | 0.00 | 0 |
| oxytocin | -0.60 | 0.32 | 52.7 | -1.88 | 0.07 | 1 |
| social treatment | -0.33 | 0.30 | 52.7 | -1.09 | 0.28 | 1 |
| time | -0.01 | 0.05 | 39.0 | -0.24 | 0.81 | 1 |
| oxytocin×social treatment | 0.53 | 0.45 | 52.7 | 1.17 | 0.25 | 1 |
| oxytocin×time | 0.15 | 0.08 | 39.0 | 1.78 | 0.08 | 1 |
| social treatment×time | 0.09 | 0.08 | 39.0 | 1.19 | 0.24 | 1 |
| oxytocin×social treatment×time | -0.15 | 0.12 | 39.0 | -1.23 | 0.23 | 1 |

social skills training

4.5

4.0

oxytocin

Fluency

placebo oxytocin

3.5

3.0

0 1 2 3

time

0 1 2 3

Figure 1.2.11 The predicted values of the full interaction model with Fluency as the outcome

## The full interaction model with Gaze as the outcome

Table 1.2.12. The full interaction model with Gaze as the outcome, N = 43

|  | Estimate | Std. Error | df | t value | p-value | q-value |
| --- | --- | --- | --- | --- | --- | --- |
| (Intercept) | 3.31 | 0.31 | 49.27 | 10.62 | 0.00 | 0 |
| oxytocin | -0.44 | 0.49 | 49.27 | -0.90 | 0.37 | 1 |
| social treatment | -0.87 | 0.46 | 49.27 | -1.89 | 0.07 | 1 |
| time | -0.05 | 0.07 | 39.00 | -0.72 | 0.48 | 1 |
| oxytocin×social treatment | 1.06 | 0.69 | 49.27 | 1.54 | 0.13 | 1 |
| oxytocin×time | -0.05 | 0.11 | 39.00 | -0.48 | 0.63 | 1 |
| social treatment×time | 0.19 | 0.11 | 39.00 | 1.78 | 0.08 | 1 |
| oxytocin×social treatment×time | 0.07 | 0.16 | 39.00 | 0.42 | 0.67 | 1 |

social skills training

4.0

supportive psychotherapy

3.5

oxytocin

Gaze

3.0 placebo

oxytocin

2.5

2.0

0 1 2 3 0 1 2 3

time

Figure 1.2.12 The predicted values of the full interaction model with Gaze as the outcome

## The full interaction model with Hostility as the outcome

Table 1.2.13. The full interaction model with Hostility as the outcome, N = 43

|  | Estimate | Std. Error | df | t value | p-value | q-value |
| --- | --- | --- | --- | --- | --- | --- |
| (Intercept) | 1.00 | 0.02 | 78 | 40.44 | 0.00 | 0 |
| oxytocin | 0.00 | 0.04 | 78 | 0.00 | 1.00 | 1 |
| social treatment | 0.00 | 0.04 | 78 | 0.00 | 1.00 | 1 |
| time | 0.00 | 0.01 | 78 | 0.00 | 1.00 | 1 |
| oxytocin×social treatment | 0.05 | 0.05 | 78 | 0.91 | 0.37 | 1 |
| oxytocin×time | 0.02 | 0.02 | 78 | 1.35 | 0.18 | 1 |
| social treatment×time | 0.00 | 0.02 | 78 | 0.00 | 1.00 | 1 |
| oxytocin×social treatment×time | -0.04 | 0.03 | 78 | -1.60 | 0.11 | 1 |

social skills training

1.10

supportive psychotherapy

1.05

Hostility

oxytocin

placebo oxytocin

1.00

0.95

0 1 2 3 0 1 2 3

time

Figure 1.2.13 The predicted values of the full interaction model with Hostility as the outcome

## 1.2.14 The full interaction model with Initiation as the outcome

Table 1.2.14. The full interaction model with Initiation as the outcome, N = 43

|  | Estimate | Std. Error | df | t value | p-value | q-value |
| --- | --- | --- | --- | --- | --- | --- |
| (Intercept) | 2.77 | 0.28 | 67.19 | 9.93 | 0.00 | 0 |
| oxytocin | -0.44 | 0.44 | 67.19 | -1.00 | 0.32 | 1 |
| social treatment | 0.37 | 0.41 | 67.19 | 0.89 | 0.38 | 1 |
| time | -0.11 | 0.10 | 39.00 | -1.07 | 0.29 | 1 |
| oxytocin×social treatment | 0.32 | 0.62 | 67.19 | 0.52 | 0.60 | 1 |
| oxytocin×time | 0.24 | 0.16 | 39.00 | 1.50 | 0.14 | 1 |
| social treatment×time | 0.09 | 0.15 | 39.00 | 0.62 | 0.54 | 1 |
| oxytocin×social treatment×time | -0.27 | 0.23 | 39.00 | -1.21 | 0.23 | 1 |

social skills training

3.5

supportive psychotherapy

3.0

Initiation

2.5

oxytocin

placebo oxytocin

2.0

0 1 2 3 0 1 2 3

time

Figure 1.2.14 The predicted values of the full interaction model with Initiation as the outcome

## 1.2.15 The full interaction model with Intrusiveness as the outcome

Table 1.2.15. The full interaction model with Intrusiveness as the outcome, N = 43

supportive psychotherapy

|  | Estimate | Std. Error | df | t value | p-value | q-value |
| --- | --- | --- | --- | --- | --- | --- |
| (Intercept) | 1.21 | 0.09 | 56.28 | 13.54 | 0.00 | 0 |
| oxytocin | 0.20 | 0.14 | 56.28 | 1.45 | 0.15 | 1 |
| social treatment | -0.13 | 0.13 | 56.28 | -0.98 | 0.33 | 1 |
| time | 0.00 | 0.03 | 39.00 | 0.17 | 0.87 | 1 |
| oxytocin×social treatment | -0.21 | 0.20 | 56.28 | -1.07 | 0.29 | 1 |
| oxytocin×time | -0.04 | 0.04 | 39.00 | -1.02 | 0.31 | 1 |
| social treatment×time | 0.01 | 0.04 | 39.00 | 0.15 | 0.88 | 1 |
| oxytocin×social treatment×time | 0.04 | 0.06 | 39.00 | 0.64 | 0.52 | 1 |

social skills training

1.6

1.4

1.2

Intrusiveness

oxytocin

placebo oxytocin

1.0

0 1 2 3 0 1 2 3

time

Figure 1.2.15 The predicted values of the full interaction model with Intrusiveness as the outcome

## 1.2.16 The full interaction model with Lead Expert as the outcome

Table 1.2.16. The full interaction model with Lead Expert as the outcome, N = 43

supportive psychotherapy

|  | Estimate | Std. Error | df | t value | p-value | q-value |
| --- | --- | --- | --- | --- | --- | --- |
| (Intercept) | 3.35 | 0.19 | 55.29 | 17.19 | 0.00 | 0 |
| oxytocin | -0.01 | 0.30 | 55.29 | -0.04 | 0.97 | 1 |
| social treatment | -0.01 | 0.29 | 55.29 | -0.04 | 0.96 | 1 |
| time | 0.03 | 0.05 | 39.00 | 0.47 | 0.64 | 1 |
| oxytocin×social treatment | 0.26 | 0.43 | 55.29 | 0.61 | 0.55 | 1 |
| oxytocin×time | -0.01 | 0.09 | 39.00 | -0.08 | 0.93 | 1 |
| social treatment×time | 0.00 | 0.08 | 39.00 | 0.00 | 1.00 | 1 |
| oxytocin×social treatment×time | -0.07 | 0.12 | 39.00 | -0.56 | 0.58 | 1 |

social skills training

4.0

3.6

Lead Expert

oxytocin

placebo oxytocin

3.2

0 1 2 3 0 1 2 3

time

Figure 1.2.16 The predicted values of the full interaction model with Lead Expert as the outcome

## 1.2.17 The full interaction model with Lead Patient as the outcome

Table 1.2.17. The full interaction model with Lead Patient as the outcome, N = 43

|  | Estimate | Std. Error | df | t value | p-value | q-value |
| --- | --- | --- | --- | --- | --- | --- |
| (Intercept) | 3.01 | 0.20 | 50.63 | 15.17 | 0.00 | 0 |
| oxytocin | -0.31 | 0.31 | 50.63 | -1.00 | 0.32 | 1 |
| social treatment | -0.21 | 0.29 | 50.63 | -0.72 | 0.48 | 1 |
| time | -0.03 | 0.05 | 39.00 | -0.62 | 0.54 | 1 |
| oxytocin×social treatment | 0.09 | 0.44 | 50.63 | 0.20 | 0.84 | 1 |
| oxytocin×time | 0.05 | 0.08 | 39.00 | 0.73 | 0.47 | 1 |
| social treatment×time | 0.02 | 0.07 | 39.00 | 0.28 | 0.78 | 1 |
| oxytocin×social treatment×time | -0.01 | 0.11 | 39.00 | -0.05 | 0.96 | 1 |

social skills training

3.0

supportive psychotherapy

oxytocin

Lead Patient

placebo oxytocin

2.5

0 1 2 3 0 1 2 3

time

Figure 1.2.17 The predicted values of the full interaction model with Lead Patient as the outcome

## 1.2.18 The full interaction model with Mismatched Affect as the outcome

Table 1.2.18. The full interaction model with Mismatched Affect as the outcome, N = 43

|  | Estimate | Std. Error | df | t value | p-value | q-value |
| --- | --- | --- | --- | --- | --- | --- |
| (Intercept) | 1.27 | 0.09 | 58.95 | 14.85 | 0.00 | 0 |
| oxytocin | -0.21 | 0.13 | 58.95 | -1.60 | 0.12 | 1 |
| social treatment | -0.06 | 0.13 | 58.95 | -0.51 | 0.61 | 1 |
| time | -0.03 | 0.03 | 39.00 | -1.21 | 0.23 | 1 |
| oxytocin×social treatment | 0.18 | 0.19 | 58.95 | 0.97 | 0.34 | 1 |
| oxytocin×time | 0.03 | 0.04 | 39.00 | 0.77 | 0.44 | 1 |
| social treatment×time | 0.03 | 0.04 | 39.00 | 0.82 | 0.42 | 1 |
| oxytocin×social treatment×time | -0.06 | 0.06 | 39.00 | -0.97 | 0.34 | 1 |

social skills training

1.4

supportive psychotherapy

1.2

Mismatched Affect

oxytocin

placebo oxytocin

1.0

0 1 2 3 0 1 2 3

time

Figure 1.2.18 The predicted values of the full interaction model with Mismatched Affect as the outcome

## The full interaction model with Motivation as the outcome

Table 1.2.19. The full interaction model with Motivation as the outcome, N = 43

|  | Estimate | Std. Error | df | t value | p-value | q-value |
| --- | --- | --- | --- | --- | --- | --- |
| (Intercept) | 4.27 | 0.21 | 56.28 | 20.22 | 0.00 | 0 |
| oxytocin | -0.36 | 0.33 | 56.28 | -1.10 | 0.28 | 1 |
| social treatment | -0.62 | 0.31 | 56.28 | -1.98 | 0.05 | 1 |
| time | 0.04 | 0.06 | 39.00 | 0.63 | 0.53 | 1 |
| oxytocin×social treatment | 0.74 | 0.47 | 56.28 | 1.59 | 0.12 | 1 |
| oxytocin×time | 0.05 | 0.10 | 39.00 | 0.57 | 0.58 | 1 |
| social treatment×time | 0.15 | 0.09 | 39.00 | 1.64 | 0.11 | 1 |
| oxytocin×social treatment×time | -0.25 | 0.14 | 39.00 | -1.81 | 0.08 | 1 |

social skills training

4.5

supportive psychotherapy

oxytocin

Motivation

4.0 placebo

oxytocin

3.5

0 1 2 3 0 1 2 3

time

Figure 1.2.19 The predicted values of the full interaction model with Motivation as the outcome

## The full interaction model with negative affect as the outcome

Table 1.2.20. The full interaction model with negative affect as the outcome, N = 43

|  | Estimate | Std. Error | df | t value | p-value | q-value |
| --- | --- | --- | --- | --- | --- | --- |
| (Intercept) | 1.40 | 0.17 | 69.64 | 8.15 | 0.00 | 0.00 |
| oxytocin | 0.82 | 0.27 | 69.64 | 3.08 | 0.00 | 0.08 |
| social treatment | 0.03 | 0.25 | 69.64 | 0.11 | 0.92 | 1.00 |
| time | 0.02 | 0.07 | 39.00 | 0.33 | 0.75 | 1.00 |
| oxytocin×social treatment | -0.82 | 0.38 | 69.64 | -2.14 | 0.04 | 0.96 |
| oxytocin×time | -0.19 | 0.10 | 39.00 | -1.90 | 0.06 | 1.00 |
| social treatment×time | 0.02 | 0.10 | 39.00 | 0.25 | 0.80 | 1.00 |
| oxytocin×social treatment×time | 0.13 | 0.15 | 39.00 | 0.87 | 0.39 | 1.00 |

social skills training

2.5

supportive psychotherapy

2.0

negative affect

oxytocin

placebo oxytocin

1.5

1.0

0 1 2 3 0 1 2 3

time

Figure 1.2.20 The predicted values of the full interaction model with negative affect as the outcome

## 1.2.21 The full interaction model with Positive Affect as the outcome

Table 1.2.21. The full interaction model with Positive Affect as the outcome, N = 43

supportive psychotherapy

|  | Estimate | Std. Error | df | t value | p-value | q-value |
| --- | --- | --- | --- | --- | --- | --- |
| (Intercept) | 2.04 | 0.18 | 51.09 | 11.41 | 0.00 | 0.00 |
| oxytocin | -0.67 | 0.28 | 51.09 | -2.39 | 0.02 | 0.55 |
| social treatment | -0.57 | 0.26 | 51.09 | -2.16 | 0.04 | 0.97 |
| time | 0.02 | 0.04 | 39.00 | 0.48 | 0.63 | 1.00 |
| oxytocin×social treatment | 0.75 | 0.40 | 51.09 | 1.89 | 0.06 | 1.00 |
| oxytocin×time | 0.13 | 0.07 | 39.00 | 1.93 | 0.06 | 1.00 |
| social treatment×time | 0.06 | 0.07 | 39.00 | 0.99 | 0.33 | 1.00 |
| oxytocin×social treatment×time | -0.18 | 0.10 | 39.00 | -1.84 | 0.07 | 1.00 |

social skills training

2.5

2.0

oxytocin

Positive Affect

placebo oxytocin

1.5

1.0

0 1 2 3 0 1 2 3

time

Figure 1.2.21 The predicted values of the full interaction model with Positive Affect as the outcome

## 1.2.22 The full interaction model with Persistence as the outcome

Table 1.2.22. The full interaction model with Persistence as the outcome, N = 43

|  | Estimate | Std. Error | df | t value | p-value | q-value |
| --- | --- | --- | --- | --- | --- | --- |
| (Intercept) | 4.49 | 0.16 | 59.13 | 28.10 | 0.00 | 0 |
| oxytocin | 0.14 | 0.25 | 59.13 | 0.57 | 0.57 | 1 |
| social treatment | 0.27 | 0.24 | 59.13 | 1.15 | 0.26 | 1 |
| time | 0.07 | 0.05 | 39.00 | 1.46 | 0.15 | 1 |
| oxytocin×social treatment | -0.25 | 0.35 | 59.13 | -0.71 | 0.48 | 1 |
| oxytocin×time | -0.05 | 0.08 | 39.00 | -0.70 | 0.49 | 1 |
| social treatment×time | -0.10 | 0.07 | 39.00 | -1.33 | 0.19 | 1 |
| oxytocin×social treatment×time | 0.03 | 0.11 | 39.00 | 0.32 | 0.75 | 1 |

social skills training

5.0

supportive psychotherapy

4.8

4.6

Persistence

oxytocin

placebo oxytocin

4.4

4.2

0 1 2 3 0 1 2 3

time

Figure 1.2.22 The predicted values of the full interaction model with Persistence as the outcome

## 1.2.23 The full interaction model with Reciprocity as the outcome

Table 1.2.23. The full interaction model with Reciprocity as the outcome, N = 43

|  | Estimate | Std. Error | df | t value | p-value | q-value |
| --- | --- | --- | --- | --- | --- | --- |
| (Intercept) | 4.22 | 0.18 | 54.5 | 23.51 | 0.00 | 0 |
| oxytocin | -0.42 | 0.28 | 54.5 | -1.50 | 0.14 | 1 |
| social treatment | -0.14 | 0.26 | 54.5 | -0.54 | 0.59 | 1 |
| time | 0.00 | 0.05 | 39.0 | 0.09 | 0.93 | 1 |
| oxytocin×social treatment | 0.41 | 0.40 | 54.5 | 1.04 | 0.30 | 1 |
| oxytocin×time | 0.05 | 0.08 | 39.0 | 0.66 | 0.51 | 1 |
| social treatment×time | 0.04 | 0.07 | 39.0 | 0.49 | 0.62 | 1 |
| oxytocin×social treatment×time | -0.05 | 0.11 | 39.0 | -0.48 | 0.63 | 1 |

social skills training

4.4

supportive psychotherapy

4.0

Reciprocity

oxytocin

placebo oxytocin

3.6

0 1 2 3 0 1 2 3

time

Figure 1.2.23 The predicted values of the full interaction model with Reciprocity as the outcome

## The full interaction model with Silence as the outcome

Table 1.2.24. The full interaction model with Silence as the outcome, N = 43

|  | Estimate | Std. Error | df | t value | p-value | q-value |
| --- | --- | --- | --- | --- | --- | --- |
| (Intercept) | 1.38 | 0.15 | 49.39 | 9.52 | 0.00 | 0 |
| oxytocin | 0.19 | 0.23 | 49.39 | 0.83 | 0.41 | 1 |
| social treatment | 0.09 | 0.21 | 49.39 | 0.40 | 0.69 | 1 |
| time | -0.02 | 0.03 | 39.00 | -0.51 | 0.61 | 1 |
| oxytocin×social treatment | -0.41 | 0.32 | 49.39 | -1.27 | 0.21 | 1 |
| oxytocin×time | 0.00 | 0.05 | 39.00 | 0.09 | 0.93 | 1 |
| social treatment×time | 0.02 | 0.05 | 39.00 | 0.35 | 0.73 | 1 |
| oxytocin×social treatment×time | 0.03 | 0.07 | 39.00 | 0.46 | 0.65 | 1 |

social skills training

1.75

supportive psychotherapy

1.50 oxytocin

Silence

placebo oxytocin

1.25

1.00

0 1 2 3 0 1 2 3

time

Figure 1.2.24 The predicted values of the full interaction model with Silence as the outcome

## The full interaction model with Synchrony as the outcome

Table 1.2.25. The full interaction model with Synchrony as the outcome, N = 43

supportive psychotherapy

|  | Estimate | Std. Error | df | t value | p-value | q-value |
| --- | --- | --- | --- | --- | --- | --- |
| (Intercept) | 4.14 | 0.18 | 57.77 | 22.47 | 0.00 | 0 |
| oxytocin | -0.38 | 0.29 | 57.77 | -1.32 | 0.19 | 1 |
| social treatment | -0.20 | 0.27 | 57.77 | -0.74 | 0.46 | 1 |
| time | -0.01 | 0.06 | 39.00 | -0.15 | 0.88 | 1 |
| oxytocin×social treatment | 0.28 | 0.41 | 57.77 | 0.67 | 0.50 | 1 |
| oxytocin×time | 0.10 | 0.09 | 39.00 | 1.17 | 0.25 | 1 |
| social treatment×time | 0.06 | 0.08 | 39.00 | 0.78 | 0.44 | 1 |
| oxytocin×social treatment×time | -0.12 | 0.12 | 39.00 | -1.00 | 0.32 | 1 |

social skills training

4.5

4.2

3.9

Synchrony

oxytocin

placebo oxytocin

3.6

3.3

0 1 2 3

time

0 1 2 3

Figure 1.2.25 The predicted values of the full interaction model with Synchrony as the outcome

## 1.2.26 The full interaction model with Tension as the outcome

Table 1.2.26. The full interaction model with Tension as the outcome, N = 43

|  | Estimate | Std. Error | df | t value | p-value | q-value |
| --- | --- | --- | --- | --- | --- | --- |
| (Intercept) | 1.19 | 0.16 | 54.74 | 7.68 | 0.00 | 0.00 |
| oxytocin | 0.47 | 0.24 | 54.74 | 1.95 | 0.06 | 1.00 |
| social treatment | 0.52 | 0.23 | 54.74 | 2.27 | 0.03 | 0.74 |
| time | 0.05 | 0.04 | 39.00 | 1.09 | 0.28 | 1.00 |
| oxytocin×social treatment | -0.59 | 0.34 | 54.74 | -1.70 | 0.09 | 1.00 |
| oxytocin×time | -0.11 | 0.07 | 39.00 | -1.70 | 0.10 | 1.00 |
| social treatment×time | -0.10 | 0.06 | 39.00 | -1.53 | 0.13 | 1.00 |
| oxytocin×social treatment×time | 0.08 | 0.10 | 39.00 | 0.80 | 0.43 | 1.00 |

2.1

supportive psychotherapy

social skills training

1.8

1.5

Tension

oxytocin

placebo oxytocin

1.2

0.9

0 1 2 3 0 1 2 3

time

Figure 1.2.26 The predicted values of the full interaction model with Tension as the outcome

## 1.2.27 The full interaction model with Withdrawal as the outcome

Table 1.2.27. The full interaction model with Withdrawal as the outcome, N = 43

supportive psychotherapy

|  | Estimate | Std. Error | df | t value | p-value | q-value |
| --- | --- | --- | --- | --- | --- | --- |
| (Intercept) | 1.31 | 0.11 | 74.51 | 11.65 | 0.00 | 0 |
| oxytocin | 0.06 | 0.18 | 74.51 | 0.36 | 0.72 | 1 |
| social treatment | 0.19 | 0.17 | 74.51 | 1.16 | 0.25 | 1 |
| time | -0.03 | 0.05 | 39.00 | -0.55 | 0.59 | 1 |
| oxytocin×social treatment | -0.03 | 0.25 | 74.51 | -0.12 | 0.91 | 1 |
| oxytocin×time | 0.00 | 0.07 | 39.00 | 0.01 | 0.99 | 1 |
| social treatment×time | -0.01 | 0.07 | 39.00 | -0.14 | 0.89 | 1 |
| oxytocin×social treatment×time | 0.01 | 0.10 | 39.00 | 0.06 | 0.95 | 1 |

social skills training

1.8

1.6

oxytocin

Withdrawal

1.4 placebo

oxytocin

1.2

1.0

0 1 2 3

time

0 1 2 3

Figure 1.2.27 The predicted values of the full interaction model with Withdrawal as the outcome

# The oxytocin–social-treatment full interaction models: the PANSS scores as the outcomes

This section provides the results for each of the PANSS scores as the outcome in the models.

## The full interaction model with PANSS total as the outcome

Table 1.3.1. The full interaction model with PANSS total as the outcome, N = 51

supportive psychotherapy

|  | Estimate | Std. Error | df | t value | p-value | q-value |
| --- | --- | --- | --- | --- | --- | --- |
| (Intercept) | 63.62 | 3.95 | 54.52 | 16.10 | 0.00 | 0.00 |
| oxytocin | 8.39 | 5.71 | 54.77 | 1.47 | 0.15 | 1.00 |
| social treatment | 2.58 | 5.59 | 54.79 | 0.46 | 0.65 | 1.00 |
| time | -2.50 | 0.79 | 135.15 | -3.18 | 0.00 | 0.02 |
| oxytocin×social treatment | -7.39 | 8.00 | 55.00 | -0.92 | 0.36 | 1.00 |
| oxytocin×time | 0.55 | 1.16 | 136.07 | 0.48 | 0.63 | 1.00 |
| social treatment×time | -0.45 | 1.13 | 135.59 | -0.40 | 0.69 | 1.00 |
| oxytocin×social treatment×time | 0.25 | 1.64 | 136.22 | 0.15 | 0.88 | 1.00 |

social skills training

80

70

oxytocin

PANSS total

placebo oxytocin

60

50

0 1 2 3 0 1 2 3

week

Figure 1.3.1 The predicted values of the full interaction model with PANSS total as the outcome

## The full interaction model with P (PANSS) as the outcome

Table 1.3.2. The full interaction model with P (PANSS) as the outcome, N = 51

supportive psychotherapy

|  | Estimate | Std. Error | df | t value | p-value | q-value |
| --- | --- | --- | --- | --- | --- | --- |
| (Intercept) | 12.76 | 1.29 | 53.54 | 9.92 | 0.00 | 0.00 |
| oxytocin | 1.97 | 1.86 | 53.80 | 1.06 | 0.29 | 1.00 |
| social treatment | -0.48 | 1.82 | 53.82 | -0.26 | 0.80 | 1.00 |
| time | -0.89 | 0.26 | 133.92 | -3.42 | 0.00 | 0.01 |
| oxytocin×social treatment | -0.22 | 2.61 | 54.03 | -0.09 | 0.93 | 1.00 |
| oxytocin×time | 0.64 | 0.38 | 134.88 | 1.67 | 0.10 | 0.87 |
| social treatment×time | -0.04 | 0.38 | 134.38 | -0.09 | 0.92 | 1.00 |
| oxytocin×social treatment×time | -0.14 | 0.54 | 135.03 | -0.27 | 0.79 | 1.00 |

social skills training

18

15

oxytocin

P (PANSS)

placebo

12 oxytocin

9

0 1 2 3 0 1 2 3

week

Figure 1.3.2 The predicted values of the full interaction model with P (PANSS) as the outcome

## The full interaction model with N (PANSS) as the outcome

Table 1.3.3. The full interaction model with N (PANSS) as the outcome, N = 51

|  | Estimate | Std. Error | df | t value | p-value | q-value |
| --- | --- | --- | --- | --- | --- | --- |
| (Intercept) | 20.15 | 1.46 | 60.56 | 13.84 | 0.00 | 0.00 |
| oxytocin | 2.76 | 2.10 | 60.94 | 1.31 | 0.20 | 1.00 |
| social treatment | 2.09 | 2.06 | 60.97 | 1.01 | 0.32 | 1.00 |
| time | -0.85 | 0.35 | 136.16 | -2.42 | 0.02 | 0.15 |
| oxytocin×social treatment | -4.76 | 2.95 | 61.29 | -1.61 | 0.11 | 1.00 |
| oxytocin×time | -0.21 | 0.52 | 137.39 | -0.40 | 0.69 | 1.00 |
| social treatment×time | -0.64 | 0.51 | 136.79 | -1.26 | 0.21 | 1.00 |
| oxytocin×social treatment×time | 1.33 | 0.74 | 137.61 | 1.81 | 0.07 | 0.66 |

social skills training

24

supportive psychotherapy

oxytocin

N (PANSS)

21

placebo oxytocin

18

15

0 1 2 3 0 1 2 3

week

Figure 1.3.3 The predicted values of the full interaction model with N (PANSS) as the outcome

## The full interaction model with G (PANSS) as the outcome

Table 1.3.4. The full interaction model with G (PANSS) as the outcome, N = 51

|  | Estimate | Std. Error | df | t value | p-value | q-value |
| --- | --- | --- | --- | --- | --- | --- |
| (Intercept) | 30.16 | 2.00 | 58.05 | 15.09 | 0.00 | 0 |
| oxytocin | 4.20 | 2.89 | 58.37 | 1.45 | 0.15 | 1 |
| social treatment | 1.66 | 2.83 | 58.39 | 0.59 | 0.56 | 1 |
| time | -0.72 | 0.45 | 135.79 | -1.60 | 0.11 | 1 |
| oxytocin×social treatment | -3.14 | 4.05 | 58.67 | -0.77 | 0.44 | 1 |
| oxytocin×time | 0.08 | 0.67 | 136.90 | 0.11 | 0.91 | 1 |
| social treatment×time | -0.06 | 0.65 | 136.34 | -0.09 | 0.93 | 1 |
| oxytocin×social treatment×time | -0.64 | 0.94 | 137.09 | -0.68 | 0.50 | 1 |

social skills training

36

supportive psychotherapy

oxytocin

G (PANSS)

32

placebo oxytocin

28

24

0 1 2 3 0 1 2 3

week

Figure 1.3.4 The predicted values of the full interaction model with G (PANSS) as the outcome

## The full interaction model with Withdrawal (PANSS) as the outcome

Table 1.3.5. The full interaction model with Withdrawal (PANSS) as the outcome, N = 51

|  | Estimate | Std. Error | df | t value | p-value | q-value |
| --- | --- | --- | --- | --- | --- | --- |
| (Intercept) | 3.15 | 0.23 | 62.64 | 13.76 | 0.00 | 0.00 |
| oxytocin | 0.35 | 0.33 | 63.05 | 1.06 | 0.29 | 1.00 |
| social treatment | 0.33 | 0.32 | 63.09 | 1.01 | 0.32 | 1.00 |
| time | -0.13 | 0.06 | 136.57 | -2.24 | 0.03 | 0.24 |
| oxytocin×social treatment | -0.75 | 0.46 | 63.45 | -1.61 | 0.11 | 1.00 |
| oxytocin×time | -0.04 | 0.09 | 137.87 | -0.52 | 0.61 | 1.00 |
| social treatment×time | -0.12 | 0.08 | 137.26 | -1.49 | 0.14 | 1.00 |
| oxytocin×social treatment×time | 0.23 | 0.12 | 138.12 | 1.91 | 0.06 | 0.52 |

social skills training

4.0

supportive psychotherapy

3.5

Withdrawal (PANSS)

oxytocin

placebo oxytocin

3.0

2.5

0 1 2 3 0 1 2 3

time

Figure 1.3.5 The predicted values of the full interaction model with Withdrawal (PANSS) as the outcome

## The full interaction model with Delusion (PANSS) as the outcome

Table 1.3.6. The full interaction model with Delusion (PANSS) as the outcome, N = 51

|  | Estimate | Std. Error | df | t value | p-value | q-value |
| --- | --- | --- | --- | --- | --- | --- |
| (Intercept) | 1.70 | 0.23 | 50.85 | 7.53 | 0.00 | 0.00 |
| oxytocin | 0.40 | 0.33 | 51.04 | 1.22 | 0.23 | 1.00 |
| social treatment | -0.16 | 0.32 | 51.05 | -0.51 | 0.61 | 1.00 |
| time | -0.12 | 0.04 | 133.66 | -2.93 | 0.00 | 0.04 |
| oxytocin×social treatment | 0.10 | 0.46 | 51.21 | 0.21 | 0.83 | 1.00 |
| oxytocin×time | 0.09 | 0.06 | 134.41 | 1.61 | 0.11 | 0.99 |
| social treatment×time | 0.02 | 0.06 | 134.00 | 0.40 | 0.69 | 1.00 |
| oxytocin×social treatment×time | -0.04 | 0.08 | 134.52 | -0.53 | 0.60 | 1.00 |

social skills training

2.4

supportive psychotherapy

2.0

Delusion (PANSS)

1.6

oxytocin

placebo oxytocin

1.2

0.8

0 1 2 3

time

0 1 2 3

Figure 1.3.6 The predicted values of the full interaction model with Delusion (PANSS) as the outcome

## The full interaction model with Anxiety (PANSS) as the outcome

Table 1.3.7. The full interaction model with Anxiety (PANSS) as the outcome, N = 51

|  | Estimate | Std. Error | df | t value | p-value | q-value |
| --- | --- | --- | --- | --- | --- | --- |
| (Intercept) | 2.52 | 0.24 | 67.28 | 10.50 | 0.00 | 0.00 |
| oxytocin | 0.54 | 0.35 | 67.79 | 1.57 | 0.12 | 1.00 |
| social treatment | -0.08 | 0.34 | 67.83 | -0.24 | 0.81 | 1.00 |
| time | -0.13 | 0.07 | 137.20 | -1.88 | 0.06 | 0.56 |
| oxytocin×social treatment | -0.47 | 0.49 | 68.27 | -0.97 | 0.33 | 1.00 |
| oxytocin×time | -0.02 | 0.10 | 138.66 | -0.23 | 0.82 | 1.00 |
| social treatment×time | 0.00 | 0.10 | 138.03 | 0.00 | 1.00 | 1.00 |
| oxytocin×social treatment×time | -0.09 | 0.14 | 138.97 | -0.63 | 0.53 | 1.00 |

social skills training

3

supportive psychotherapy

oxytocin

Anxiety (PANSS)

placebo oxytocin

2

0 1 2 3 0 1 2 3

time

Figure 1.3.7 The predicted values of the full interaction model with Anxiety (PANSS) as the outcome

## The full interaction model with Disorganization (PANSS) as the outcome

Table 1.3.8. The full interaction model with Disorganization (PANSS) as the outcome, N = 51

supportive psychotherapy

|  | Estimate | Std. Error | df | t value | p-value | q-value |
| --- | --- | --- | --- | --- | --- | --- |
| (Intercept) | 1.96 | 0.16 | 61.24 | 11.88 | 0.00 | 0 |
| oxytocin | -0.10 | 0.24 | 61.70 | -0.44 | 0.66 | 1 |
| social treatment | 0.11 | 0.23 | 61.74 | 0.48 | 0.63 | 1 |
| time | -0.04 | 0.04 | 133.37 | -0.95 | 0.34 | 1 |
| oxytocin×social treatment | 0.17 | 0.33 | 62.13 | 0.51 | 0.61 | 1 |
| oxytocin×time | 0.00 | 0.07 | 134.84 | -0.07 | 0.95 | 1 |
| social treatment×time | 0.03 | 0.06 | 134.18 | 0.51 | 0.61 | 1 |
| oxytocin×social treatment×time | -0.06 | 0.09 | 135.14 | -0.60 | 0.55 | 1 |

social skills training

2.50

2.25

2.00

Disorganization (PANSS)

1.75

oxytocin

placebo oxytocin

1.50

0 1 2 3 0 1 2 3

time

Figure 1.3.8 The predicted values of the full interaction model with Disorganization (PANSS) as the outcome

## The full interaction model with Poor control (PANSS) as the outcome

Table 1.3.9. The full interaction model with Poor control (PANSS) as the outcome, N = 51

|  | Estimate | Std. Error | df | t value | p-value | q-value |
| --- | --- | --- | --- | --- | --- | --- |
| (Intercept) | 1.46 | 0.13 | 67.40 | 11.01 | 0.00 | 0 |
| oxytocin | 0.21 | 0.19 | 67.97 | 1.10 | 0.28 | 1 |
| social treatment | 0.18 | 0.19 | 68.01 | 0.97 | 0.34 | 1 |
| time | -0.03 | 0.04 | 134.70 | -0.78 | 0.44 | 1 |
| oxytocin×social treatment | -0.42 | 0.27 | 68.50 | -1.54 | 0.13 | 1 |
| oxytocin×time | 0.01 | 0.06 | 136.32 | 0.20 | 0.84 | 1 |
| social treatment×time | -0.03 | 0.06 | 135.66 | -0.46 | 0.64 | 1 |
| oxytocin×social treatment×time | 0.00 | 0.08 | 136.68 | -0.01 | 0.99 | 1 |

social skills training

1.8

supportive psychotherapy

1.5

Poor control (PANSS)

oxytocin

placebo oxytocin

1.2

0.9

0 1 2 3

time

0 1 2 3

Figure 1.3.9 The predicted values of the full interaction model with Poor control (PANSS) as the outcome

# The oxytocin main effect models

To estimate the effects over time of oxytocin (without social treatments), mixed effects mo dels were used for each of the five social i nteraction f actors, t he i ndividual s ocial interaction s cores, a nd t he PANSS s cores as the outcome. In these models, multiple measurements (one per time point) for each patient were modeled as random effects. T he c oefficients of int erest are the cha nge in the effe cts of o xyt ocin over time , which is represented as the time interaction. I.e., oxytocin × time. The p-values due to multiple testing were adjusted using Bonferroni corrrection for each group of outcomes (the set of social interaction factors, the set of the individual social interaction scores, and the set of PANSS-related scores) and the corrected values are referred to as the ‘q-values.’

# The oxytocin main effect m odels: t he fi ve so cial interaction factors as the outcomes

This section provides the results for each of the five social interaction factors—initiation, synchrony, positive affects, tension, a nd withdrawal—as t he o utcome in t he models.

## The oxytocin main effect model with Factor 1 (synchrony) total as the outcome

Table 2.1.1. The oxytocin main effect model with Factor 1 (synchrony) total as the outcome, N = 43

|  | Estimate | Std. Error | df | t value | p-value | q-value |
| --- | --- | --- | --- | --- | --- | --- |
|  | (Intercept) -0.06 | 0.21 | 60.83 | -0.27 | 0.79 | 1 |
|  | oxytocin -0.08 | 0.31 | 60.83 | -0.26 | 0.79 | 1 |
|  | time 0.05 | 0.06 | 41.00 | 0.82 | 0.42 | 1 |
|  | oxytocin×time 0.04 | 0.09 | 41.00 | 0.41 | 0.68 | 1 |
| 0.6 |  |  |  |  |  |  |

0.3

Factor 1 (synchrony) total

oxytocin

0.0 placebo

oxytocin

−0.3

−0.6

0 1 2 3

time

Figure 2.1.1 The predicted values of the oxytocin main effect model with Factor 1 (synchrony) total as the outcome

## The oxytocin main effect model with Factor 2 (initiation) total as the outcome

Table 2.1.2. The oxytocin main effect model with Factor 2 (initiation) total as the outcome, N = 43

|  | Estimate | Std. Error | df | t value | p-value | q-value |
| --- | --- | --- | --- | --- | --- | --- |
|  | (Intercept) 0.11 | 0.21 | 56.17 | 0.51 | 0.61 | 1 |
|  | oxytocin -0.27 | 0.31 | 56.17 | -0.87 | 0.39 | 1 |
|  | time -0.04 | 0.06 | 41.00 | -0.73 | 0.47 | 1 |
|  | oxytocin×time 0.13 | 0.08 | 41.00 | 1.59 | 0.12 | 1 |
| 0.6 |  |  |  |  |  |  |

0.3

0.0

Factor 2 (initiation) total

oxytocin

placebo oxytocin

−0.3

−0.6

0 1 2 3

time

Figure 2.1.2 The predicted values of the oxytocin main effect model with Factor 2 (initiation) total as the outcome

## 2.1.3 The oxytocin main effect model with Factor 3 (tension) total as the outcome

Table 2.1.3. The oxytocin main effect model with Factor 3 (tension) total as the outcome, N = 43

|  | Estimate | Std. Error | df | t value | p-value | q-value |
| --- | --- | --- | --- | --- | --- | --- |
|  | (Intercept) -0.04 | 0.20 | 62.24 | -0.19 | 0.85 | 1 |
|  | oxytocin 0.40 | 0.30 | 62.24 | 1.30 | 0.20 | 1 |
|  | time -0.01 | 0.06 | 41.00 | -0.09 | 0.92 | 1 |
|  | oxytocin×time -0.18 | 0.09 | 41.00 | -1.85 | 0.07 | 1 |
| 0.8 |  |  |  |  |  |  |

0.4

Factor 3 (tension) total

0.0

oxytocin

placebo oxytocin

−0.4

0 1 2 3

time

Figure 2.1.3 The predicted values of the oxytocin main effect model with Factor 3 (tension) total as the outcome

## 2.1.4 The oxytocin main effect model with Factor 4 (withdrawal) total as the outcome

Table 2.1.4. The oxytocin main effect model with Factor 4 (withdrawal) total as the outcome, N = 43

|  | Estimate | Std. Error | df | t value | p-value | q-value |
| --- | --- | --- | --- | --- | --- | --- |
| (Intercept) | 0.10 | 0.20 | 63.99 | 0.49 | 0.63 | 1 |
| oxytocin | -0.19 | 0.31 | 63.99 | -0.63 | 0.53 | 1 |
| time | -0.05 | 0.07 | 41.00 | -0.78 | 0.44 | 1 |
| oxytocin×time | 0.04 | 0.10 | 41.00 | 0.44 | 0.66 | 1 |

0.3

0.0

Factor 4 (withdrawal) total

oxytocin

placebo oxytocin

−0.3

−0.6

0 1 2 3

time

Figure 2.1.4 The predicted values of the oxytocin main effect model with Factor 4 (withdrawal) total as the outcome

## The oxytocin main effect model with Factor 5 (Positive affect) total as the outcome

Table 2.1.5. The oxytocin main effect model with Factor 5 (Positive affect) total as the outcome, N = 43

|  | Estimate | Std. Error | df | t value | p-value |  | q-value |
| --- | --- | --- | --- | --- | --- | --- | --- |
|  | (Intercept) 0.06 | 0.19 | 57.16 | 0.29 | 0.77 |  | 1 |
|  | oxytocin -0.47 | 0.29 | 57.16 | -1.61 | 0.11 |  | 1 |
|  | time 0.02 | 0.05 | 41.00 | 0.39 | 0.70 |  | 1 |
|  | oxytocin×time 0.10 | 0.08 | 41.00 | 1.31 | 0.20 |  | 1 |
| 0.5 |  |  |  |  |  |  |  |
|  |  |  | | | |  |  |
| 0.0 |  |  | | | |  |  |

oxytocin

placebo oxytocin

Factor 5 (Positive affect) total

−0.5

0 1 2 3

time

Figure 2.1.5 The predicted values of the oxytocin main effect model with Factor 5 (Positive affect) total as the outcome

## The oxytocin main effect model with Factor 1 (synchrony) Conflict Inter- action as the outcome

Table 2.1.6. The oxytocin main effect model with Factor 1 (synchrony) Conflict Interaction as the outcome, N = 43

|  | Estimate | Std. Error | df | t value | p-value | q-value |
| --- | --- | --- | --- | --- | --- | --- |
|  | (Intercept) -0.08 | 0.21 | 63.96 | -0.37 | 0.71 | 1 |
|  | oxytocin -0.09 | 0.31 | 63.96 | -0.27 | 0.79 | 1 |
|  | time 0.04 | 0.07 | 41.00 | 0.63 | 0.53 | 1 |
|  | oxytocin×time 0.05 | 0.10 | 41.00 | 0.50 | 0.62 | 1 |
| 0.6 |  |  |  |  |  |  |

0.3

Factor 1 (synchrony) Conflict Interaction

0.0

oxytocin

placebo oxytocin

−0.3

−0.6

0 1 2 3

time

Figure 2.1.6 The predicted values of the oxytocin main effect model with CIB total as the outcome

## 2.1.7 The oxytocin main effect model with Factor 2 (initiation) Conflict Interac- tion as the outcome

Table 2.1.7. The oxytocin main effect model with Factor 2 (initiation) Conflict Interaction as the outcome, N = 43

|  | Estimate | Std. Error | df | t value | p-value | q-value |
| --- | --- | --- | --- | --- | --- | --- |
| (Intercept) | 0.02 | 0.21 | 61.74 | 0.08 | 0.94 | 1 |
| oxytocin | -0.22 | 0.31 | 61.74 | -0.70 | 0.48 | 1 |
| time | -0.02 | 0.06 | 41.00 | -0.38 | 0.71 | 1 |
| oxytocin×time | 0.14 | 0.10 | 41.00 | 1.48 | 0.15 | 1 |

0.4

Factor 2 (initiation) Conflict Interaction

0.0

oxytocin

placebo oxytocin

−0.4

0 1 2 3

time

Figure 2.1.7 The predicted values of the oxytocin main effect model with Factor 1 (synchrony) Conflict Interaction as the outcome

## 2.1.8 The oxytocin main effect model with Factor 3 (tension) Conflict Interaction as the outcome

Table 2.1.8. The oxytocin main effect model with Factor 3 (tension) Conflict Interaction as the outcome, N

= 43

|  | Estimate | Std. Error | df | t value | p-value | q-value |
| --- | --- | --- | --- | --- | --- | --- |
| (Intercept) | 0.06 | 0.21 | 71.54 | 0.28 | 0.78 | 1 |
| oxytocin | 0.16 | 0.31 | 71.54 | 0.50 | 0.62 | 1 |
| time | -0.03 | 0.08 | 41.00 | -0.36 | 0.72 | 1 |
| oxytocin×time | -0.10 | 0.12 | 41.00 | -0.88 | 0.38 | 1 |

0.4

Factor 3 (tension) Conflict Interaction

0.0

oxytocin

placebo oxytocin

−0.4

0 1 2 3

time

Figure 2.1.8 The predicted values of the oxytocin main effect model with Factor 2 (initiation) Conflict Interaction as the outcome

## The oxytocin main effect model with Factor 4 (withdrawal) Conflict Inter- action as the outcome

Table 2.1.9. The oxytocin main effect model with Factor 4 (withdrawal) Conflict Interaction as the outcome, N = 43

|  | Estimate | Std. Error | df | t value | p-value | q-value |
| --- | --- | --- | --- | --- | --- | --- |
| (Intercept) | 0.01 | 0.21 | 63.95 | 0.04 | 0.96 | 1 |
| oxytocin | -0.04 | 0.31 | 63.95 | -0.13 | 0.90 | 1 |
| time | -0.02 | 0.07 | 41.00 | -0.31 | 0.76 | 1 |
| oxytocin×time | 0.03 | 0.10 | 41.00 | 0.26 | 0.79 | 1 |

0.25

Factor 4 (withdrawal) Conflict Interaction

0.00

oxytocin

placebo oxytocin

−0.25

−0.50

0 1 2 3

time

Figure 2.1.9 The predicted values of the oxytocin main effect model with Factor 3 (tension) Conflict Inter- action as the outcome

## The oxytocin main effect model with Factor 5 (Positive affect) Conflict Interaction as the outcome

Table 2.1.10. The oxytocin main effect model with Factor 5 (Positive affect) Conflict Interaction as the outcome, N = 43

|  | Estimate | Std. Error | df | t value | p-value | q-value |
| --- | --- | --- | --- | --- | --- | --- |
| (Intercept) | -0.10 | 0.20 | 57.28 | -0.50 | 0.62 | 1.00 |
| oxytocin | -0.19 | 0.30 | 57.28 | -0.66 | 0.51 | 1.00 |
| time | 0.13 | 0.05 | 41.00 | 2.41 | 0.02 | 0.43 |
| oxytocin×time | -0.08 | 0.08 | 41.00 | -1.03 | 0.31 | 1.00 |

0.4

Factor 5 (Positive affect) Conflict Interaction

0.0

oxytocin

placebo oxytocin

−0.4

−0.8

0 1 2 3

time

Figure 2.1.10 The predicted values of the oxytocin main effect model with Factor 4 (withdrawal) Conflict Interaction as the outcome

## The oxytocin main effect model with Factor 1 (synchrony) Positive Inter- action as the outcome

Table 2.1.11. The oxytocin main effect model with Factor 1 (synchrony) Positive Interaction as the outcome, N = 43

|  | Estimate | Std. Error | df | t value | p-value | q-value |
| --- | --- | --- | --- | --- | --- | --- |
| (Intercept) | -0.03 | 0.20 | 74.01 | -0.13 | 0.90 | 1 |
| oxytocin | -0.14 | 0.31 | 74.01 | -0.47 | 0.64 | 1 |
| time | 0.08 | 0.08 | 41.00 | 0.98 | 0.33 | 1 |
| oxytocin×time | 0.00 | 0.12 | 41.00 | 0.04 | 0.97 | 1 |

0.4

Factor 1 (synchrony) Positive Interaction

oxytocin

0.0 placebo

oxytocin

−0.4

0 1 2 3

time

Figure 2.1.11 The predicted values of the oxytocin main effect model with Factor 5 (Positive affect) Conflict Interaction as the outcome

## The oxytocin main effect model with Factor 2 (initiation) Positive Inter- action as the outcome

Table 2.1.12. The oxytocin main effect model with Factor 2 (initiation) Positive Interaction as the outcome, N = 43

|  | Estimate | Std. Error | df | t value | p-value | q-value |
| --- | --- | --- | --- | --- | --- | --- |
|  | (Intercept) 0.04 | 0.21 | 66.75 | 0.20 | 0.84 | 1 |
|  | oxytocin -0.08 | 0.31 | 66.75 | -0.27 | 0.79 | 1 |
|  | time -0.01 | 0.07 | 41.00 | -0.12 | 0.91 | 1 |
|  | oxytocin×time 0.05 | 0.11 | 41.00 | 0.50 | 0.62 | 1 |
| 0.6 |  |  |  |  |  |  |

0.3

Factor 2 (initiation) Positive Interaction

0.0

oxytocin

placebo oxytocin

−0.3

0 1 2 3

time

Figure 2.1.12 The predicted values of the oxytocin main effect model with Factor 1 (synchrony) Positive Interaction as the outcome

## The oxytocin main effect model with Factor 3 (tension) Positive Interac- tion as the outcome

Table 2.1.13. The oxytocin main effect model with Factor 3 (tension) Positive Interaction as the outcome, N = 43

Estimate Std. Error df t value p-value q-value

(Intercept) -0.09 0.20 66.74 -0.43 0.67 1

oxytocin 0.36 0.31 66.74 1.16 0.25 1

time 0.02 0.07 41.00 0.23 0.82 1

oxytocin×time -0.12 0.10 41.00 -1.19 0.24 1

0.75

0.50

Factor 3 (tension) Positive Interaction

0.25

0.00

oxytocin

placebo oxytocin

−0.25

−0.50

0 1 2 3

time

Figure 2.1.13 The predicted values of the oxytocin main effect model with Factor 2 (initiation) Positive Interaction as the outcome

## The oxytocin main effect model with Factor 4 (withdrawal) Positive In- teraction as the outcome

Table 2.1.14. The oxytocin main effect model with Factor 4 (withdrawal) Positive Interaction as the outcome, N = 43

|  | Estimate | Std. Error | df | t value | p-value |  | q-value |
| --- | --- | --- | --- | --- | --- | --- | --- |
|  | (Intercept) 0.03 | 0.20 | 79.6 | 0.13 | 0.90 |  | 1 |
|  | oxytocin -0.26 | 0.31 | 79.6 | -0.84 | 0.40 |  | 1 |
|  | time 0.01 | 0.09 | 41.0 | 0.13 | 0.90 |  | 1 |
|  | oxytocin×time 0.06 | 0.13 | 41.0 | 0.43 | 0.67 |  | 1 |
| 0.50 |  |  |  |  |  |  |  |
|  |  |  |  |  |  |  |  |
| 0.25 |  |  |  |  |  |  |  |

0.00

Factor 4 (withdrawal) Positive Interaction

−0.25

oxytocin

placebo oxytocin

−0.50

0 1 2 3

time

Figure 2.1.14 The predicted values of the oxytocin main effect model with Factor 3 (tension) Positive Inter- action as the outcome

## The oxytocin main effect model with Factor 5 (Positive affect) Positive Interaction as the outcome

Table 2.1.15. The oxytocin main effect model with Factor 5 (Positive affect) Positive Interaction as the outcome, N = 43

|  | Estimate | Std. Error | df | t value | p-value | q-value |
| --- | --- | --- | --- | --- | --- | --- |
| (Intercept) | 0.19 | 0.19 | 64.66 | 0.97 | 0.33 | 1 |
| oxytocin | -0.53 | 0.29 | 64.66 | -1.84 | 0.07 | 1 |
| time | -0.05 | 0.06 | 41.00 | -0.84 | 0.40 | 1 |
| oxytocin×time | 0.13 | 0.10 | 41.00 | 1.37 | 0.18 | 1 |

0.4

Factor 5 (Positive affect) Positive Interaction

0.0 oxytocin

placebo oxytocin

−0.4

−0.8

0 1 2 3

time

Figure 2.1.15 The predicted values of the oxytocin main effect model with Factor 4 (withdrawal) Positive Interaction as the outcome

## The oxytocin main effect model with Factor 1 (synchrony) Supportive

**interaction as the outcome**

Table 2.1.16. The oxytocin main effect m odel w ith F actor 1 ( synchrony) S upportive I nteracton a s the outcome, N = 43

|  | Estimate | Std. Error | df | t value | p-value | q-value |
| --- | --- | --- | --- | --- | --- | --- |
| (Intercept) | 0.18 | 0.21 | 76.85 | 0.88 | 0.38 | 1 |
| oxytocin | -0.42 | 0.31 | 76.85 | -1.34 | 0.18 | 1 |
| time | -0.08 | 0.08 | 41.00 | -0.89 | 0.38 | 1 |
| oxytocin×time | 0.16 | 0.13 | 41.00 | 1.30 | 0.20 | 1 |

0.4

Factor 1 (synchrony) Supportive interaction

0.0

oxytocin

placebo oxytocin

−0.4

0 1 2 3

time

Figure 2.1.16 The predicted values of the oxytocin main effect model with Factor 5 (Positive affect) Positive Interaction as the outcome

## 2.1.17 The oxytocin main effect model with Factor 2 (initiation) Supportive

**interaction as the outcome**

Table 2.1.17. The oxytocin main effect model with Factor 2 (initiation) Supportive interaction as the outcome, N = 43

|  | Estimate | Std. Error | df | t value | p-value | q-value |
| --- | --- | --- | --- | --- | --- | --- |
| (Intercept) | 0.22 | 0.21 | 60.58 | 1.09 | 0.28 | 1 |
| oxytocin | -0.27 | 0.31 | 60.58 | -0.88 | 0.38 | 1 |
| time | -0.09 | 0.06 | 41.00 | -1.45 | 0.15 | 1 |
| oxytocin×time | 0.09 | 0.09 | 41.00 | 0.93 | 0.36 | 1 |

0.6

0.3

Factor 2 (initiation) Supportive interaction

0.0

oxytocin

placebo oxytocin

−0.3

0 1 2 3

time

Figure 2.1.17 The predicted values of the oxytocin main effect model with Factor 1 (synchrony) Supportive

interaction as the outcome

## 2.1.18 The oxytocin main effect model with Factor 3 (EmpathEmoEx) Supportive

**interaction as the outcome**

Table 2.1.18. The oxytocin main effect model with Factor 3 (EmpathEmoEx) Supportive interaction as the outcome, N = 43

|  | Estimate | Std. Error | df | t value | p-value | q-value |
| --- | --- | --- | --- | --- | --- | --- |
| (Intercept) | 0.22 | 0.20 | 82 | 1.08 | 0.28 | 1 |
| oxytocin | 0.00 | 0.31 | 82 | 0.00 | 1.00 | 1 |
| time | -0.14 | 0.10 | 82 | -1.41 | 0.16 | 1 |
| oxytocin×time | -0.03 | 0.15 | 82 | -0.20 | 0.84 | 1 |

0.4

Factor 3 (EmpathEmoEx) Supportive interaction

0.0

oxytocin

placebo oxytocin

−0.4

−0.8

0 1 2 3

time

Figure 2.1.18 The predicted values of the oxytocin main effect model with Factor 2 (initiation) Supportive

interaction as the outcome

## 2.1.19 The oxytocin main effect model with Factor 4 (Positive affect) Supportive

**interaction as the outcome**

Table 2.1.19. The oxytocin main effect m odel w ith Factor 4 ( Positive a ffect) Su pportive In teracton as the outcome, N = 43

|  | Estimate | Std. Error | df | t value | p-value | q-value |
| --- | --- | --- | --- | --- | --- | --- |
| (Intercept) | 0.03 | 0.19 | 71.4 | 0.15 | 0.88 | 1 |
| oxytocin | -0.43 | 0.29 | 71.4 | -1.48 | 0.14 | 1 |
| time | 0.08 | 0.07 | 41.0 | 1.07 | 0.29 | 1 |
| oxytocin×time | 0.00 | 0.11 | 41.0 | 0.04 | 0.97 | 1 |

0.4

Factor 4 (Positive affect) Supportive interaction

0.0

oxytocin

placebo oxytocin

−0.4

−0.8

0 1 2 3

time

Figure 2.1.19 The predicted values of the oxytocin main effect model with Factor 3 (EmpathEmoEx) Sup-portive interaction as the outcome

## 2.1.20 The oxytocin main effect model with Factor 5 (Critisism) Supportive

**interaction as the outcome**

Table 2.1.20. The oxytocin main effect model with Factor 5 (Critisism) Supportive interaction as the outcome, N = 43

|  | Estimate | Std. Error | df | t value | p-value | q-value |
| --- | --- | --- | --- | --- | --- | --- |
| (Intercept) | -0.39 | 0.20 | 80.34 | -1.94 | 0.06 | 1.00 |
| oxytocin | 0.81 | 0.30 | 80.34 | 2.70 | 0.01 | 0.18 |
| time | 0.14 | 0.09 | 41.00 | 1.58 | 0.12 | 1.00 |
| oxytocin×time | -0.24 | 0.13 | 41.00 | -1.84 | 0.07 | 1.00 |

0.5

Factor 5 (Critisism) Supportive interaction

0.0

oxytocin

placebo oxytocin

−0.5

0 1 2 3

time

Figure 2.1.20 The predicted values of the oxytocin main effect model with Factor 4 (Positive affect) Supportive

interaction as the outcome

## 2.1.21 The oxytocin main effect model with CIB total as the outcome

Table 2.1.21. The oxytocin main effect model with CIB total as the outcome, N = 43

|  | Estimate | Std. Error | df | t value | p-value |  | q-value |
| --- | --- | --- | --- | --- | --- | --- | --- |
|  | (Intercept) 20.26 | 1.90 | 52.74 | 10.68 | 0.00 |  | 0 |
|  | oxytocin -2.09 | 2.85 | 52.74 | -0.73 | 0.47 |  | 1 |
|  | time 0.19 | 0.45 | 41.00 | 0.41 | 0.68 |  | 1 |
|  | oxytocin×time 0.59 | 0.68 | 41.00 | 0.87 | 0.39 |  | 1 |
| 25.0 |  |  |  |  |  |  |  |
|  |  |  |  |  |  |  |  |
|  |  |  |  |  |  |  |  |
| 22.5  20.0  17.5 |  |  | | | |  |  |

oxytocin

CIB total

placebo oxytocin

15.0

0 1 2 3

time

Figure 2.1.21 The predicted values of the oxytocin main effect model with Factor 5 (Critisism) Supportive

interaction as the outcome

# The oxytocin main effect models: the individual social interac- tion scores as the outcomes

This section provides the results for each of the individual social interaction scores as the outcome in the models.

## The oxytocin main effect model with Acknowledgment as the outcome

Table 2.2.1. The oxytocin main effect model with Acknowledgment as the outcome, N = 43

|  | Estimate | Std. Error | df | t value | p-value | q-value |
| --- | --- | --- | --- | --- | --- | --- |
| (Intercept) | 4.07 | 0.18 | 61.35 | 22.73 | 0.00 | 0 |
| oxytocin | -0.27 | 0.27 | 61.35 | -1.01 | 0.32 | 1 |
| time | 0.01 | 0.05 | 41.00 | 0.21 | 0.83 | 1 |
| oxytocin×time | 0.08 | 0.08 | 41.00 | 1.03 | 0.31 | 1 |

4.5

4.2

3.9

Acknowledgment

oxytocin

placebo oxytocin

3.6

0 1 2 3

time

Figure 2.2.1 The predicted values of the oxytocin main effect model with Acknowledgment as the outcome

## The oxytocin main effect model with Alert as the outcome

Table 2.2.2. The oxytocin main effect model with Alert as the outcome, N = 43

|  | Estimate | Std. Error | df | t value | p-value | q-value |
| --- | --- | --- | --- | --- | --- | --- |
| (Intercept) | 2.83 | 0.23 | 52.93 | 12.08 | 0.00 | 0 |
| oxytocin | 0.07 | 0.35 | 52.93 | 0.19 | 0.85 | 1 |
| time | 0.04 | 0.06 | 41.00 | 0.78 | 0.44 | 1 |
| oxytocin×time | -0.01 | 0.08 | 41.00 | -0.11 | 0.92 | 1 |

3.3

3.0

Alert

oxytocin

placebo oxytocin

2.7

2.4

0 1 2 3

time

Figure 2.2.2 The predicted values of the oxytocin main effect model with Alert as the outcome

## The oxytocin main effect model with Anger as the outcome

Table 2.2.3. The oxytocin main effect model with Anger as the outcome, N = 43 Estimate Std. Error df t value p-value q-value

(Intercept) 1.03 0.04 81.38 25.77 0.00 0

oxytocin 0.11 0.06 81.38 1.75 0.08 1

time 0.02 0.02 41.00 1.02 0.31 1

oxytocin×time -0.02 0.03 41.00 -0.57 0.57 1

1.25

1.20

1.15

1.10

Anger

oxytocin

placebo oxytocin

1.05

1.00

0.95

0 1 2 3

time

Figure 2.2.3 The predicted values of the oxytocin main effect model with Anger as the outcome

## The oxytocin main effect model with Anxiety as the outcome

Table 2.2.4. The oxytocin main effect model with Anxiety as the outcome, N = 43

|  | Estimate | Std. Error | df | t value | p-value | q-value |
| --- | --- | --- | --- | --- | --- | --- |
| (Intercept) | 1.49 | 0.10 | 56.75 | 14.25 | 0.00 | 0 |
| oxytocin | -0.13 | 0.16 | 56.75 | -0.81 | 0.42 | 1 |
| time | 0.01 | 0.03 | 41.00 | 0.41 | 0.69 | 1 |
| oxytocin×time | -0.03 | 0.04 | 41.00 | -0.82 | 0.42 | 1 |

1.6

1.4

Anxiety

oxytocin

placebo oxytocin

1.2

0 1 2 3

time

Figure 2.2.4 The predicted values of the oxytocin main effect model with Anxiety as the outcome

## The oxytocin main effect model with Avoidance as the outcome

Table 2.2.5. The oxytocin main effect model with Avoidance as the outcome, N = 43

|  | | Estimate | Std. Error | df | t value | p-value | |  | q-value |
| --- | --- | --- | --- | --- | --- | --- | --- | --- | --- |
|  |  | (Intercept) 1.08 | 0.05 | 67.47 | 20.92 | 0.00 | |  | 0 |
|  |  | oxytocin 0.07 | 0.08 | 67.47 | 0.84 | 0.40 | |  | 1 |
|  |  | time 0.01 | 0.02 | 41.00 | 0.52 | 0.61 | |  | 1 |
|  |  | oxytocin×time 0.00 | 0.03 | 41.00 | -0.02 | 0.99 | |  | 1 |
| 1.3 |  |  |  |  |  |  |  |  |  |
|  |  |  |  |  |  |  |  |  |  |
|  |  |  |  | | | | |  |  |
| 1.2  1.1 |  |  |  | | | | |  |  |

oxytocin

Avoidance

placebo oxytocin

1.0

0 1 2 3

time

Figure 2.2.5 The predicted values of the oxytocin main effect model with Avoidance as the outcome

## The oxytocin main effect model with Blunted affect as the outcome

Table 2.2.6. The oxytocin main effect model with Blunted affect as the outcome, N = 43

|  | Estimate | Std. Error | df | t value | p-value | q-value |
| --- | --- | --- | --- | --- | --- | --- |
| (Intercept) | 2.04 | 0.16 | 49.05 | 12.84 | 0.00 | 0 |
| oxytocin | -0.08 | 0.24 | 49.05 | -0.32 | 0.75 | 1 |
| time | -0.01 | 0.03 | 41.00 | -0.22 | 0.83 | 1 |
| oxytocin×time | -0.03 | 0.05 | 41.00 | -0.53 | 0.60 | 1 |

2.4

2.2

2.0 oxytocin

Blunted affect

placebo oxytocin

1.8

1.6

0 1 2 3

time

Figure 2.2.6 The predicted values of the oxytocin main effect model with Blunted affect as the outcome

## The oxytocin main effect model with Constricted as the outcome

Table 2.2.7. The oxytocin main effect model with Constricted as the outcome, N = 43

|  | Estimate | Std. Error | df | t value | p-value | q-value |
| --- | --- | --- | --- | --- | --- | --- |
| (Intercept) | 1.98 | 0.15 | 56.53 | 13.01 | 0.00 | 0 |
| oxytocin | 0.11 | 0.23 | 56.53 | 0.47 | 0.64 | 1 |
| time | 0.00 | 0.04 | 41.00 | -0.06 | 0.96 | 1 |
| oxytocin×time | -0.07 | 0.06 | 41.00 | -1.14 | 0.26 | 1 |

2.25

2.00

Constricted

oxytocin

placebo oxytocin

1.75

1.50

0 1 2 3

time

Figure 2.2.7 The predicted values of the oxytocin main effect model with Constricted as the outcome

## 2.2.8 The oxytocin main effect model with Criticism as the outcome

Table 2.2.8. The oxytocin main effect model with Criticism as the outcome, N = 43

|  | Estimate | Std. Error | df | t value | p-value | q-value |
| --- | --- | --- | --- | --- | --- | --- |
| (Intercept) | 1.08 | 0.05 | 81.74 | 19.98 | 0.00 | 0 |
| oxytocin | 0.12 | 0.08 | 81.74 | 1.45 | 0.15 | 1 |
| time | 0.03 | 0.02 | 41.00 | 1.40 | 0.17 | 1 |
| oxytocin×time | -0.04 | 0.04 | 41.00 | -1.01 | 0.32 | 1 |

1.3

1.2

oxytocin

Criticism

placebo oxytocin

1.1

1.0

0 1 2 3

time

Figure 2.2.8 The predicted values of the oxytocin main effect model with Criticism as the outcome

## The oxytocin main effect model with Detachment as the outcome

Table 2.2.9. The oxytocin main effect model with Detachment as the outcome, N = 43

|  | Estimate | Std. Error | df | t value | p-value | q-value |
| --- | --- | --- | --- | --- | --- | --- |
| (Intercept) | 1.06 | 0.03 | 64.01 | 40.47 | 0.00 | 0 |
| oxytocin | -0.02 | 0.04 | 64.01 | -0.52 | 0.60 | 1 |
| time | 0.00 | 0.01 | 41.00 | -0.27 | 0.78 | 1 |
| oxytocin×time | 0.02 | 0.01 | 41.00 | 1.34 | 0.19 | 1 |

1.10

1.05

Detachment

oxytocin

placebo oxytocin

1.00

0 1 2 3

time

Figure 2.2.9 The predicted values of the oxytocin main effect model with Detachment as the outcome

## The oxytocin main effect model with Elaboration as the outcome

Table 2.2.10. The oxytocin main effect model with Elaboration as the outcome, N = 43

|  | Estimate | Std. Error | df | t value | p-value | q-value |
| --- | --- | --- | --- | --- | --- | --- |
| (Intercept) | 3.56 | 0.17 | 59.49 | 20.95 | 0.00 | 0 |
| oxytocin | -0.22 | 0.26 | 59.49 | -0.87 | 0.39 | 1 |
| time | 0.00 | 0.05 | 41.00 | -0.05 | 0.96 | 1 |
| oxytocin×time | 0.07 | 0.07 | 41.00 | 0.89 | 0.38 | 1 |

3.75

3.50 oxytocin

Elaboration

placebo oxytocin

3.25

3.00

0 1 2 3

time

Figure 2.2.10 The predicted values of the oxytocin main effect model with Elaboration as the outcome

## The oxytocin main effect model with Fluency as the outcome

Table 2.2.11. The oxytocin main effect model with Fluency as the outcome, N = 43

|  | Estimate | Std. Error | df | t value | p-value | q-value |
| --- | --- | --- | --- | --- | --- | --- |
| (Intercept) | 3.95 | 0.15 | 55.79 | 26.62 | 0.00 | 0 |
| oxytocin | -0.35 | 0.22 | 55.79 | -1.55 | 0.13 | 1 |
| time | 0.03 | 0.04 | 41.00 | 0.77 | 0.45 | 1 |
| oxytocin×time | 0.08 | 0.06 | 41.00 | 1.32 | 0.19 | 1 |

4.2

4.0

3.8

Fluency

oxytocin

placebo oxytocin

3.6

3.4

0 1 2 3

time

Figure 2.2.11 The predicted values of the oxytocin main effect model with Fluency as the outcome

## 2.2.12 The oxytocin main effect model with Gaze as the outcome

Table 2.2.12. The oxytocin main effect model with Gaze as the outcome, N = 43

|  | | Estimate | Std. Error | df | t value | p-value |  | q-value |
| --- | --- | --- | --- | --- | --- | --- | --- | --- |
|  |  | (Intercept) 2.91 | 0.23 | 52.9 | 12.42 | 0.00 |  | 0 |
|  |  | oxytocin 0.06 | 0.35 | 52.9 | 0.18 | 0.86 |  | 1 |
|  |  | time 0.03 | 0.06 | 41.0 | 0.62 | 0.54 |  | 1 |
|  |  | oxytocin×time -0.01 | 0.08 | 41.0 | -0.06 | 0.95 |  | 1 |
| 3.6 |  |  |  |  |  |  |  |  |
|  |  |  |  |  |  |  |  |  |
|  |  |  |  |  |  |  |  |  |
|  |  |  |  |  |  |  |  |  |
| 3.3  3.0  2.7 |  |  |  | | | |  |  |

oxytocin

Gaze

placebo oxytocin

2.4

0 1 2 3

time

Figure 2.2.12 The predicted values of the oxytocin main effect model with Gaze as the outcome

## 2.2.13 The oxytocin main effect model with Hostility as the outcome

Table 2.2.13. The oxytocin main effect model with Hostility as the outcome, N = 43

|  | Estimate | Std. Error | df | t value | p-value | q-value |
| --- | --- | --- | --- | --- | --- | --- |
| (Intercept) | 1.00 | 0.02 | 82 | 54.69 | 0.00 | 0 |
| oxytocin | 0.03 | 0.03 | 82 | 0.96 | 0.34 | 1 |
| time | 0.00 | 0.01 | 82 | 0.00 | 1.00 | 1 |
| oxytocin×time | 0.00 | 0.01 | 82 | 0.23 | 0.82 | 1 |

1.08

1.05

oxytocin

Hostility

1.02 placebo

oxytocin

0.99

0.96

0 1 2 3

time

Figure 2.2.13 The predicted values of the oxytocin main effect model with Hostility as the outcome

## The oxytocin main effect model with Initiation as the outcome

Table 2.2.14. The oxytocin main effect model with Initiation as the outcome, N = 43 Estimate Std. Error df t value p-value q-value

(Intercept) 2.94 0.21 69.72 14.18 0.00 0

oxytocin -0.24 0.31 69.72 -0.77 0.44 1

time -0.07 0.07 41.00 -0.89 0.38 1

oxytocin×time 0.10 0.11 41.00 0.90 0.37 1

3.4

3.2

3.0

oxytocin

Initiation

2.8 placebo

oxytocin

2.6

2.4

2.2

0 1 2 3

time

Figure 2.2.14 The predicted values of the oxytocin main effect model with Initiation as the outcome

## The oxytocin main effect model with Intrusiveness as the outcome

Table 2.2.15. The oxytocin main effect model with Intrusiveness as the outcome, N = 43

|  | Estimate | Std. Error | df | t value | p-value | q-value |
| --- | --- | --- | --- | --- | --- | --- |
| (Intercept) | 1.15 | 0.07 | 57.53 | 16.97 | 0.00 | 0 |
| oxytocin | 0.08 | 0.10 | 57.53 | 0.81 | 0.42 | 1 |
| time | 0.01 | 0.02 | 41.00 | 0.37 | 0.71 | 1 |
| oxytocin×time | -0.02 | 0.03 | 41.00 | -0.76 | 0.45 | 1 |

1.3

1.2

Intrusiveness

oxytocin

placebo oxytocin

1.1

1.0

0 1 2 3

time

Figure 2.2.15 The predicted values of the oxytocin main effect model with Intrusiveness as the outcome

## 2.2.16 The oxytocin main effect model with Lead Expert as the outcome

Table 2.2.16. The oxytocin main effect model with Lead Expert as the outcome, N = 43

|  | Estimate | Std. Error | df | t value | p-value | q-value |
| --- | --- | --- | --- | --- | --- | --- |
| (Intercept) | 3.34 | 0.14 | 58.25 | 23.81 | 0.00 | 0 |
| oxytocin | 0.12 | 0.21 | 58.25 | 0.59 | 0.56 | 1 |
| time | 0.03 | 0.04 | 41.00 | 0.64 | 0.53 | 1 |
| oxytocin×time | -0.04 | 0.06 | 41.00 | -0.72 | 0.48 | 1 |

3.8

3.6

3.4

Lead Expert

oxytocin

placebo oxytocin

3.2

0 1 2 3

time

Figure 2.2.16 The predicted values of the oxytocin main effect model with Lead Expert as the outcome

## 2.2.17 The oxytocin main effect model with Lead Patient as the outcome

Table 2.2.17. The oxytocin main effect model with Lead Patient as the outcome, N = 43

|  | Estimate | Std. Error | df | t value | p-value | q-value |
| --- | --- | --- | --- | --- | --- | --- |
| (Intercept) | 2.92 | 0.14 | 53.1 | 20.33 | 0.00 | 0 |
| oxytocin | -0.28 | 0.22 | 53.1 | -1.28 | 0.21 | 1 |
| time | -0.02 | 0.03 | 41.0 | -0.60 | 0.55 | 1 |
| oxytocin×time | 0.05 | 0.05 | 41.0 | 1.02 | 0.31 | 1 |

3.00

2.75

Lead Patient

oxytocin

placebo oxytocin

2.50

0 1 2 3

time

Figure 2.2.17 The predicted values of the oxytocin main effect model with Lead Patient as the outcome

## 2.2.18 The oxytocin main effect model with Mismatched Affect as the outcome

Table 2.2.18. The oxytocin main effect model with Mismatched Affect as the outcome, N = 43

|  | Estimate | Std. Error | df | t value | p-value | q-value |
| --- | --- | --- | --- | --- | --- | --- |
| (Intercept) | 1.24 | 0.06 | 62.23 | 20.05 | 0.00 | 0 |
| oxytocin | -0.12 | 0.09 | 62.23 | -1.30 | 0.20 | 1 |
| time | -0.02 | 0.02 | 41.00 | -0.90 | 0.37 | 1 |
| oxytocin×time | 0.00 | 0.03 | 41.00 | 0.15 | 0.89 | 1 |

1.3

1.2

Mismatched Affect

1.1

oxytocin

placebo oxytocin

1.0

0 1 2 3

time

Figure 2.2.18 The predicted values of the oxytocin main effect model with Mismatched Affect as the outcome

## The oxytocin main effect model with Motivation as the outcome

Table 2.2.19. The oxytocin main effect model with Motivation as the outcome, N = 43 Estimate Std. Error df t value p-value q-value

(Intercept) 3.99 0.16 59.82 25.57 0.00 0.00

oxytocin -0.01 0.23 59.82 -0.05 0.96 1.00

time 0.11 0.05 41.00 2.32 0.03 0.69

oxytocin×time -0.07 0.07 41.00 -0.95 0.35 1.00

4.6

4.4

4.2 oxytocin

Motivation

placebo oxytocin

4.0

3.8

3.6

0 1 2 3

time

Figure 2.2.19 The predicted values of the oxytocin main effect model with Motivation as the outcome

## The oxytocin main effect model with negative affect as the outcome

Table 2.2.20. The oxytocin main effect model with negative affect as the outcome, N = 43

|  | Estimate | Std. Error | df | t value | p-value | q-value |
| --- | --- | --- | --- | --- | --- | --- |
| (Intercept) | 1.41 | 0.13 | 71.36 | 10.82 | 0.00 | 0 |
| oxytocin | 0.40 | 0.20 | 71.36 | 2.03 | 0.05 | 1 |
| time | 0.03 | 0.05 | 41.00 | 0.67 | 0.50 | 1 |
| oxytocin×time | -0.13 | 0.07 | 41.00 | -1.74 | 0.09 | 1 |

2.0

1.8

1.6

negative affect

oxytocin

placebo oxytocin

1.4

1.2

0 1 2 3

time

Figure 2.2.20 The predicted values of the oxytocin main effect model with negative affect as the outcome

## 2.2.21 The oxytocin main effect model with Positive Affect as the outcome

Table 2.2.21. The oxytocin main effect model with Positive Affect as the outcome, N = 43

|  | Estimate | Std. Error | df | t value | p-value | q-value |
| --- | --- | --- | --- | --- | --- | --- |
| (Intercept) | 1.78 | 0.13 | 53.64 | 13.25 | 0.00 | 0 |
| oxytocin | -0.31 | 0.20 | 53.64 | -1.55 | 0.13 | 1 |
| time | 0.05 | 0.03 | 41.00 | 1.54 | 0.13 | 1 |
| oxytocin×time | 0.04 | 0.05 | 41.00 | 0.86 | 0.40 | 1 |

2.00

1.75

Positive Affect

oxytocin

placebo oxytocin

1.50

1.25

0 1 2 3

time

Figure 2.2.21 The predicted values of the oxytocin main effect model with Positive Affect as the outcome

## 2.2.22 The oxytocin main effect model with Persistence as the outcome

Table 2.2.22. The oxytocin main effect model with Persistence as the outcome, N = 43

|  | Estimate | Std. Error | df | t value | p-value | q-value |
| --- | --- | --- | --- | --- | --- | --- |
| (Intercept) | 4.61 | 0.12 | 63.03 | 39.78 | 0.00 | 0 |
| oxytocin | 0.03 | 0.17 | 63.03 | 0.17 | 0.87 | 1 |
| time | 0.03 | 0.04 | 41.00 | 0.76 | 0.45 | 1 |
| oxytocin×time | -0.04 | 0.06 | 41.00 | -0.77 | 0.45 | 1 |

4.9

4.8

4.7

Persistence

4.6

oxytocin

placebo oxytocin

4.5

4.4

0 1 2 3

time

Figure 2.2.22 The predicted values of the oxytocin main effect model with Persistence as the outcome

## 2.2.23 The oxytocin main effect model with Reciprocity as the outcome

Table 2.2.23. The oxytocin main effect model with Reciprocity as the outcome, N = 43

|  | Estimate | Std. Error | df | t value | p-value |  | q-value |
| --- | --- | --- | --- | --- | --- | --- | --- |
|  | (Intercept) 4.15 | 0.13 | 57.04 | 31.91 | 0.00 |  | 0 |
|  | oxytocin -0.21 | 0.20 | 57.04 | -1.09 | 0.28 |  | 1 |
|  | time 0.02 | 0.04 | 41.00 | 0.58 | 0.56 |  | 1 |
|  | oxytocin×time 0.03 | 0.05 | 41.00 | 0.48 | 0.63 |  | 1 |
| 4.50 |  |  |  |  |  |  |  |
|  |  |  |  |  |  |  |  |
|  |  |  | | | |  |  |
| 4.25  4.00 |  |  | | | |  |  |

oxytocin

Reciprocity

placebo oxytocin

3.75

0 1 2 3

time

Figure 2.2.23 The predicted values of the oxytocin main effect model with Reciprocity as the outcome

## 2.2.24 The oxytocin main effect model with Silence as the outcome

Table 2.2.24. The oxytocin main effect model with Silence as the outcome, N = 43 Estimate Std. Error df t value p-value q-value

(Intercept) 1.42 0.11 51.79 13.39 0.00 0

oxytocin -0.02 0.16 51.79 -0.13 0.90 1

time -0.01 0.02 41.00 -0.38 0.71 1

oxytocin×time 0.02 0.04 41.00 0.65 0.52 1

1.7

1.6

1.5

Silence

1.4

oxytocin

placebo oxytocin

1.3

1.2

0 1 2 3

time

Figure 2.2.24 The predicted values of the oxytocin main effect model with Silence as the outcome

## 2.2.25 The oxytocin main effect model with Synchrony as the outcome

Table 2.2.25. The oxytocin main effect model with Synchrony as the outcome, N = 43

|  | Estimate | Std. Error | df | t value | p-value | q-value |
| --- | --- | --- | --- | --- | --- | --- |
| (Intercept) | 4.05 | 0.13 | 61.1 | 30.46 | 0.00 | 0 |
| oxytocin | -0.25 | 0.20 | 61.1 | -1.25 | 0.22 | 1 |
| time | 0.02 | 0.04 | 41.0 | 0.52 | 0.61 | 1 |
| oxytocin×time | 0.04 | 0.06 | 41.0 | 0.67 | 0.51 | 1 |

4.25

4.00 oxytocin

Synchrony

placebo oxytocin

3.75

3.50

0 1 2 3

time

Figure 2.2.25 The predicted values of the oxytocin main effect model with Synchrony as the outcome

## 2.2.26 The oxytocin main effect model with Tension as the outcome

Table 2.2.26. The oxytocin main effect model with Tension as the outcome, N = 43

|  | | Estimate | Std. Error | df | t value | p-value |  | q-value |
| --- | --- | --- | --- | --- | --- | --- | --- | --- |
|  |  | (Intercept) 1.43 | 0.12 | 57.2 | 12.33 | 0.00 |  | 0 |
|  |  | oxytocin 0.20 | 0.17 | 57.2 | 1.15 | 0.25 |  | 1 |
|  |  | time 0.00 | 0.03 | 41.0 | 0.07 | 0.94 |  | 1 |
|  |  | oxytocin×time -0.08 | 0.05 | 41.0 | -1.69 | 0.10 |  | 1 |
| 1.9 |  |  |  |  |  |  |  |  |
| 1.7  1.5  1.3 |  |  |  | | | |  |  |

oxytocin

Tension

placebo oxytocin

1.1

0 1 2 3

time

Figure 2.2.26 The predicted values of the oxytocin main effect model with Tension as the outcome

## 2.2.27 The oxytocin main effect model with Withdrawal as the outcome

Table 2.2.27. The oxytocin main effect model with Withdrawal as the outcome, N = 43

|  | Estimate | Std. Error | df | t value | p-value | q-value |
| --- | --- | --- | --- | --- | --- | --- |
| (Intercept) | 1.40 | 0.08 | 77.1 | 16.92 | 0.00 | 0 |
| oxytocin | 0.06 | 0.12 | 77.1 | 0.49 | 0.63 | 1 |
| time | -0.03 | 0.03 | 41.0 | -0.89 | 0.38 | 1 |
| oxytocin×time | 0.00 | 0.05 | 41.0 | 0.07 | 0.94 | 1 |

1.6

1.5

1.4

Withdrawal

oxytocin

placebo oxytocin

1.3

1.2

0 1 2 3

time

Figure 2.2.27 The predicted values of the oxytocin main effect model with Withdrawal as the outcome

# The oxytocin main effect models: the PANSS scores as the outcomes

This section provides the results for each of the PANSS scores as the outcome in the models.

## The oxytocin main effect model with PANSS total as the outcome

Table 2.3.1. The oxytocin main effect model with PANSS total as the outcome, N = 51

|  | Estimate | Std. Error | df | t value | p-value | q-value |
| --- | --- | --- | --- | --- | --- | --- |
| (Intercept) | 64.90 | 2.77 | 57.24 | 23.43 | 0.00 | 0 |
| oxytocin | 4.61 | 3.96 | 57.47 | 1.16 | 0.25 | 1 |
| time | -2.72 | 0.56 | 137.58 | -4.83 | 0.00 | 0 |
| oxytocin×time | 0.67 | 0.81 | 138.23 | 0.82 | 0.41 | 1 |

75

70

65 oxytocin

PANSS total

placebo oxytocin

60

55

0 1 2 3

time

Figure 2.3.1 The predicted values of the oxytocin main effect model with PANSS total as the outcome

## The oxytocin main effect model with P (PANSS) as the outcome

Table 2.3.2. The oxytocin main effect model with P (PANSS) as the outcome, N = 51

|  | Estimate | Std. Error | df | t value | p-value | q-value |
| --- | --- | --- | --- | --- | --- | --- |
| (Intercept) | 12.52 | 0.90 | 56.35 | 13.98 | 0.00 | 0.00 |
| oxytocin | 1.84 | 1.28 | 56.59 | 1.44 | 0.16 | 1.00 |
| time | -0.91 | 0.19 | 136.29 | -4.87 | 0.00 | 0.00 |
| oxytocin×time | 0.56 | 0.27 | 136.97 | 2.09 | 0.04 | 0.34 |

16

14

oxytocin

P (PANSS)

12 placebo

oxytocin

10

8

0 1 2 3

time

Figure 2.3.2 The predicted values of the oxytocin main effect model with P (PANSS) as the outcome

## The oxytocin main effect model with N (PANSS) as the outcome

Table 2.3.3. The oxytocin main effect model with N (PANSS) as the outcome, N = 51

|  | Estimate | Std. Error | df | t value | p-value | q-value |
| --- | --- | --- | --- | --- | --- | --- |
| (Intercept) | 21.18 | 1.02 | 64.17 | 20.70 | 0.00 | 0 |
| oxytocin | 0.33 | 1.46 | 64.54 | 0.23 | 0.82 | 1 |
| time | -1.16 | 0.26 | 138.93 | -4.55 | 0.00 | 0 |
| oxytocin×time | 0.46 | 0.37 | 139.79 | 1.24 | 0.22 | 1 |

24

22

oxytocin

N (PANSS)

20 placebo

oxytocin

18

16

0 1 2 3

time

Figure 2.3.3 The predicted values of the oxytocin main effect model with N (PANSS) as the outcome

## The oxytocin main effect model with G (PANSS) as the outcome

Table 2.3.4. The oxytocin main effect model with G (PANSS) as the outcome, N = 51

|  | Estimate | Std. Error | df | t value | p-value | q-value |
| --- | --- | --- | --- | --- | --- | --- |
| (Intercept) | 30.99 | 1.40 | 61.04 | 22.08 | 0.00 | 0.00 |
| oxytocin | 2.61 | 2.01 | 61.35 | 1.30 | 0.20 | 1.00 |
| time | -0.75 | 0.32 | 138.31 | -2.32 | 0.02 | 0.19 |
| oxytocin×time | -0.26 | 0.47 | 139.09 | -0.55 | 0.59 | 1.00 |

35.0

32.5

G (PANSS)

30.0

oxytocin

placebo oxytocin

27.5

0 1 2 3

time

Figure 2.3.4 The predicted values of the oxytocin main effect model with G (PANSS) as the outcome

## The oxytocin main effect model with Withdrawal (PANSS) as the outcome

Table 2.3.5. The oxytocin main effect model with Withdrawal (PANSS) as the outcome, N = 51

|  | Estimate | Std. Error | df | t value | p-value | q-value |
| --- | --- | --- | --- | --- | --- | --- |
| (Intercept) | 3.32 | 0.16 | 66.59 | 20.60 | 0.00 | 0 |
| oxytocin | -0.03 | 0.23 | 66.99 | -0.13 | 0.90 | 1 |
| time | -0.19 | 0.04 | 139.43 | -4.52 | 0.00 | 0 |
| oxytocin×time | 0.07 | 0.06 | 140.33 | 1.17 | 0.24 | 1 |

3.6

3.2

Withdrawal (PANSS)

oxytocin

placebo oxytocin

2.8

0 1 2 3

time

Figure 2.3.5 The predicted values of the oxytocin main effect model with Withdrawal (PANSS) as the outcome

## The oxytocin main effect model with Delusion (PANSS) as the outcome

Table 2.3.6. The oxytocin main effect model with Delusion (PANSS) as the outcome, N = 51

|  | Estimate | Std. Error | df | t value | p-value | q-value |
| --- | --- | --- | --- | --- | --- | --- |
| (Intercept) | 1.62 | 0.16 | 53.37 | 10.31 | 0.00 | 0.00 |
| oxytocin | 0.44 | 0.22 | 53.55 | 1.98 | 0.05 | 0.48 |
| time | -0.10 | 0.03 | 135.85 | -3.70 | 0.00 | 0.00 |
| oxytocin×time | 0.07 | 0.04 | 136.40 | 1.75 | 0.08 | 0.74 |

2.0

oxytocin

Delusion (PANSS)

placebo oxytocin

1.5

1.0

0 1 2 3

time

Figure 2.3.6 The predicted values of the oxytocin main effect model with Delusion (PANSS) as the outcome

## The oxytocin main effect model with Anxiety (PANSS) as the outcome

Table 2.3.7. The oxytocin main effect model with Anxiety (PANSS) as the outcome, N = 51

|  | Estimate | Std. Error | df | t value | p-value | q-value |
| --- | --- | --- | --- | --- | --- | --- |
| (Intercept) | 2.47 | 0.17 | 69.29 | 14.27 | 0.00 | 0.00 |
| oxytocin | 0.30 | 0.25 | 69.74 | 1.20 | 0.24 | 1.00 |
| time | -0.13 | 0.05 | 139.89 | -2.62 | 0.01 | 0.09 |
| oxytocin×time | -0.07 | 0.07 | 140.83 | -0.98 | 0.33 | 1.00 |

2.8

Anxiety (PANSS)

oxytocin

placebo oxytocin

2.0

0 1 2 3

time

Figure 2.3.7 The predicted values of the oxytocin main effect model with Anxiety (PANSS) as the outcome

## 2.3.8 The oxytocin main effect model with Disorganization (PANSS) as the out- come

Table 2.3.8. The oxytocin main effect model with Disorganization (PANSS) as the outcome, N = 51

|  | Estimate | Std. Error | df | t value | p-value | q-value |
| --- | --- | --- | --- | --- | --- | --- |
| (Intercept) | 2.01 | 0.12 | 64.46 | 17.31 | 0.00 | 0 |
| oxytocin | -0.01 | 0.17 | 64.89 | -0.08 | 0.94 | 1 |
| time | -0.03 | 0.03 | 136.17 | -0.85 | 0.40 | 1 |
| oxytocin×time | -0.03 | 0.05 | 137.15 | -0.69 | 0.49 | 1 |

2.2

2.0

Disorganization (PANSS)

oxytocin

placebo oxytocin

1.8

1.6

0 1 2 3

time

Figure 2.3.8 The predicted values of the oxytocin main effect model with Disorganization (PANSS) as the outcome

## 2.3.9 The oxytocin main effect model with Poor control (PANSS) as the outcome

Table 2.3.9. The oxytocin main effect model with Poor control (PANSS) as the outcome, N = 51

|  | Estimate | Std. Error | df | t value | p-value | q-value |
| --- | --- | --- | --- | --- | --- | --- |
| (Intercept) | 1.55 | 0.09 | 69.95 | 16.33 | 0.00 | 0 |
| oxytocin | 0.00 | 0.14 | 70.46 | -0.01 | 0.99 | 1 |
| time | -0.04 | 0.03 | 137.52 | -1.55 | 0.12 | 1 |
| oxytocin×time | 0.01 | 0.04 | 138.56 | 0.25 | 0.80 | 1 |

1.8

1.6

Poor control (PANSS)

oxytocin

placebo oxytocin

1.4

1.2

0 1 2 3

time

Figure 2.3.9 The predicted values of the oxytocin main effect model with Poor control (PANSS) as the outcome

# The social treatment main effect models

To estimate the effects over time of social treatments (without oxytocin), mixed effects mo dels were used for each of the five social i nteraction f actors, t he i ndividual s ocial interaction s cores, a nd t he PANSS s cores as the outcome. In these models, multiple measurements (one per time point) for each patient were modeled as random effects. The coefficients of interest are the change in the effects of social treatments over time, which is represented as the time interaction. I.e., social treatments × time. The p-values due to multiple testing were adjusted using Bonferroni corrrection for each group of outcomes (the set of social interaction factors, the set of the individual social interaction scores, and the set of PANSS-related scores) and the corrected values are referred to as the ‘q-values.’

# The social treatment main effect m odels: t he fi ve so cial inter- action factors as the outcomes

This section provides the results for each of the five social interaction factors—initiation, synchrony, positive affects, tension, a nd withdrawal—as t he o utcome in t he models.

## The social treatment main effect model with Factor 1 (synchrony) total as the outcome

Table 3.1.1. The social treatment main effect model with Factor 1 (synchrony) total as the outcome, N = 43

|  | Estimate | Std. Error | df | t value | p-value | q-value |
| --- | --- | --- | --- | --- | --- | --- |
| (Intercept) | -0.01 | 0.22 | 60.96 | -0.07 | 0.95 | 1 |
| social treatment | -0.16 | 0.31 | 60.96 | -0.52 | 0.61 | 1 |
| time | 0.06 | 0.07 | 41.00 | 0.86 | 0.39 | 1 |
| social treatment×time | 0.02 | 0.09 | 41.00 | 0.26 | 0.79 | 1 |

0.6

0.3

Factor 1 (synchrony) total

0.0

social

supportive psychotherapy

social

−0.3

−0.6

0 1 2 3

time

Figure 3.1.1 The predicted values of the social treatment main effect model with Factor 1 (synchrony) total as the outcome

## The social treatment main effect model with Factor 2 (initiation) total as the outcome

Table 3.1.2. The social treatment main effect model with Factor 2 (initiation) total as the outcome, N = 43

|  | Estimate | Std. Error | df | t value | p-value | q-value |
| --- | --- | --- | --- | --- | --- | --- |
| (Intercept) | 0.01 | 0.22 | 56.92 | 0.05 | 0.96 | 1 |
| social treatment | -0.05 | 0.31 | 56.92 | -0.16 | 0.87 | 1 |
| time | 0.00 | 0.06 | 41.00 | -0.05 | 0.96 | 1 |
| social treatment×time | 0.04 | 0.09 | 41.00 | 0.51 | 0.61 | 1 |

0.50

0.25

Factor 2 (initiation) total

0.00

social

supportive psychotherapy

social skills training

−0.25

−0.50

0 1 2 3

time

Figure 3.1.2 The predicted values of the social skills training main effect model with Factor 2 (initiation) total as the outcome

## The social skills training main effect model with Factor 3 (tension) total as the outcome

Table 3.1.3. The social skills training main effect model with Factor 3 (tension) total as the outcome, N = 43

|  | Estimate | Std. Error | df | t value | p-value | q-value |
| --- | --- | --- | --- | --- | --- | --- |
| (Intercept) | -0.12 | 0.21 | 63.57 | -0.56 | 0.58 | 1 |
| social treatment | 0.52 | 0.30 | 63.57 | 1.73 | 0.09 | 1 |
| time | -0.02 | 0.07 | 41.00 | -0.34 | 0.74 | 1 |
| social treatment×time | -0.12 | 0.10 | 41.00 | -1.30 | 0.20 | 1 |

0.5

social skills training

Factor 3 (tension) total

0.0

supportive psychotherapy

social skills training

−0.5

0 1 2 3

time

Figure 3.1.3 The predicted values of the social skills training main effect model with Factor 3 (tension) total as the outcome

## The social skills training treatment main effect model with Factor 4 (withdrawal) total as the outcome

Table 3.1.4. The social skills training treatment main effect model with Factor 4 (withdrawal) total as the outcome, N = 43

|  | Estimate | Std. Error | df | t value | p-value | q-value |
| --- | --- | --- | --- | --- | --- | --- |
| (Intercept) | -0.25 | 0.21 | 64.68 | -1.18 | 0.24 | 1 |
| social treatment | 0.53 | 0.30 | 64.68 | 1.78 | 0.08 | 1 |
| time | 0.01 | 0.07 | 41.00 | 0.16 | 0.87 | 1 |
| social treatment×time | -0.09 | 0.10 | 41.00 | -0.90 | 0.37 | 1 |

0.4

0.0

Factor 4 (withdrawal) total

social skills training supportive psychotherapy social skills training

−0.4

0 1 2 3

time

Figure 3.1.4 The predicted values of the social skills training treatment main effect model with Factor 4 (withdrawal) total as the outcome

## The social skills training treatment main effect model with Factor 5 (Positive affect) total as the outcome

Table 3.1.5. The social skills training treatment main effect model with Factor 5 (Positive affect) total as the outcome, N = 43

|  | Estimate | Std. Error | df | t value | p-value | q-value |
| --- | --- | --- | --- | --- | --- | --- |
| (Intercept) | 0.00 | 0.20 | 57.56 | 0.02 | 0.98 | 1 |
| social treatment | -0.32 | 0.29 | 57.56 | -1.10 | 0.28 | 1 |
| time | 0.05 | 0.06 | 41.00 | 0.87 | 0.39 | 1 |
| social treatment×time | 0.04 | 0.08 | 41.00 | 0.46 | 0.64 | 1 |

0.4

0.0

Factor 5 (Positive affect) total

social

supportive psychotherapy

social

−0.4

0 1 2 3

time

Figure 3.1.5 The predicted values of the social skills training treatment main effect model with Factor 5 (Positive affect) total as the outcome

## The social skills training treatment main effect model with Factor 1 (synchrony) Conflict Interaction as the outcome

Table 3.1.6. The social skills training treatment main effect model with Factor 1 (synchrony) Conflict Interaction as the outcome, N = 43

|  | Estimate | Std. Error | df | t value | p-value | q-value |
| --- | --- | --- | --- | --- | --- | --- |
| (Intercept) | 0.01 | 0.22 | 63.61 | 0.05 | 0.96 | 1 |
| social treatment | -0.26 | 0.31 | 63.61 | -0.83 | 0.41 | 1 |
| time | 0.01 | 0.07 | 41.00 | 0.18 | 0.86 | 1 |
| social treatment×time | 0.11 | 0.10 | 41.00 | 1.07 | 0.29 | 1 |

0.50

0.25

Factor 1 (synchrony) Conflict Interaction

0.00

social

supportive psychotherapy

social

−0.25

−0.50

0 1 2 3

time

Figure 3.1.6 The predicted values of the social treatment main effect model with CIB total as the outcome

## The social skills training treatment main effect model with Factor 2 (initiation) Conflict Interaction as the outcome

Table 3.1.7. The social skills training treatment main effect model with Factor 2 (initiation) Conflict Interaction as the outcome, N = 43

|  | Estimate | Std. Error | df | t value | p-value | q-value |
| --- | --- | --- | --- | --- | --- | --- |
| (Intercept) | -0.05 | 0.22 | 62.7 | -0.25 | 0.81 | 1 |
| social treatment | -0.06 | 0.31 | 62.7 | -0.18 | 0.86 | 1 |
| time | 0.05 | 0.07 | 41.0 | 0.69 | 0.49 | 1 |
| social treatment×time | -0.02 | 0.10 | 41.0 | -0.19 | 0.85 | 1 |

0.3

Factor 2 (initiation) Conflict Interaction

0.0

social

supportive psychotherapy

social

−0.3

−0.6

0 1 2 3

time

Figure 3.1.7 The predicted values of the social treatment main effect model with Factor 1 (synchrony) Conflict Interaction as the outcome

## The social skills training treatment main effect model with Factor 3 (tension) Conflict Interaction as the outcome

Table 3.1.8. The social skills training treatment main effect model with Factor 3 (tension) Conflict Interaction as the outcome, N = 43

|  | Estimate | Std. Error | df | t value | p-value | q-value |
| --- | --- | --- | --- | --- | --- | --- |
| (Intercept) | -0.11 | 0.21 | 72.13 | -0.52 | 0.60 | 1 |
| social treatment | 0.49 | 0.31 | 72.13 | 1.60 | 0.12 | 1 |
| time | -0.02 | 0.08 | 41.00 | -0.19 | 0.85 | 1 |
| social treatment×time | -0.12 | 0.11 | 41.00 | -1.03 | 0.31 | 1 |

0.5

Factor 3 (tension) Conflict Interaction

social

0.0

supportive psychotherapy

social

−0.5

0 1 2 3

time

Figure 3.1.8 The predicted values of the social treatment main effect model with Factor 2 (initiation) Conflict Interaction as the outcome

## The social skills training treatment main effect model with Factor 4 (withdrawal) Conflict Interaction as the outcome

Table 3.1.9. The social skills training treatment main effect model with Factor 4 (withdrawal) Conflict Interaction as the outcome, N = 43

|  | Estimate | Std. Error | df | t value | p-value | q-value |
| --- | --- | --- | --- | --- | --- | --- |
| (Intercept) | -0.33 | 0.21 | 64.1 | -1.57 | 0.12 | 1.00 |
| social treatment | 0.66 | 0.30 | 64.1 | 2.19 | 0.03 | 0.68 |
| time | 0.06 | 0.07 | 41.0 | 0.95 | 0.35 | 1.00 |
| social treatment×time | -0.15 | 0.10 | 41.0 | -1.55 | 0.13 | 1.00 |

0.8

0.4

Factor 4 (withdrawal) Conflict Interaction

0.0

social

supportive psychotherapy

social

−0.4

−0.8

0 1 2 3

time

Figure 3.1.9 The predicted values of the social skills training treatment main effect model with Factor 3 (tension) Conflict Interaction as the outcome

## The social skills training skills training treatment main effect model with Factor 5 (Positive affect) Conflict Interaction as the outcome

Table 3.1.10. The social skills training skills training treatment main effect model with Factor 5 (Positive affect) Conflict Interaction as the outcome, N = 43

|  | Estimate | Std. Error | df | t value | p-value | q-value |
| --- | --- | --- | --- | --- | --- | --- |
| (Intercept) | 0.04 | 0.20 | 58.22 | 0.21 | 0.83 | 1 |
| social treatment | -0.46 | 0.29 | 58.22 | -1.60 | 0.12 | 1 |
| time | 0.09 | 0.06 | 41.00 | 1.57 | 0.12 | 1 |
| social treatment×time | 0.01 | 0.08 | 41.00 | 0.09 | 0.93 | 1 |

0.5

Factor 5 (Positive affect) Conflict Interaction

social

0.0

supportive psychotherapy

social

−0.5

0 1 2 3

time

Figure 3.1.10 The predicted values of the social treatment main effect model with Factor 4 (withdrawal) Conflict Interaction as the outcome

## The social skills training treatment main effect model with Factor 1 (synchrony) Positive Interaction as the outcome

Table 3.1.11. The social skills training treatment main effect model with Factor 1 (synchrony) Positive Interaction as the outcome, N = 43

|  | Estimate | Std. Error | df | t value | p-value | q-value |
| --- | --- | --- | --- | --- | --- | --- |
| (Intercept) | -0.12 | 0.21 | 73.19 | -0.56 | 0.57 | 1 |
| social treatment | 0.06 | 0.30 | 73.19 | 0.20 | 0.84 | 1 |
| time | 0.16 | 0.08 | 41.00 | 1.98 | 0.05 | 1 |
| social treatment×time | -0.16 | 0.11 | 41.00 | -1.42 | 0.16 | 1 |

0.5

Factor 1 (synchrony) Positive Interaction

social

0.0

supportive psychotherapy

social

−0.5

0 1 2 3

time

Figure 3.1.11 The predicted values of the social treatment main effect model with Factor 5 (Positive affect) Conflict Interaction as the outcome

## The social skills training treatment main effect model with Factor 2 (initiation) Positive Interaction as the outcome

Table 3.1.12. The social skills training treatment main effect model with Factor 2 (initiation) Positive Interaction as the outcome, N = 43

|  | Estimate | Std. Error | df | t value | p-value | q-value |
| --- | --- | --- | --- | --- | --- | --- |
| (Intercept) | 0.11 | 0.21 | 65.18 | 0.50 | 0.62 | 1 |
| social treatment | -0.21 | 0.31 | 65.18 | -0.69 | 0.49 | 1 |
| time | -0.08 | 0.07 | 41.00 | -1.14 | 0.26 | 1 |
| social treatment×time | 0.20 | 0.10 | 41.00 | 1.94 | 0.06 | 1 |

0.4

Factor 2 (initiation) Positive Interaction

social

0.0

supportive psychotherapy

social

−0.4

0 1 2 3

time

Figure 3.1.12 The predicted values of the social treatment main effect model with Factor 1 (synchrony) Positive Interaction as the outcome

## The social treatment main effect model with Factor 3 (tension) Positive Interaction as the outcome

Table 3.1.13. The social treatment main effect model with Factor 3 (tension) Positive Interaction as the outcome, N = 43

|  | Estimate | Std. Error | df | t value | p-value | q-value |
| --- | --- | --- | --- | --- | --- | --- |
| (Intercept) | -0.18 | 0.21 | 67.23 | -0.85 | 0.40 | 1 |
| social treatment | 0.51 | 0.30 | 67.23 | 1.69 | 0.10 | 1 |
| time | 0.02 | 0.07 | 41.00 | 0.32 | 0.75 | 1 |
| social treatment×time | -0.13 | 0.10 | 41.00 | -1.22 | 0.23 | 1 |

0.8

0.4

Factor 3 (tension) Positive Interaction

social

supportive psychotherapy

0.0 social

−0.4

0 1 2 3

time

Figure 3.1.13 The predicted values of the social treatment main effect model with Factor 2 (initiation) Positive Interaction as the outcome

## The social treatment main effect model with Factor 4 (withdrawal) Posi- tive Interaction as the outcome

Table 3.1.14. The social treatment main effect model with Factor 4 (withdrawal) Positive Interaction as the outcome, N = 43

|  | Estimate | Std. Error | df | t value | p-value | q-value |
| --- | --- | --- | --- | --- | --- | --- |
| (Intercept) | -0.24 | 0.21 | 79.31 | -1.14 | 0.26 | 1 |
| social treatment | 0.32 | 0.30 | 79.31 | 1.04 | 0.30 | 1 |
| time | 0.10 | 0.09 | 41.00 | 1.05 | 0.30 | 1 |
| social treatment×time | -0.12 | 0.13 | 41.00 | -0.94 | 0.35 | 1 |

0.50

0.25

Factor 4 (withdrawal) Positive Interaction

0.00

social

supportive psychotherapy

social

−0.25

−0.50

0 1 2 3

time

Figure 3.1.14 The predicted values of the social treatment main effect model with Factor 3 (tension) Positive Interaction as the outcome

## 3.1.15 The social treatment main effect model with Factor 5 (Positive affect) Positive Interaction as the outcome

Table 3.1.15. The social treatment main effect model with Factor 5 (Positive affect) Positive Interaction as the outcome, N = 43

|  | Estimate | Std. Error | df | t value | p-value | q-value |
| --- | --- | --- | --- | --- | --- | --- |
| (Intercept) | 0.00 | 0.20 | 64.79 | 0.01 | 0.99 | 1 |
| social treatment | -0.10 | 0.29 | 64.79 | -0.35 | 0.73 | 1 |
| time | 0.03 | 0.07 | 41.00 | 0.49 | 0.62 | 1 |
| social treatment×time | -0.06 | 0.10 | 41.00 | -0.61 | 0.55 | 1 |

0.3

Factor 5 (Positive affect) Positive Interaction

social

0.0

supportive psychotherapy

social

−0.3

−0.6

0 1 2 3

time

Figure 3.1.15 The predicted values of the social treatment main effect model with Factor 4 (withdrawal) Positive Interaction as the outcome

## 3.1.16 The social treatment main effect model with Factor 1 (synchrony) Sup- portive interaction as the outcome

Table 3.1.16. The social treatment main effect m odel w ith Factor 1 ( synchrony) S upportive I nteracton as the outcome, N = 43

|  | Estimate | Std. Error | df | t value | p-value | q-value |
| --- | --- | --- | --- | --- | --- | --- |
| (Intercept) | 0.20 | 0.22 | 77.95 | 0.93 | 0.36 | 1 |
| social treatment | -0.42 | 0.31 | 77.95 | -1.34 | 0.18 | 1 |
| time | -0.03 | 0.09 | 41.00 | -0.36 | 0.72 | 1 |
| social treatment×time | 0.06 | 0.13 | 41.00 | 0.47 | 0.64 | 1 |

0.4

Factor 1 (synchrony) Supportive interaction

0.0

social

supportive psychotherapy

social

−0.4

0 1 2 3

time

Figure 3.1.16 The predicted values of the social treatment main effect model with Factor 5 (Positive affect) Positive Interaction as the outcome

## 3.1.17 The social skills training treatment main effect model with Factor 2 (initiation) Sup-portive interaction as the outcome

Table 3.1.17. The social skills training treatment main effect model with Factor 2 (initiation) Supportive

interaction as the outcome, N = 43

|  | Estimate | Std. Error | df | t value | p-value | q-value |
| --- | --- | --- | --- | --- | --- | --- |
| (Intercept) | 0.35 | 0.21 | 61.72 | 1.64 | 0.11 | 1 |
| social treatment | -0.49 | 0.30 | 61.72 | -1.64 | 0.11 | 1 |
| time | -0.08 | 0.06 | 41.00 | -1.17 | 0.25 | 1 |
| social treatment×time | 0.05 | 0.09 | 41.00 | 0.54 | 0.59 | 1 |

0.8

0.4

Factor 2 (initiation) Supportive interaction

social

0.0

supportive psychotherapy

social

−0.4

0 1 2 3

time

Figure 3.1.17 The predicted values of the social treatment main effect model with Factor 1 (synchrony) Supportive interaction as the outcome

## 3.1.18 The social skills training treatment main effect model with Factor 3 (EmpathEmoEx) Supportive interaction as the outcome

Table 3.1.18. The social skills training treatment main effect model with Factor 3 (EmpathEmoEx) Supportive interaction as the outcome, N = 43

|  | Estimate | Std. Error | df | t value | p-value | q-value |
| --- | --- | --- | --- | --- | --- | --- |
| (Intercept) | 0.35 | 0.21 | 82 | 1.66 | 0.10 | 1.00 |
| social treatment | -0.27 | 0.30 | 82 | -0.88 | 0.38 | 1.00 |
| time | -0.20 | 0.10 | 82 | -2.02 | 0.05 | 0.99 |
| social treatment×time | 0.11 | 0.14 | 82 | 0.77 | 0.45 | 1.00 |

0.8

0.4

Factor 3 (EmpathEmoEx) Supportive interaction

social

0.0

supportive psychotherapy

social

−0.4

0 1 2 3

time

Figure 3.1.18 The predicted values of the social treatment main effect model with Factor 2 (initiation) Supportive interaction as the outcome

## 3.1.19 The social skills training treatment main effect model with Factor 4 (Positive affect) Supportive interaction as the outcome

Table 3.1.19. The social skills training treatment main effect model with Factor 4 (Positive affect) Supportive interaction as the outcome, N = 43

|  | Estimate | Std. Error | df | t value | p-value | q-value |
| --- | --- | --- | --- | --- | --- | --- |
| (Intercept) | -0.12 | 0.20 | 69.69 | -0.58 | 0.57 | 1.00 |
| social treatment | -0.09 | 0.29 | 69.69 | -0.32 | 0.75 | 1.00 |
| time | 0.16 | 0.07 | 41.00 | 2.14 | 0.04 | 0.81 |
| social treatment×time | -0.16 | 0.10 | 41.00 | -1.51 | 0.14 | 1.00 |

0.8

0.4

Factor 4 (Positive affect) Supportive interaction

social

0.0

supportive psychotherapy

social

−0.4

0 1 2 3

time

Figure 3.1.19 The predicted values of the social skills training treatment main effect model with Factor 3 (EmpathEmoEx) Supportive interaction as the outcome

## 3.1.20 The social skills training treatment main effect model with Factor 5 (Critisism) Sup-portive interaction as the outcome

Table 3.1.20. The social skills training treatment main effect model with Factor 5 (Critisism) Supportive

interaction as the outcome, N = 43

|  | Estimate | Std. Error | df | t value | p-value | q-value |
| --- | --- | --- | --- | --- | --- | --- |
| (Intercept) | 0.01 | 0.22 | 80.31 | 0.03 | 0.97 | 1 |
| social treatment | -0.08 | 0.31 | 80.31 | -0.25 | 0.81 | 1 |
| time | 0.09 | 0.09 | 41.00 | 0.92 | 0.36 | 1 |
| social treatment×time | -0.11 | 0.13 | 41.00 | -0.85 | 0.40 | 1 |

0.4

Factor 5 (Critisism) Supportive interaction

social

0.0

supportive psychotherapy

social

−0.4

0 1 2 3

time

Figure 3.1.20 The predicted values of the social treatment main effect model with Factor 4 (Positive affect) Supportive interaction as the outcome

## 3.1.21 The social skills training treatment main effect model with CIB total as the outcome

Table 3.1.21. The social skills training treatment main effect model with CIB total as the outcome, N = 43

|  | Estimate | Std. Error | df | t value | p-value | q-value |
| --- | --- | --- | --- | --- | --- | --- |
| (Intercept) | 19.97 | 1.99 | 52.77 | 10.06 | 0.00 | 0 |
| social treatment | -1.30 | 2.84 | 52.77 | -0.46 | 0.65 | 1 |
| time | 0.22 | 0.47 | 41.00 | 0.46 | 0.65 | 1 |
| social treatment×time | 0.46 | 0.68 | 41.00 | 0.69 | 0.50 | 1 |

25.0

22.5

20.0

CIB total

social

supportive psychotherapy

social

17.5

15.0

0 1 2 3

time

Figure 3.1.21 The predicted values of the social skills training treatment main effect model with Factor 5 (Critisism) Supportive interaction as the outcome

# The social skills training treatment main effect models: the individual social interaction scores as the outcomes

This section provides the results for each of the individual social skills training interaction scores as the outcome in the models.

## The social skills training treatment main effect model with Acknowledgment as the out-come

Table 3.2.1. The social skills training treatment main effect model with Acknowledgment as the outcome, N

= 43

|  | Estimate | Std. Error | df | t value | p-value | q-value |
| --- | --- | --- | --- | --- | --- | --- |
| (Intercept) | 4.06 | 0.19 | 62.07 | 21.78 | 0.00 | 0 |
| social treatment | -0.23 | 0.27 | 62.07 | -0.85 | 0.40 | 1 |
| time | 0.05 | 0.06 | 41.00 | 0.87 | 0.39 | 1 |
| social treatment×time | 0.00 | 0.08 | 41.00 | -0.03 | 0.97 | 1 |

4.6

4.4

4.2

Acknowledgment

4.0

social

supportive psychotherapy

social

3.8

3.6

3.4

0 1 2 3

time

Figure 3.2.1 The predicted values of the social treatment main effect model with Acknowledgment as the outcome

## The social skills training treatment main effect model with Alert as the outcome

Table 3.2.2. The social skills training treatment main effect model with Alert as the outcome, N = 43

|  | Estimate | Std. Error | df | t value | p-value | q-value |
| --- | --- | --- | --- | --- | --- | --- |
| (Intercept) | 3.01 | 0.24 | 53.09 | 12.39 | 0.00 | 0 |
| social treatment | -0.31 | 0.35 | 53.09 | -0.89 | 0.38 | 1 |
| time | 0.03 | 0.06 | 41.00 | 0.47 | 0.64 | 1 |
| social treatment×time | 0.03 | 0.08 | 41.00 | 0.30 | 0.77 | 1 |

3.6

3.2

social

supportive psychotherapy

Alert

2.8 social

2.4

0 1 2 3

time

Figure 3.2.2 The predicted values of the social treatment main effect model with Alert as the outcome

## The social skills training treatment main effect model with Anger as the outcome

Table 3.2.3. The social skills training treatment main effect model with Anger as the outcome, N = 43

|  | Estimate | Std. Error | df | t value | p-value | q-value |
| --- | --- | --- | --- | --- | --- | --- |
| (Intercept) | 1.08 | 0.04 | 80.79 | 25.23 | 0.00 | 0 |
| social treatment | 0.00 | 0.06 | 80.79 | -0.06 | 0.95 | 1 |
| time | 0.01 | 0.02 | 41.00 | 0.67 | 0.51 | 1 |
| social treatment×time | 0.00 | 0.03 | 41.00 | -0.08 | 0.94 | 1 |

1.20

1.15

social

Anger

1.10 supportive psychotherapy

social

1.05

1.00

0 1 2 3

time

Figure 3.2.3 The predicted values of the social treatment main effect model with Anger as the outcome

## The social skills training treatment main effect model with Anxiety as the outcome

Table 3.2.4. The social skills training treatment main effect model with Anxiety as the outcome, N = 43

|  | Estimate | Std. Error | df | t value | p-value | q-value |
| --- | --- | --- | --- | --- | --- | --- |
| (Intercept) | 1.39 | 0.11 | 56.46 | 12.60 | 0.00 | 0 |
| social treatment | 0.07 | 0.16 | 56.46 | 0.47 | 0.64 | 1 |
| time | 0.00 | 0.03 | 41.00 | 0.08 | 0.93 | 1 |
| social treatment×time | -0.01 | 0.04 | 41.00 | -0.31 | 0.76 | 1 |

1.7

1.6

1.5

social

Anxiety

supportive psychotherapy

1.4 social

1.3

1.2

0 1 2 3

time

Figure 3.2.4 The predicted values of the social treatment main effect model with Anxiety as the outcome

## The social skills training treatment main effect model with Avoidance as the outcome

Table 3.2.5. The social skills training treatment main effect model with Avoidance as the outcome, N = 43

|  | Estimate | Std. Error | df | t value | p-value | q-value |
| --- | --- | --- | --- | --- | --- | --- |
| (Intercept) | 1.06 | 0.05 | 68.39 | 19.94 | 0.00 | 0 |
| social treatment | 0.11 | 0.08 | 68.39 | 1.39 | 0.17 | 1 |
| time | 0.01 | 0.02 | 41.00 | 0.41 | 0.69 | 1 |
| social treatment×time | 0.00 | 0.03 | 41.00 | 0.11 | 0.91 | 1 |

1.3

1.2

social

Avoidance

1.1

supportive psychotherapy

social

1.0

0 1 2 3

time

Figure 3.2.5 The predicted values of the social skills training treatment main effect model with Avoidance as the outcome

## 3.2.6 The social skills training treatment main effect model with Blatant affect as the outcome

Table 3.2.6. The social skills training treatment main effect model with Blatant affect as the outcome, N = 43

|  | Estimate | Std. Error | df | t value | p-value | q-value |
| --- | --- | --- | --- | --- | --- | --- |
| (Intercept) | 2.00 | 0.16 | 48.11 | 12.13 | 0.00 | 0.0 |
| social treatment | 0.02 | 0.24 | 48.11 | 0.07 | 0.95 | 1.0 |
| time | 0.04 | 0.03 | 41.00 | 1.13 | 0.26 | 1.0 |
| social treatment×time | -0.11 | 0.04 | 41.00 | -2.45 | 0.02 | 0.5 |

2.25

2.00

Blatant affect

social

supportive psychotherapy

social

1.75

1.50

0 1 2 3

time

Figure 3.2.6 The predicted values of the social treatment main effect model with Blatant affect as the outcome

## The social skills training treatment main effect model with Constricted as the outcome

Table 3.2.7. The social skills training treatment main effect model with Constricted as the outcome, N = 43

|  | Estimate | Std. Error | df | t value | p-value | q-value |
| --- | --- | --- | --- | --- | --- | --- |
| (Intercept) | 1.98 | 0.16 | 56.9 | 12.47 | 0.00 | 0 |
| social treatment | 0.09 | 0.23 | 56.9 | 0.38 | 0.71 | 1 |
| time | -0.02 | 0.04 | 41.0 | -0.41 | 0.69 | 1 |
| social treatment×time | -0.03 | 0.06 | 41.0 | -0.52 | 0.60 | 1 |

2.4

2.2

social

Constricted

2.0 supportive psychotherapy

social

1.8

1.6

0 1 2 3

time

Figure 3.2.7 The predicted values of the social skills training treatment main effect model with Constricted as the outcome

## The social skills training treatment main effect model with Criticism as the outcome

Table 3.2.8. The social skills training treatment main effect model with Criticism as the outcome, N = 43

|  | Estimate | Std. Error | df | t value | p-value | q-value |
| --- | --- | --- | --- | --- | --- | --- |
| (Intercept) | 1.12 | 0.06 | 81.66 | 19.63 | 0.00 | 0 |
| social treatment | 0.03 | 0.08 | 81.66 | 0.36 | 0.72 | 1 |
| time | 0.03 | 0.03 | 41.00 | 1.26 | 0.21 | 1 |
| social treatment×time | -0.03 | 0.04 | 41.00 | -0.81 | 0.42 | 1 |

1.3

1.2

Criticism

social

supportive psychotherapy

social

1.1

1.0

0 1 2 3

time

Figure 3.2.8 The predicted values of the social skills training treatment main effect model with Criticism as the outcome

## 3.2.9 The social skills training treatment main effect model with Detachment as the outcome

Table 3.2.9. The social skills training treatment main effect model with Detachment as the outcome, N = 43

|  | Estimate | Std. Error | df | t value | p-value | q-value |
| --- | --- | --- | --- | --- | --- | --- |
| (Intercept) | 1.07 | 0.03 | 64.83 | 39.34 | 0.00 | 0 |
| social treatment | -0.04 | 0.04 | 64.83 | -1.14 | 0.26 | 1 |
| time | 0.00 | 0.01 | 41.00 | 0.00 | 1.00 | 1 |
| social treatment×time | 0.01 | 0.01 | 41.00 | 0.83 | 0.41 | 1 |

1.10

1.05

Detachment

social

supportive psychotherapy

social

1.00

0 1 2 3

time

Figure 3.2.9 The predicted values of the social skills training treatment main effect model with Detachment as the outcome

## 3.2.10 The social skills training treatment main effect model with Elaboration as the outcome

Table 3.2.10. The social skills training treatment main effect model with Elaboration as the outcome, N = 43

|  | Estimate | Std. Error | df | t value | p-value | q-value |
| --- | --- | --- | --- | --- | --- | --- |
| (Intercept) | 3.36 | 0.18 | 59.86 | 18.98 | 0.00 | 0 |
| social treatment | 0.19 | 0.25 | 59.86 | 0.76 | 0.45 | 1 |
| time | 0.04 | 0.05 | 41.00 | 0.72 | 0.47 | 1 |
| social treatment×time | -0.02 | 0.07 | 41.00 | -0.29 | 0.77 | 1 |

4.00

3.75

3.50

Elaboration

social

supportive psychotherapy

social

3.25

3.00

0 1 2 3

time

Figure 3.2.10 The predicted values of the social treatment main effect model with Elaboration as the outcome

## 3.2.11 The social skills training treatment main effect model with Fluency as the outcome

Table 3.2.11. The social skills training treatment main effect model with Fluency as the outcome, N = 43

|  | Estimate | Std. Error | df | t value | p-value | q-value |
| --- | --- | --- | --- | --- | --- | --- |
| (Intercept) | 3.86 | 0.16 | 55.88 | 24.52 | 0.00 | 0 |
| social treatment | -0.12 | 0.22 | 55.88 | -0.52 | 0.60 | 1 |
| time | 0.05 | 0.04 | 41.00 | 1.15 | 0.26 | 1 |
| social treatment×time | 0.03 | 0.06 | 41.00 | 0.57 | 0.57 | 1 |

4.2

4.0

social

Fluency

3.8

supportive psychotherapy

social

3.6

3.4

0 1 2 3

time

Figure 3.2.11 The predicted values of the social skills training treatment main effect model with Fluency as the outcome

## 3.2.12 The social skills training treatment main effect model with Gaze as the outcome

Table 3.2.12. The social skills training treatment main effect model with Gaze as the outcome, N = 43

|  | Estimate | Std. Error | df | t value | p-value | q-value |
| --- | --- | --- | --- | --- | --- | --- |
| (Intercept) | 3.13 | 0.24 | 51.05 | 12.93 | 0.00 | 0.0 |
| social treatment | -0.39 | 0.35 | 51.05 | -1.13 | 0.26 | 1.0 |
| time | -0.07 | 0.05 | 41.00 | -1.36 | 0.18 | 1.0 |
| social treatment×time | 0.22 | 0.08 | 41.00 | 2.81 | 0.01 | 0.2 |

3.4

social

Gaze

3.0

supportive psychotherapy

social

2.6

2.2

0 1 2 3

time

Figure 3.2.12 The predicted values of the social skills training treatment main effect model with Gaze as the outcome

## 3.2.13 The social skills training treatment main effect model with Hostility as the outcome

Table 3.2.13. The social skills training treatment main effect model with Hostility as the outcome, N = 43

|  | Estimate | Std. Error | df | t value | p-value | q-value |
| --- | --- | --- | --- | --- | --- | --- |
| (Intercept) | 1.00 | 0.02 | 82 | 52.19 | 0.00 | 0 |
| social treatment | 0.02 | 0.03 | 82 | 0.87 | 0.39 | 1 |
| time | 0.01 | 0.01 | 82 | 1.12 | 0.27 | 1 |
| social treatment×time | -0.02 | 0.01 | 82 | -1.40 | 0.17 | 1 |

1.050

1.025

Hostility

social

supportive psychotherapy

social

1.000

0.975

0 1 2 3

time

Figure 3.2.13 The predicted values of the social skills training treatment main effect model with Hostility as the outcome

## 3.2.14 The social skills training treatment main effect model with Initiation as the outcome

Table 3.2.14. The social skills training treatment main effect model with Initiation as the outcome, N = 43

|  | Estimate | Std. Error | df | t value | p-value | q-value |
| --- | --- | --- | --- | --- | --- | --- |
| (Intercept) | 2.59 | 0.21 | 71.68 | 12.26 | 0.00 | 0 |
| social treatment | 0.49 | 0.30 | 71.68 | 1.63 | 0.11 | 1 |
| time | -0.01 | 0.08 | 41.00 | -0.14 | 0.89 | 1 |
| social treatment×time | -0.02 | 0.11 | 41.00 | -0.18 | 0.86 | 1 |

3.2

2.8

Initiation

social

supportive psychotherapy

social

2.4

0 1 2 3

time

Figure 3.2.14 The predicted values of the social skills training treatment main effect model with Initiation as the outcome

## 3.2.15 The social skills training treatment main effect model with Intrusiveness as the outcome

Table 3.2.15. The social skills training treatment main effect model with Intrusiveness as the outcome, N = 43

|  | Estimate | Std. Error | df | t value | p-value | q-value |
| --- | --- | --- | --- | --- | --- | --- |
| (Intercept) | 1.29 | 0.07 | 59.08 | 19.01 | 0.00 | 0.00 |
| social treatment | -0.22 | 0.10 | 59.08 | -2.23 | 0.03 | 0.79 |
| time | -0.01 | 0.02 | 41.00 | -0.64 | 0.52 | 1.00 |
| social treatment×time | 0.02 | 0.03 | 41.00 | 0.73 | 0.47 | 1.00 |

1.4

1.3

1.2

Intrusiveness

social

supportive psychotherapy

social

1.1

1.0

0 1 2 3

time

Figure 3.2.15 The predicted values of the social skills training treatment main effect model with Intrusiveness as the outcome

## The social skills training treatment main effect model with Lead Expert as the outcome

Table 3.2.16. The social skills training treatment main effect model with Lead Expert as the outcome, N = 43

|  | Estimate | Std. Error | df | t value | p-value | q-value |
| --- | --- | --- | --- | --- | --- | --- |
| (Intercept) | 3.34 | 0.15 | 58.32 | 22.79 | 0.00 | 0 |
| social treatment | 0.11 | 0.21 | 58.32 | 0.53 | 0.60 | 1 |
| time | 0.02 | 0.04 | 41.00 | 0.55 | 0.59 | 1 |
| social treatment×time | -0.03 | 0.06 | 41.00 | -0.56 | 0.58 | 1 |

3.6

social

Lead Expert

3.4 supportive psychotherapy

social

3.2

0 1 2 3

time

Figure 3.2.16 The predicted values of the social skills training treatment main effect model with Lead Expert as the outcome

## The social skills training treatment main effect model with Lead Patient as the outcome

Table 3.2.17. The social skills training treatment main effect model with Lead Patient as the outcome, N = 43

|  | Estimate | Std. Error | df | t value | p-value | q-value |
| --- | --- | --- | --- | --- | --- | --- |
| (Intercept) | 2.89 | 0.15 | 53.25 | 19.17 | 0.00 | 0 |
| social treatment | -0.19 | 0.22 | 53.25 | -0.87 | 0.39 | 1 |
| time | -0.01 | 0.04 | 41.00 | -0.21 | 0.84 | 1 |
| social treatment×time | 0.02 | 0.05 | 41.00 | 0.40 | 0.69 | 1 |

3.2

3.0

2.8

Lead Patient

social

supportive psychotherapy

social

2.6

2.4

0 1 2 3

time

Figure 3.2.17 The predicted values of the social skills training treatment main effect model with Lead Patient as the outcome

## 3.2.18 The social skills training treatment main effect model with Mismatched Affect as the outcome

Table 3.2.18. The social skills training treatment main effect model with Mismatched Affect as the outcome, N = 43

|  | Estimate | Std. Error | df | t value | p-value | q-value |
| --- | --- | --- | --- | --- | --- | --- |
| (Intercept) | 1.18 | 0.07 | 61.4 | 17.99 | 0.00 | 0 |
| social treatment | 0.01 | 0.09 | 61.4 | 0.09 | 0.93 | 1 |
| time | -0.02 | 0.02 | 41.0 | -0.94 | 0.35 | 1 |
| social treatment×time | 0.01 | 0.03 | 41.0 | 0.24 | 0.81 | 1 |

1.3

1.2

Mismatched Affect

social

supportive psychotherapy

social

1.1

1.0

0 1 2 3

time

Figure 3.2.18 The predicted values of the social skills training treatment main effect model with Mismatched Affect as the outcome

## 3.2.19 The social skills training treatment main effect model with Motivation as the outcome

Table 3.2.19. The social skills training treatment main effect model with Motivation as the outcome, N = 43

|  | Estimate | Std. Error | df | t value | p-value | q-value |
| --- | --- | --- | --- | --- | --- | --- |
| (Intercept) | 4.12 | 0.16 | 60.5 | 25.52 | 0.00 | 0 |
| social treatment | -0.29 | 0.23 | 60.5 | -1.25 | 0.22 | 1 |
| time | 0.06 | 0.05 | 41.0 | 1.25 | 0.22 | 1 |
| social treatment×time | 0.03 | 0.07 | 41.0 | 0.50 | 0.62 | 1 |

4.50

4.25

social

Motivation

4.00

supportive psychotherapy

social

3.75

3.50

0 1 2 3

time

Figure 3.2.19 The predicted values of the social skills training treatment main effect model with Motivation as the outcome

## 3.2.20 The social skills training treatment main effect model with negative affect as the outcome

Table 3.2.20. The social skills training treatment main effect model with negative affect as the outcome, N = 43

|  | Estimate | Std. Error | df | t value | p-value | q-value |
| --- | --- | --- | --- | --- | --- | --- |
| (Intercept) | 1.73 | 0.14 | 72.29 | 12.64 | 0.00 | 0 |
| social treatment | -0.31 | 0.20 | 72.29 | -1.56 | 0.12 | 1 |
| time | -0.06 | 0.05 | 41.00 | -1.13 | 0.27 | 1 |
| social treatment×time | 0.07 | 0.07 | 41.00 | 0.97 | 0.34 | 1 |

2.0

1.8

1.6

negative affect

social

supportive psychotherapy

social

1.4

1.2

0 1 2 3

time

Figure 3.2.20 The predicted values of the social skills training treatment main effect model with negative

affect as the outcome

## The social skills training treatment main effect model with Positive Affect as the outcome

Table 3.2.21. The social skills training treatment main effect model with Positive Affect as the outcome, N = 43

|  | Estimate | Std. Error | df | t value | p-value | q-value |
| --- | --- | --- | --- | --- | --- | --- |
| (Intercept) | 1.77 | 0.14 | 53.97 | 12.64 | 0.00 | 0.00 |
| social treatment | -0.26 | 0.20 | 53.97 | -1.29 | 0.20 | 1.00 |
| time | 0.08 | 0.03 | 41.00 | 2.18 | 0.04 | 0.95 |
| social treatment×time | -0.01 | 0.05 | 41.00 | -0.25 | 0.81 | 1.00 |

2.25

2.00

social

Positive Affect

1.75 supportive psychotherapy

social

1.50

1.25

0 1 2 3

time

Figure 3.2.21 The predicted values of the social skills training treatment main effect model with Positive Affect as the outcome

## The social skills training treatment main effect model with Persistence as the outcome

Table 3.2.22. The social skills training treatment main effect model with Persistence as the outcome, N = 43

|  | Estimate | Std. Error | df | t value | p-value | q-value |
| --- | --- | --- | --- | --- | --- | --- |
| (Intercept) | 4.55 | 0.12 | 62.2 | 37.73 | 0.00 | 0 |
| social treatment | 0.16 | 0.17 | 62.2 | 0.93 | 0.35 | 1 |
| time | 0.05 | 0.04 | 41.0 | 1.35 | 0.19 | 1 |
| social treatment×time | -0.08 | 0.05 | 41.0 | -1.58 | 0.12 | 1 |

4.8

social

Persistence

4.6

supportive psychotherapy

social

4.4

0 1 2 3

time

Figure 3.2.22 The predicted values of the social skills training treatment main effect model with Persistence as the outcome

## 3.2.23 The social skills training treatment main effect model with Reciprocity as the outcome

Table 3.2.23. The social skills training treatment main effect model with Reciprocity as the outcome, N = 43

|  | Estimate | Std. Error | df | t value | p-value | q-value |
| --- | --- | --- | --- | --- | --- | --- |
| (Intercept) | 4.05 | 0.14 | 56.78 | 29.49 | 0.00 | 0 |
| social treatment | 0.03 | 0.20 | 56.78 | 0.13 | 0.90 | 1 |
| time | 0.03 | 0.04 | 41.00 | 0.68 | 0.50 | 1 |
| social treatment×time | 0.01 | 0.05 | 41.00 | 0.27 | 0.79 | 1 |

4.5

4.3

social

Reciprocity

4.1

supportive psychotherapy

social

3.9

0 1 2 3

time

Figure 3.2.23 The predicted values of the social skills training treatment main effect model with Reciprocity as the outcome

## 3.2.24 The social skills training treatment main effect model with Silence as the outcome

Table 3.2.24. The social skills training treatment main effect model with Silence as the outcome, N = 43

|  | Estimate | Std. Error | df | t value | p-value | q-value |
| --- | --- | --- | --- | --- | --- | --- |
| (Intercept) | 1.46 | 0.11 | 51.7 | 13.19 | 0.00 | 0 |
| social treatment | -0.10 | 0.16 | 51.7 | -0.61 | 0.54 | 1 |
| time | -0.02 | 0.03 | 41.0 | -0.60 | 0.55 | 1 |
| social treatment×time | 0.03 | 0.04 | 41.0 | 0.93 | 0.36 | 1 |

1.7

1.6

1.5

Silence

1.4

social

supportive psychotherapy

social

1.3

1.2

0 1 2 3

time

Figure 3.2.24 The predicted values of the social skills training treatment main effect model with Silence as the outcome

## 3.2.25 The social skills training treatment main effect model with Synchrony as the outcome

Table 3.2.25. The social skills training treatment main effect model with Synchrony as the outcome, N = 43

|  | Estimate | Std. Error | df | t value | p-value | q-value |
| --- | --- | --- | --- | --- | --- | --- |
| (Intercept) | 3.98 | 0.14 | 60.88 | 28.42 | 0.00 | 0 |
| social treatment | -0.10 | 0.20 | 60.88 | -0.48 | 0.63 | 1 |
| time | 0.03 | 0.04 | 41.00 | 0.77 | 0.44 | 1 |
| social treatment×time | 0.01 | 0.06 | 41.00 | 0.20 | 0.84 | 1 |

4.4

4.2

4.0

Synchrony

social

supportive psychotherapy

social

3.8

3.6

0 1 2 3

time

Figure 3.2.25 The predicted values of the social skills training treatment main effect model with Synchrony as the outcome

## 3.2.26 The social skills training treatment main effect model with Tension as the outcome

Table 3.2.26. The social skills training treatment main effect model with Tension as the outcome, N = 43

|  | Estimate | Std. Error | df | t value | p-value | q-value |
| --- | --- | --- | --- | --- | --- | --- |
| (Intercept) | 1.39 | 0.12 | 57.83 | 11.53 | 0.00 | 0 |
| social treatment | 0.27 | 0.17 | 57.83 | 1.58 | 0.12 | 1 |
| time | 0.00 | 0.03 | 41.00 | 0.00 | 1.00 | 1 |
| social treatment×time | -0.07 | 0.05 | 41.00 | -1.43 | 0.16 | 1 |

1.9

1.7

social

Tension

1.5

supportive psychotherapy

social

1.3

0 1 2 3

time

Figure 3.2.26 The predicted values of the social skills training treatment main effect model with Tension as the outcome

## 3.2.27 The social skills training treatment main effect model with Withdrawal as the outcome

Table 3.2.27. The social skills training treatment main effect model with Withdrawal as the outcome, N = 43

|  | Estimate | Std. Error | df | t value | p-value | q-value |
| --- | --- | --- | --- | --- | --- | --- |
| (Intercept) | 1.33 | 0.08 | 78.2 | 15.80 | 0.00 | 0 |
| social treatment | 0.18 | 0.12 | 78.2 | 1.51 | 0.13 | 1 |
| time | -0.03 | 0.04 | 41.0 | -0.72 | 0.48 | 1 |
| social treatment×time | -0.01 | 0.05 | 41.0 | -0.13 | 0.90 | 1 |

1.6

1.4

Withdrawal

social

supportive psychotherapy

social

1.2

0 1 2 3

time

Figure 3.2.27 The predicted values of the social skills training treatment main effect model with Withdrawal as the outcome

# The social skills training treatment main effect models: the PANSS scores as the outcomes

This section provides the results for each of the PANSS scores as the outcome in the models.

## The social skills training treatment main effect model with PANSS total as the outcome

Table 3.3.1. The social skills training treatment main effect model with PANSS total as the outcome, N = 51

|  | Estimate | Std. Error | df | t value | p-value | q-value |
| --- | --- | --- | --- | --- | --- | --- |
| (Intercept) | 67.63 | 2.87 | 56.70 | 23.56 | 0.00 | 0 |
| social treatment | -0.95 | 4.02 | 56.95 | -0.23 | 0.82 | 1 |
| time | -2.26 | 0.58 | 137.75 | -3.92 | 0.00 | 0 |
| social treatment×time | -0.29 | 0.82 | 137.95 | -0.36 | 0.72 | 1 |

70

65 social

PANSS total

supportive psychotherapy

social

60

55

0 1 2 3

time

Figure 3.3.1 The predicted values of the social skills training treatment main effect model with PANSS total as the outcome

## The social skills training treatment main effect model with P (PANSS) as the outcome

Table 3.3.2. The social skills training treatment main effect model with P (PANSS) as the outcome, N = 51

|  | Estimate | Std. Error | df | t value | p-value | q-value |
| --- | --- | --- | --- | --- | --- | --- |
| (Intercept) | 13.70 | 0.94 | 55.69 | 14.51 | 0.00 | 0.00 |
| social treatment | -0.55 | 1.32 | 55.94 | -0.42 | 0.68 | 1.00 |
| time | -0.60 | 0.19 | 136.39 | -3.10 | 0.00 | 0.02 |
| social treatment×time | -0.08 | 0.27 | 136.61 | -0.30 | 0.77 | 1.00 |

14

social

P (PANSS)

supportive psychotherapy

12 social

10

0 1 2 3

time

Figure 3.3.2 The predicted values of the social skills training treatment main effect model with P (PANSS) as the outcome

## The social skills training treatment main effect model with N (PANSS) as the outcome

Table 3.3.3. The social skills training treatment main effect model with N (PANSS) as the outcome, N = 51

|  | Estimate | Std. Error | df | t value | p-value | q-value |
| --- | --- | --- | --- | --- | --- | --- |
| (Intercept) | 21.46 | 1.05 | 64.12 | 20.51 | 0.00 | 0 |
| social treatment | -0.24 | 1.47 | 64.52 | -0.16 | 0.87 | 1 |
| time | -0.95 | 0.26 | 139.34 | -3.64 | 0.00 | 0 |
| social treatment×time | 0.02 | 0.37 | 139.65 | 0.05 | 0.96 | 1 |

24

22

social

N (PANSS)

supportive psychotherapy

20

social

18

0 1 2 3

time

Figure 3.3.3 The predicted values of the social skills training treatment main effect model with N (PANSS) as the outcome

## The social skills training treatment main effect model with G (PANSS) as the outcome

Table 3.3.4. The social skills training treatment main effect model with G (PANSS) as the outcome, N = 51

|  | Estimate | Std. Error | df | t value | p-value | q-value |
| --- | --- | --- | --- | --- | --- | --- |
| (Intercept) | 32.17 | 1.45 | 60.42 | 22.22 | 0.00 | 0.00 |
| social treatment | 0.19 | 2.03 | 60.74 | 0.09 | 0.93 | 1.00 |
| time | -0.69 | 0.33 | 138.56 | -2.10 | 0.04 | 0.34 |
| social treatment×time | -0.37 | 0.47 | 138.82 | -0.79 | 0.43 | 1.00 |

34

32

G (PANSS)

social

supportive psychotherapy

30 social

28

26

0 1 2 3

time

Figure 3.3.4 The predicted values of the social skills training treatment main effect model with G (PANSS) as the outcome

## The social skills training treatment main effect model with Withdrawal (PANSS) as the outcome

Table 3.3.5. The social skills training treatment main effect model with Withdrawal (PANSS) as the outcome, N = 51

|  | Estimate | Std. Error | df | t value | p-value | q-value |
| --- | --- | --- | --- | --- | --- | --- |
| (Intercept) | 3.32 | 0.16 | 66.79 | 20.26 | 0.00 | 0.00 |
| social treatment | -0.04 | 0.23 | 67.23 | -0.18 | 0.86 | 1.00 |
| time | -0.15 | 0.04 | 139.94 | -3.50 | 0.00 | 0.01 |
| social treatment×time | -0.01 | 0.06 | 140.29 | -0.17 | 0.87 | 1.00 |

3.75

3.50

3.25

Withdrawal (PANSS)

social

supportive psychotherapy

social

3.00

2.75

2.50

0 1 2 3

time

Figure 3.3.5 The predicted values of the social skills training treatment main effect model with Withdrawal (PANSS) as the outcome

## 3.3.6 The social skills training treatment main effect model with Delusion (PANSS) as the outcome

Table 3.3.6. The social skills training treatment main effect model with Delusion (PANSS) as the outcome, N = 51

|  | Estimate | Std. Error | df | t value | p-value | q-value |
| --- | --- | --- | --- | --- | --- | --- |
| (Intercept) | 1.89 | 0.17 | 52.63 | 11.24 | 0.00 | 0.00 |
| social treatment | -0.11 | 0.24 | 52.81 | -0.45 | 0.65 | 1.00 |
| time | -0.07 | 0.03 | 135.95 | -2.49 | 0.01 | 0.13 |
| social treatment×time | 0.00 | 0.04 | 136.11 | 0.12 | 0.91 | 1.00 |

2.25

2.00

1.75

Delusion (PANSS)

social

supportive psychotherapy

social

1.50

1.25

0 1 2 3

time

Figure 3.3.6 The predicted values of the social skills training treatment main effect model with Delusion (PANSS) as the outcome

## The social skills training treatment main effect model with Anxiety (PANSS) as the outcome

Table 3.3.7. The social skills training treatment main effect model with Anxiety (PANSS) as the outcome, N = 51

|  | Estimate | Std. Error | df | t value | p-value | q-value |
| --- | --- | --- | --- | --- | --- | --- |
| (Intercept) | 2.78 | 0.17 | 70.26 | 15.95 | 0.00 | 0.00 |
| social treatment | -0.31 | 0.24 | 70.76 | -1.25 | 0.22 | 1.00 |
| time | -0.14 | 0.05 | 140.54 | -2.80 | 0.01 | 0.05 |
| social treatment×time | -0.04 | 0.07 | 140.93 | -0.62 | 0.54 | 1.00 |

3.0

2.5 social

Anxiety (PANSS)

supportive psychotherapy

social

2.0

0 1 2 3

time

Figure 3.3.7 The predicted values of the social skills training treatment main effect model with Anxiety (PANSS) as the outcome

## The social skills training treatment main effect model with Disorganization (PANSS) as the outcome

Table 3.3.8. The social skills training treatment main effect model with Disorganization (PANSS) as the outcome, N = 51

|  | Estimate | Std. Error | df | t value | p-value | q-value |
| --- | --- | --- | --- | --- | --- | --- |
| (Intercept) | 1.91 | 0.12 | 65.14 | 16.30 | 0.00 | 0 |
| social treatment | 0.20 | 0.16 | 65.62 | 1.19 | 0.24 | 1 |
| time | -0.04 | 0.03 | 136.80 | -1.36 | 0.18 | 1 |
| social treatment×time | 0.00 | 0.05 | 137.20 | 0.10 | 0.92 | 1 |

2.2

2.0 social

Disorganization (PANSS)

supportive psychotherapy

social

1.8

1.6

0 1 2 3

time

Figure 3.3.8 The predicted values of the social skills training treatment main effect model with Disorganization (PANSS) as the outcome

## The social skills training treatment main effect model with Poor control (PANSS) as the outcome

Table 3.3.9. The social skills training treatment main effect model with Poor control (PANSS) as the outcome, N = 51

|  | Estimate | Std. Error | df | t value | p-value | q-value |
| --- | --- | --- | --- | --- | --- | --- |
| (Intercept) | 1.56 | 0.10 | 69.94 | 16.18 | 0.00 | 0 |
| social treatment | -0.02 | 0.14 | 70.50 | -0.15 | 0.88 | 1 |
| time | -0.03 | 0.03 | 137.96 | -0.91 | 0.36 | 1 |
| social treatment×time | -0.03 | 0.04 | 138.41 | -0.62 | 0.53 | 1 |

1.8

1.6

Poor control (PANSS)

social

supportive psychotherapy

social

1.4

1.2

0 1 2 3

time

Figure 3.3.9 The predicted values of the social skills training treatment main effect model with Poor control (PANSS) as the outcome

1. Antipsychotics use in the oxytocin study group vs. placebo group

|  | **Oxytocin**  **(N=22)** | **Placebo**  **(N=25)** |
| --- | --- | --- |
| **Haloperidol (%)** | - | 3 (12) |
| **Perphenazine (%)** | 1 (4.5) | 1 (4) |
| **Levomepromazine (%)** | 1 (4.5) | - |
| **Olanzapine (%)** | 3 (13.6) | 5 (20) |
| **Quetiapine (%)** | 3 (13.6) | 5 (20) |
| **Clozapine (%)** | 7 (31.8) | 5 (20) |
| **Amisulpride (%)** | 4 (18.1) | 5 (20) |
| **Paliperidone (%)** | 2 (9.1) | 3 (12) |
| **Aripiprazole (%)** | 1 (4.5) | 2 (8) |
